# CO/CO and NO/NO coupling at a hidden frustrated Lewis pair template†
†Electronic supplementary information (ESI) available: Experimental and analytical details, spectral data and crystallographic data. CCDC 1506872–1506879. For ESI and crystallographic data in CIF or other electronic format see DOI: 10.1039/c6sc04459j



**DOI:** 10.1039/c6sc04459j

**Published:** 2017-01-04

**Authors:** Tongdao Wang, Long Wang, Constantin G. Daniliuc, Kamil Samigullin, Matthias Wagner, Gerald Kehr, Gerhard Erker

**Affiliations:** a Organisch-Chemisches Institut , Westfälische Wilhelms-Universität Münster , Corrensstraße 40 , 48149 Münster , Germany . Email: erker@uni-muenster.de; b Institut für Anorganische und Analytische Chemie , Goethe-Universität Frankfurt , Max-von-Laue-Straße 7 , 60438 Frankfurt am Main , Germany

## Abstract

*N*-Allyltetramethylpiperidine is readily isomerized to the corresponding enamine by treatment with catalytic amounts of B(C_6_F_5_)_3_. It adds HB(C_6_F_5_)_2_ at the nucleophilic enamine carbon atom to form a C/B Lewis adduct. This reacts with two molar equivalents of carbon monoxide by selective head to tail coupling to give a five-membered C_2_O_2_B heterocycle. In contrast the enamine/HB(C_6_F_5_)_2_ Lewis pair reacts with two molar equiv. of nitric oxide by head to head coupling. This reaction probably proceeds *via* equilibrium with the corresponding vicinal N/B Lewis pair. Most products were characterized by X-ray diffraction.

## Abstract 2

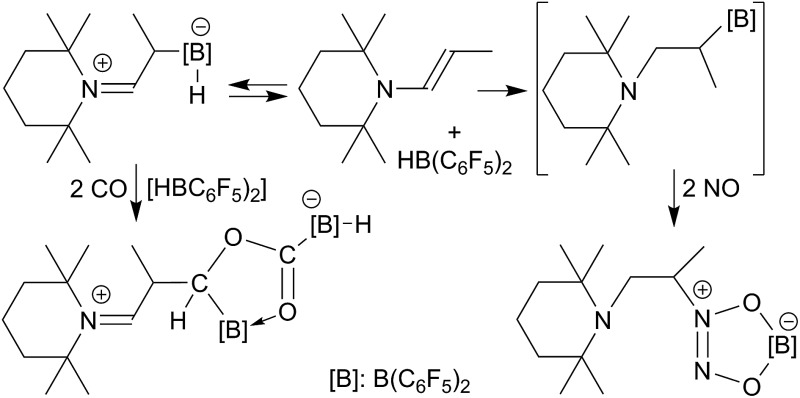
The reversible enamine/HB(C_6_F_5_)_2_ Lewis adduct reacts with CO by selective head to tail coupling whereas it reacts with NO *via* a vicinal N/B Lewis pair to give a head to head NO/NO coupling product.

## Introduction

**Scheme 1:**
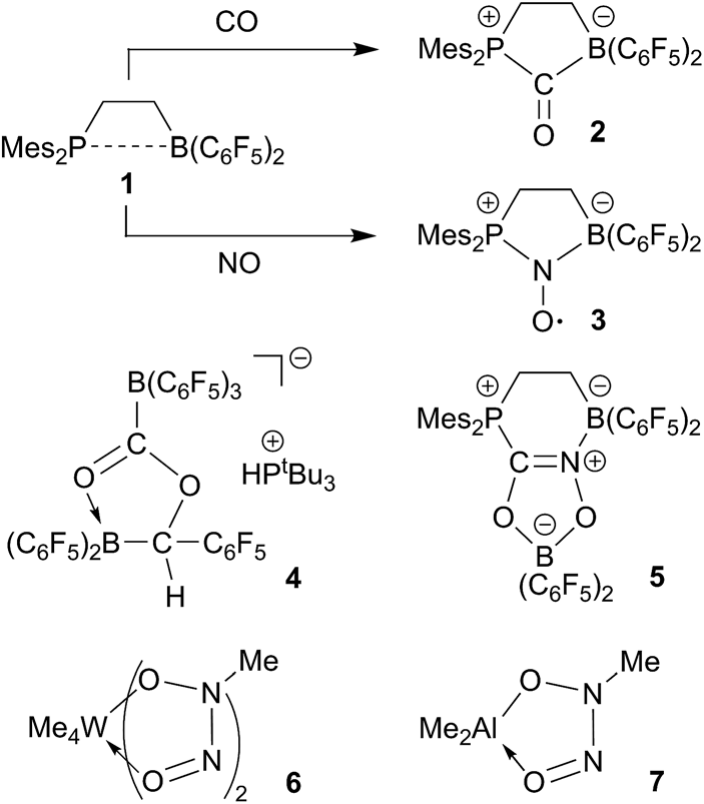


Frustrated Lewis pairs (FLPs) have become well known for their ability of binding and/or activating a variety of small molecules.^1,2^ The reactivity of *e.g.* the ethylene-bridged P/B FLP **1** ^3^ toward carbon monoxide is remarkable. Compound **1** adds both the phosphane Lewis base and the borane Lewis acid to the CO carbon atom to yield the five-membered heterocyclic carbonyl compound **2**.^4^ This behavior of FLP **1** remotely resembles CO/metal coordination chemistry^5^ and in contrast to the conventional role of such P/B pairs as ambiphilic ligands.^6^ Compound **1** reacts analogously with nitric oxide (NO), again serving in a “pseudo-metal like” role, giving the persistent nitroxide radical **3** (see Scheme 1).^7^

There are very few first cases where frustrated Lewis pairs induce coupling of two such element oxides. Stephan *et al.* had found the formation of **4** upon treatment of a 2 : 1 mixture of B(C_6_F_5_)_3_ and P^t^Bu_3_ with CO/H_2_ (ref. 8) and we recently reported the first example of a CO/NO coupling reaction at the FLP **1** and elucidated the pathway of the unprecedented formation of product **5**.^9^ It is also well known that a variety of alkyl or aryl transition metal and main group metal complexes, respectively, undergo reactions involving the head to head coupling of two nitric oxide (NO) molecules to give the respective *O*-metallated *N*-hydrocarbyl-*N*-nitroso-hydroxylaminato metal complexes.^10–15^ The tungsten and aluminum complexes **6** and **7** are representative examples. There have also been early reports about the reaction of Et_3_B with NO to apparently give a Et_2_B–ON(Et)NO type product.^16^

We had previously shown that enamines can react with Piers' borane^17^ by addition of the Lewis acidic borane at the nucleophilic β-carbon atom. Nevertheless some of these systems were H_2_-activators, probably *via* dissociation of the Lewis pair adduct followed by *in situ* generation of the respective vicinal N/B FLP.^18^ We have now conveniently prepared an enamine/HB(C_6_F_5_)_2_ Lewis pair adduct containing the very bulky tetramethylpiperidinyl (TMP) moiety^19^ by an isomerization route starting from the respective allylamine precursor.^20^ The TMP-enamine/HB(C_6_F_5_)_2_ C/B adduct was shown to undergo CO/CO and NO/NO coupling reactions in rather differently oriented pathways. This opposing set of reactions will be described and discussed in this account.

## Results and discussions

### CO/CO coupling with the enamine/HB(C_6_F_5_)_2_ C/B addition product

**Scheme 2:**
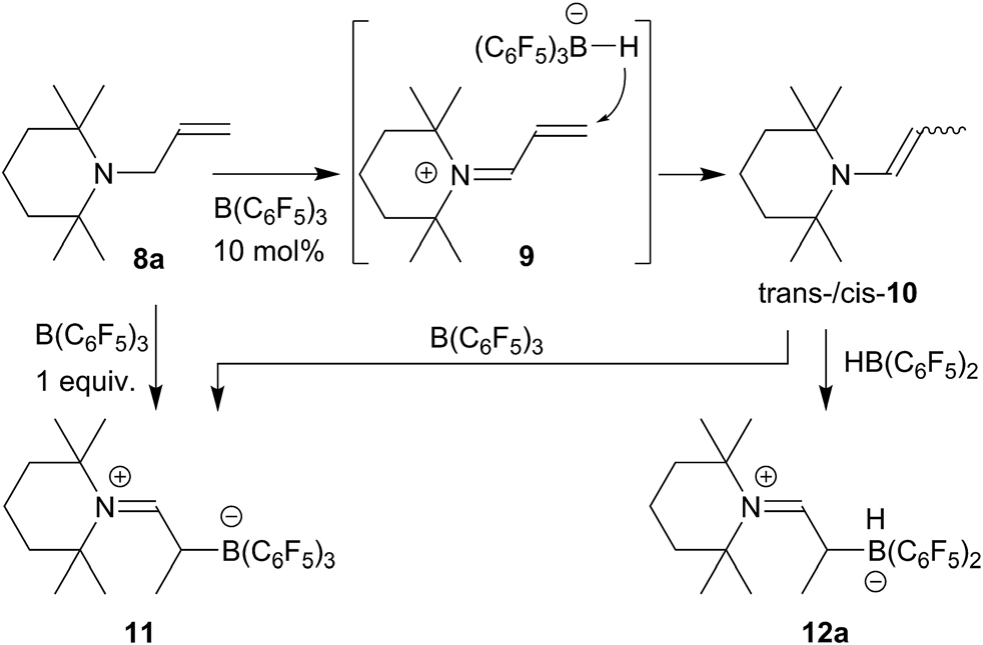


For this part of our study we started from *N*-allyl tetramethylpiperidine (**8a**) which was isomerized to the enamine **10** (*trans*/*cis* mixture of ∼10 : 8) by treatment with a catalytic quantity (10 mol%) of the strong boron Lewis acid B(C_6_F_5_)_3_ (toluene, r.t., 2 d).^21^ The *trans*-/*cis*-enamine mixture was isolated by distillation. Compound *trans*-**10** shows an olefinic [N]–CH = ^1^H NMR resonance at *δ* 5.75 ppm (dq, ^3^ *J*_HH_ = 13.4 Hz, ^4^ *J*_HH_ = 1.6 Hz), whereas *cis*-**10** features the respective olefinic resonance at *δ* 5.68 ppm (dq, ^3^ *J*_HH_ = 7.8 Hz, ^4^ *J*_HH_ = 1.8 Hz). We assume a reaction pathway (see Scheme 2) that involves hydride abstraction^18,22^ in the α-position to the amine to give the conjugated iminium/hydridoborate salt **9** followed by H^–^ addition to the terminal =CH_2_ group.

The enamine isomers **10** rapidly add B(C_6_F_5_)_3_ to the enamine carbon atom^18^ (*n*-pentane, r.t., 30 min) to give the zwitterionic adduct **11** (isolated in 93% after workup). Compound **11** can also be obtained directly from the allylamine **8a** by treatment with a stoichiometric quantity of B(C_6_F_5_)_3_ (toluene, r.t., 30 min); in this case *in situ* generation of the enamine is assumed. We isolated compound **11** in 81% from this stoichiometric reaction. Compound **11** shows a ^11^B NMR resonance at *δ* –11.9 ppm and a typical set of ^19^F NMR signals of *o*,*p*,*m*-fluorines of the three C_6_F_5_-groups at boron with Δ*δ* ^19^F_*m*,*p*_ = 4.5 ppm. The ^1^H/^13^C NMR iminium [N]=CH– resonances were located at *δ* 8.74/*δ* 189.3 ppm, respectively. Compound **11** was also characterized by X-ray diffraction. The structure is depicted in the ESI.†

**Fig. 1:**
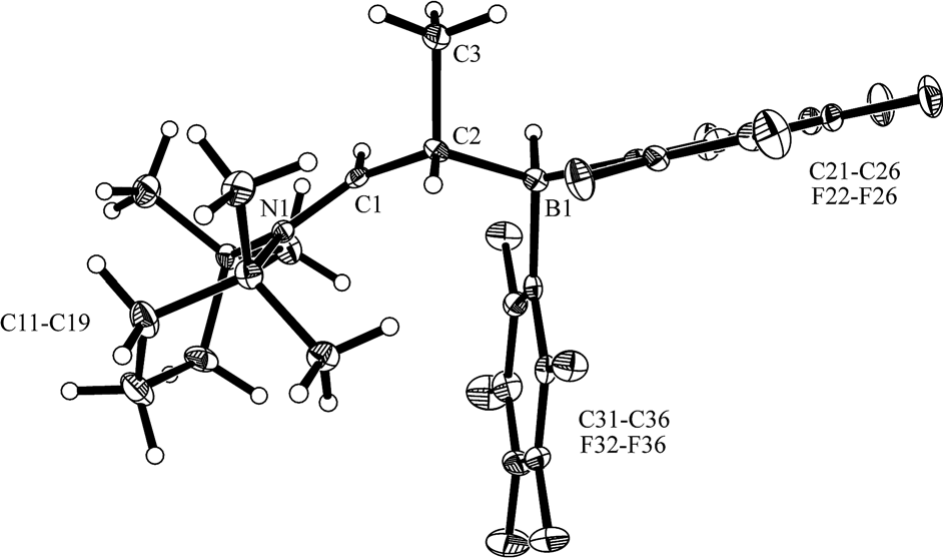
The molecular structure of the enamine/HB(C_6_F_5_)_2_ addition product **12a** (thermal ellipsoids are shown at the 50% probability level). Selected bond lengths (Å) and angles (°): N1–C1 1.299(2), C1–C2 1.463(2), C2–C3 1.549(2), C2–B1 1.682(3), B1–C21 1.636(3), B1–C31 1.649(3); N1–C1–C2 131.8(2), C1–C2–C3 108.6(2), C1–C2–B1 106.2(1), N1–C1–C2–B1 125.4(2).

The reaction of the enamine isomer mixture **10** with Piers' borane [HB(C_6_F_5_)_2_]^17^ was carried out analogously (*n*-pentane, r.t., 30 min) and gave the zwitterionic addition product **12a** as a white solid, isolated in 87% yield. Compound **12a** was characterized by an X-ray crystal structure analysis (suitable single crystals were obtained at –35 °C from a dichloromethane solution covered with a *n*-pentane layer). In the structure (see Fig. 1) the bulky tetramethylpiperidide unit is part of the iminium functional group. It has the remaining saturated C_2_-unit attached, which features the –B(H)(C_6_F_5_)_2_ substituent bonded to carbon atom C2. The boron atom shows a pseudo-tetrahedral coordination geometry with a sum of heavy atom bond angles of ∑B1^CCC^ = 335.6°. The structure of the B(C_6_F_5_)_3_/enamine adduct **11** is very similar (see the ESI†).

In solution (CD_2_Cl_2_) compound **12a** shows the ^1^H/^13^C NMR resonances of the [N]=CH– iminium functionality at *δ* 8.12 (d, ^3^ *J*_HH_ = 13.1 Hz) and *δ* 189.2 ppm, respectively. The ^11^B NMR signal occurs at *δ* –20.0 ppm as a doublet (^1^ *J*_BH_ ∼ 93 Hz). The corresponding ^1^H NMR [B]–H resonance was located at 2.74 ppm as a broad 1 : 1 : 1 : 1 intensity quartet. Due to the chiral center (C2) we observe a 1 : 1 pair of *o*,*p*,*m*-^19^F NMR signals and a total of four tetramethylpiperidino ^1^H NMR methyl group signals.

We reacted the enamine/HB(C_6_F_5_)_2_ adduct **12a** with carbon monoxide (CH_2_Cl_2_, r.t., 2.0 bar). The reaction was followed *in situ* by NMR spectroscopy. This showed the formation of the new product **13a** (which contained two B(C_6_F_5_)_2_ units) and the boron free enamine **10**. After 3 h reaction time workup eventually furnished the product **13a**, isolated as a white solid in 43% yield. Since we needed two molar equiv. of HB(C_6_F_5_)_2_ for a complete conversion of **12a** to the carbonylation product **13a**, we reacted the precursor **12a** with CO under similar conditions in the presence of an additional molar equivalent of Piers' borane. Workup after 3 h reaction time involving washing with CH_2_Cl_2_ gave the product **13a** in 41% yield (for details see the ESI†).

**Fig. 2:**
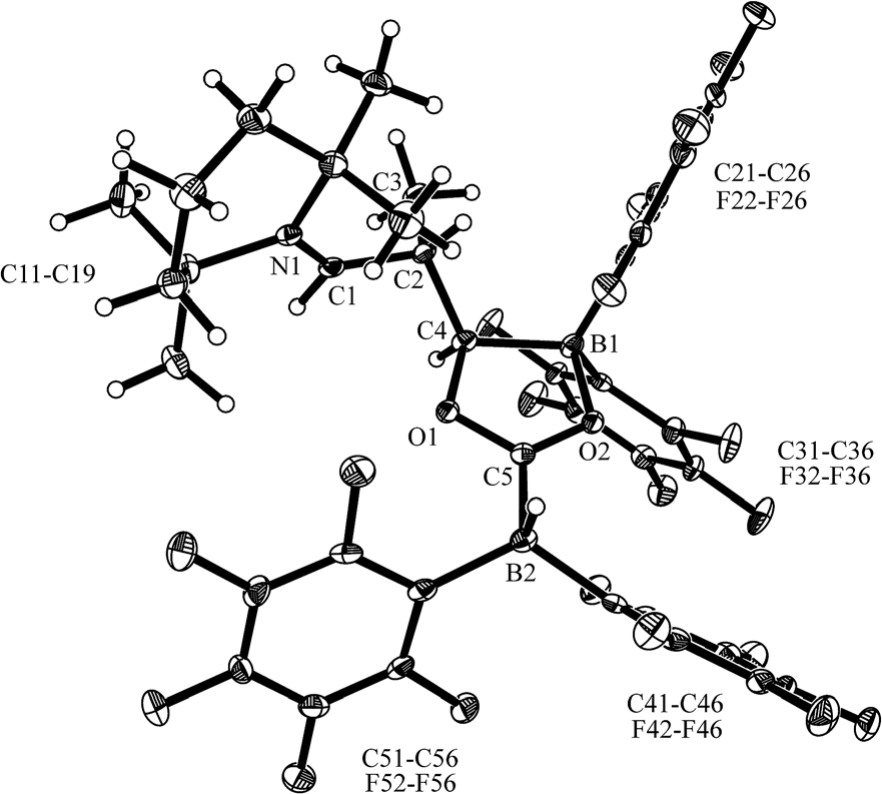
A projection of the carbonylation product **13a** (thermal ellipsoids are shown at the 50% probability level). Selected bond lengths (Å) and angles (°): N1–C1 1.286(3), C1–C2 1.498(3), C4–B1 1.658(3), B1–O2 1.551(3), O2–C5 1.272(2), B2–C5 1.604(3), C5–O1 1.320(2), O1–C4 1.494(3); C4–O1–C5 111.5(2), B1–O2–C5 113.4(2).

The X-ray crystal structure analysis of **13a** (Fig. 2, single crystals were obtained at –35 °C from a THF solution covered with a layer of *n*-pentane) revealed that a central five-membered heterocycle had been formed by head to tail coupling of two CO-molecules.^8^ The carbon atom of one of them (C4) now bears a hydrogen originating from a HB(C_6_F_5_)_2_ equivalent and the enamine derived substituent. The other CO equivalent is found C–O bonded between a pair of boron atoms. Both the boron atoms feature pseudotetrahedral coordination geometries (∑B1^CCC^ = 344.4°, ∑B2^CCC^ = 337.5°). The enamine derived residue shows an iminium functionality.

In solution (THF-d_8_) compound **13a** features ^1^H/^13^C NMR resonances of the iminium moiety at *δ* 8.68 (^3^ *J*_HH_ = 10.4 Hz) and 184.5 ppm ([N]=CH–), respectively. The central CO derived –O–C[B]–O– carbon atom shows a ^13^C NMR signal at *δ* 213.7 ppm (C5), whereas the [B]CH carbon atom (C4 in Fig. 2) shows a broad ^13^C NMR resonance *δ* 86.2 ppm.

Compound **13a** shows a pair of ^11^B NMR signals at *δ* 2.6 and –26.1 ppm (^1^ *J*_BH_ = 85.8 Hz). Due to the C2–chirality center both the B(C_6_F_5_)_2_ units show the ^19^F NMR signals of pairs of diastereotopic C_6_F_5_ substituents.

**Scheme 3:**
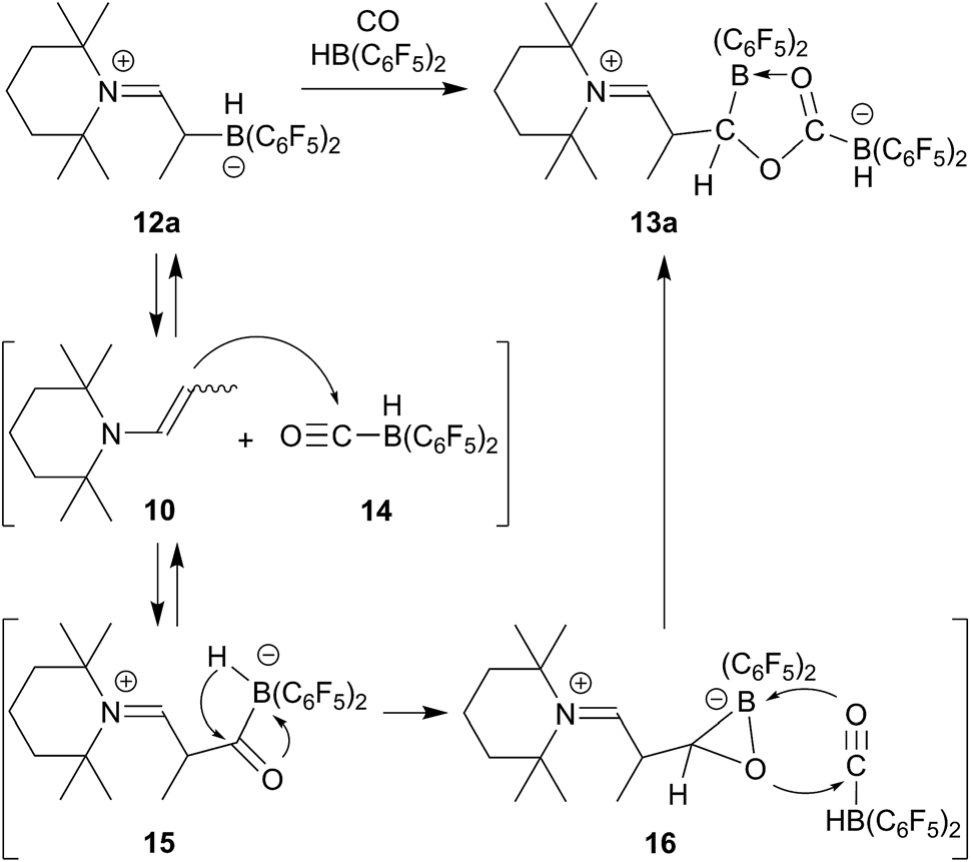


We assume that the enamine/HB(C_6_F_5_)_2_ C/B-adduct becomes reversible under the applied carbonylation conditions and we, consequently, observe a reaction under frustrated Lewis pair (FLP) conditions.^18,20,23,24^ CO activation can occur by formation of Piers' borane carbonyl^25,26^ [OC–B(H)(C_6_F_5_)_2_] **14** as we had recently shown (we had actually isolated **14** at low temperature and characterized this borane carbonyl by X-ray diffraction). FLP addition of the enamine nucleophile followed by hydridoborate reduction of the acyl group in **15** would then lead to the intermediate **16** which could add to a second equivalent of the borane carbonyl to eventually yield the observed CO coupling product **13a** (see Scheme 3).

**Scheme 4:**
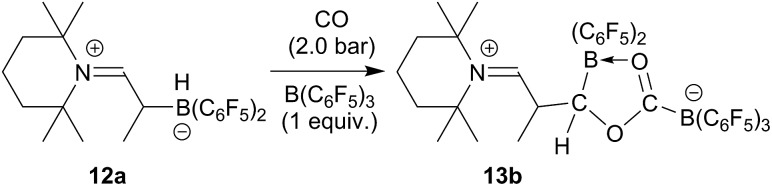


The reaction of the enamine/HB(C_6_F_5_)_2_ FLP **12a** with CO proceeds similarly in the presence of one molar equiv. of the strong boron Lewis acid B(C_6_F_5_)_3_. The reaction was carried out at r.t. in dichloromethane with 2.0 bar carbon monoxide pressure. Workup after 3 h reaction time eventually gave the CO coupling product **13b** as a white solid, isolated in 76% yield. In THF-d_8_ solution it shows a pair of ^11^B NMR signals at *δ* 2.6 and *δ* –17.7 ppm. We monitor a single set of *o*,*p*,*m*-C_6_F_5_^19^F NMR resonances of the exocyclic B(C_6_F_5_)_3_ substituent and the corresponding resonances of a pair of diastereotopic C_6_F_5_ groups of the endocyclic B(C_6_F_5_)_2_ moiety. Compound **13b** was also characterized by X-ray diffraction. Its structure is analogous to that of **13a** (for details see the ESI†). We assume a similar reaction scheme for the formation of **13b** as we had discussed it for **13a** (see above) (Scheme 4).

### NO/NO coupling with the enamine/HB(C_6_F_5_)_2_ C/B Lewis pair

**Scheme 5:**
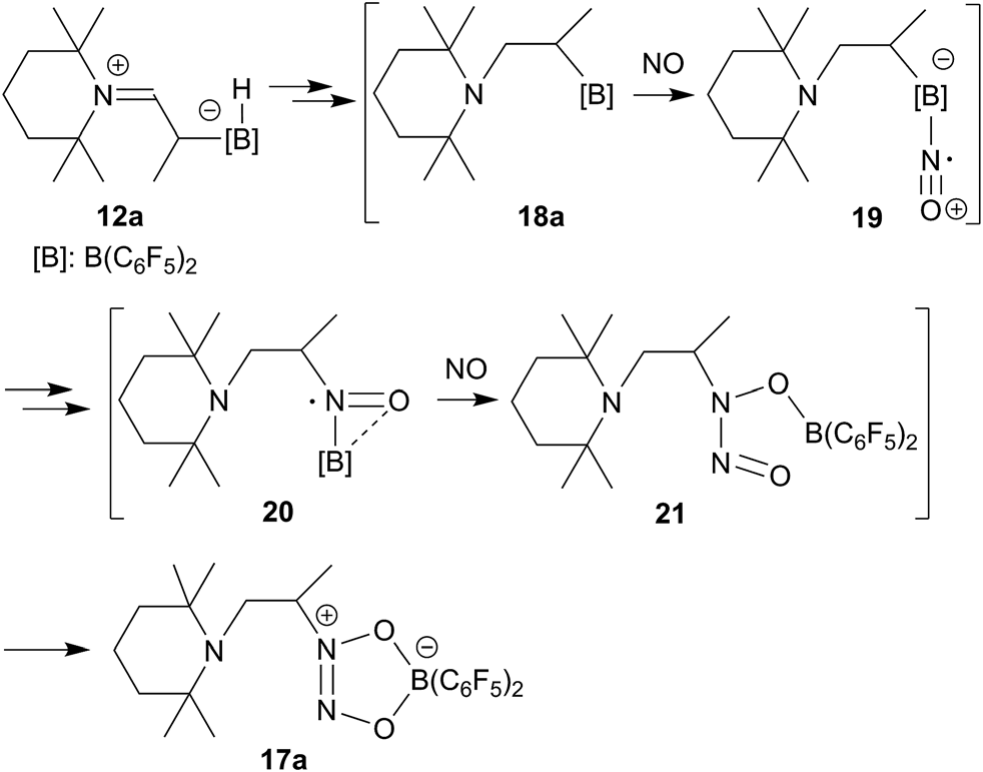


**Fig. 3:**
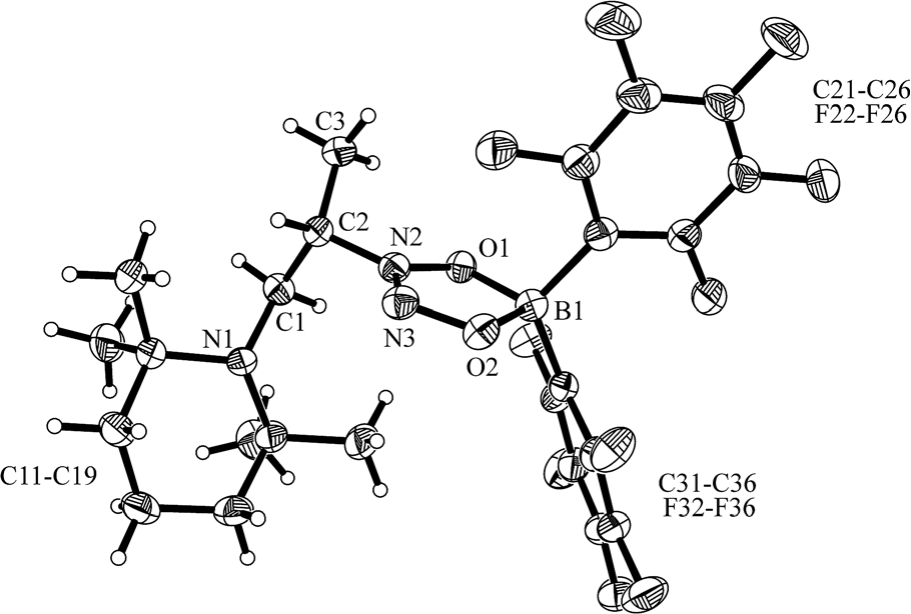
Molecular structure of the NO coupling product **17a** (thermal ellipsoids are shown at the 30% probability level). Selected bond lengths (Å) and angles (°): N1–C1 1.459(3), N2–C2 1.473(3), N2–N3 1.256(3), N2–O1 1.344(2), N3–O2 1.333(3), O1–B1 1.527(3), O2–B1 1.538(3); N1–C1–C2 114.5(2), C2–N2–O1 119.2(2), O1–N2–N3 117.3(2), N2–O1–B1 105.6(2), N3–O2–B1 110.3(2), O1–B1–O2 97.6(2).

The enamine/HB(C_6_F_5_)_2_ adduct **12a** reacted equally well with nitric oxide (NO). A solution of **12a** in dichloromethane was exposed to a NO atmosphere (1.0 bar) for 3 h. Workup by removing the solvent *in vacuo* and washing of the residue with *n*-pentane gave the NO coupling product **17a** as a pale yellow solid. It was isolated in 76% yield (see Scheme 5). Single crystals of compound **17a** suited for the X-ray crystal structure analysis were obtained from a *n*-pentane solution at –35 °C (see Fig. 3). It shows that two NO molecules had been head to head coupled. This has formed the observed central planar five-membered N_2_O_2_B containing heterocycle. It contains a short N2=N3 linkage and pairs of N–O and B–O bonds in the σ-bond length range. Nitrogen atom N2 has the amino-alkyl substituent attached to it. We note that this does not contain the iminium moiety of the starting material (**12a**) any more but instead features a saturated *tert* amine [N]–CH_2_– unit.

In solution compound **17a** shows the ^1^H NMR signals of the [N]–CH_2_–CH– section of the substituent at *δ* 2.72, 3.05 (CH_2_, ^2^ *J*_HH_ = 16.4 Hz, ^3^ *J*_HH_ = 9.5 Hz, ^13^C: *δ* 47.9) and *δ* 4.80 ppm (CH, ^13^C: *δ* 69.3), respectively. It features a ^11^B NMR resonance at *δ* +12.2 ppm and two sets of *o*,*p*,*m*-^19^F NMR signals of the pair of diastereotopic C_6_F_5_ substituents at boron.

From the structure of compound **17a** (see Fig. 3), which contains the saturated [N]–CH_2_–CH– moiety, we must assume that the NO reaction started from the N/B FLP isomer **18a** (see Scheme 5 and below).^20,23,24^ NO addition to boron would give the radical **19** which might have rearranged to its isomer **20**.^20,27^ Trapping with a second equivalent of NO would then straightforwardly lead to **17a**. We must stress that this pathway which is depicted in Scheme 5 provides a mere possible rationalization for the observed formation of the NO/NO coupling product; so far none of the alleged intermediates has been observed directly.

**Scheme 6:**
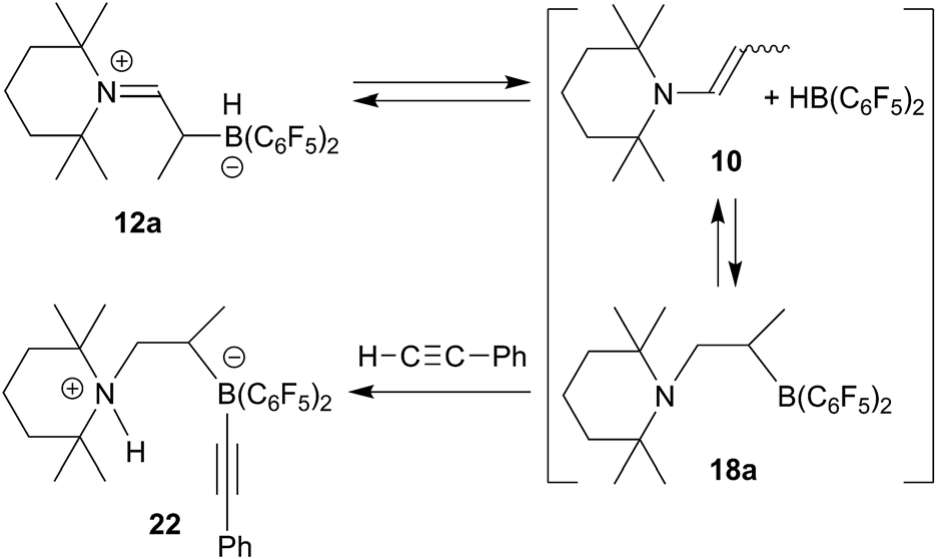


We have, however, obtained additional indirect evidence for the formation of the N/B FLP **18a**, which is apparently in an endothermal equilibrium situation with **12a** (a situation that had previously been observed with other enamine/RB(C_6_F_5_)_2_ borane addition products as well).^18^ In this case we added a slight excess of phenylacetylene to a solution of the enamine/HB(C_6_F_5_)_2_ adduct **12a** in dichloromethane solution. The mixture was kept at r.t. for 1 h. Workup then gave the reaction product **22**, which we isolated in 91% yield (see Scheme 6).

**Fig. 4:**
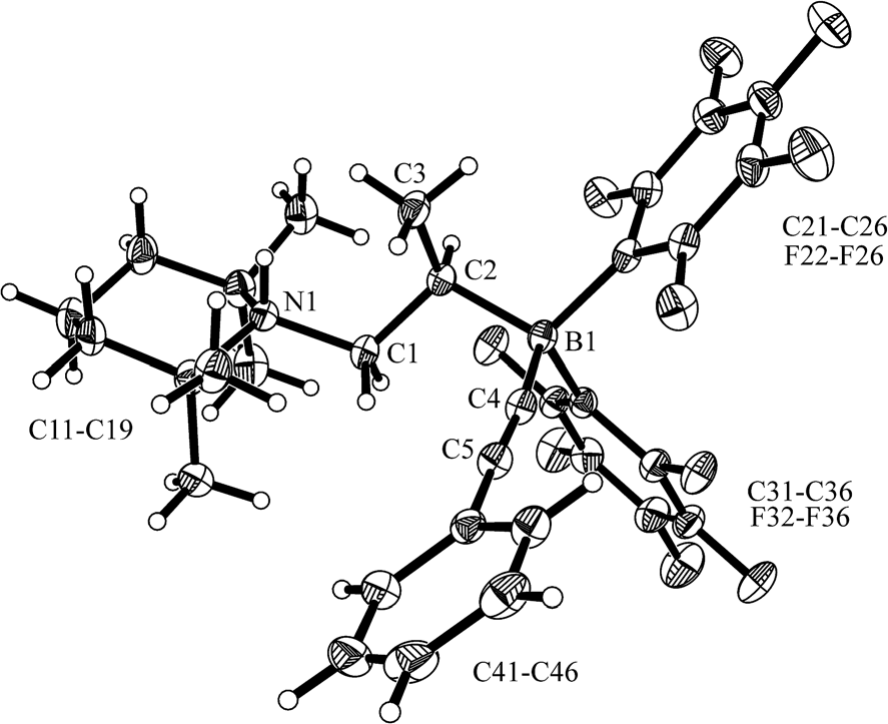
A projection of the molecular structure of compound **22** (thermal ellipsoids are shown at the 30% probability level). Selected bond lengths (Å) and angles (°): B1–C4 1.593(3), C4–C5 1.206(3), C5–C41 1.439(3), B1–C2 1.677(3), C1–C2 1.532(3), C1–N1 1.541(3); B1–C4–C5 174.3(2), C4–C5–C41 175.7(2), N1–C1–C2–B1 167.9(2), ∑N1^CCC^ 343.5.

The X-ray crystal structure analysis of **22** (see Fig. 4) revealed that we had apparently trapped the typical N/B FLP reaction product with the terminal acetylene.^28^ The alkyne had become deprotonated to give the ammonium cation section of **22** and the resulting alkynyl carbanion had become attached to the boron atom. In the zwitterionic compound a saturated –CH_2_–CH(CH_3_)– bridge is formed connecting the boron and nitrogen atoms. Consequently, we observe the ^13^C NMR acetylide signals at *δ* 111.9 and 98.4 ppm, respectively (^11^B: *δ* –17.3 ppm) and a broad NH ^1^H NMR resonance at *δ* 6.08 ppm. We assume that the adduct formation of the enamine **10** with Piers' borane to give **12a** is reversible in solution. From this equilibrium there might be an (endothermic) pathway of hydroboration of the enamine double bond to generate the N/B FLP **18a** *in situ* in an equilibrium situation. This we have not observed as such,^18^ but we assume that its typical N/B FLP reaction with the added phenylacetylene trapping reagent eventually led to the formation of the observed product **22** (see Scheme 6 and Fig. 4).

**Scheme 7:**
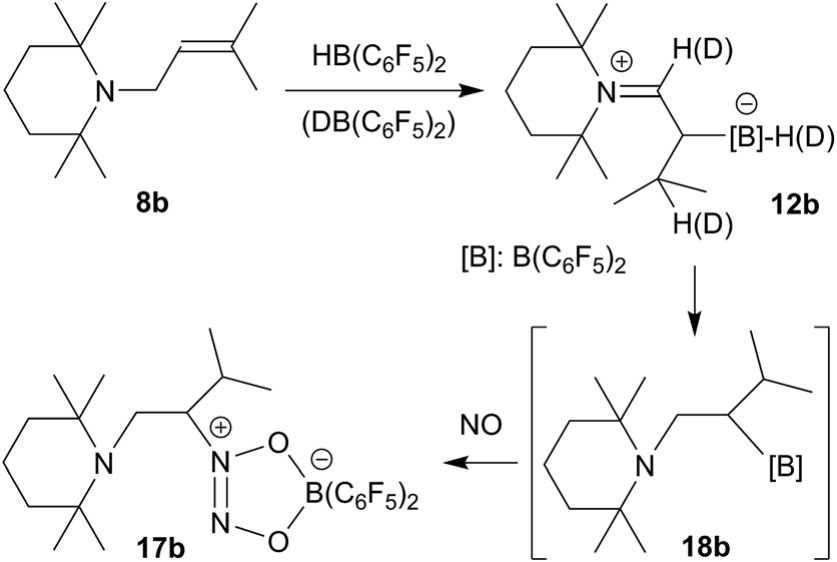


This set the scene for the formation of the isopropyl substituted enamine/HB(C_6_F_5_)_2_ adduct **12b** and its reaction with nitric oxide. We treated the substituted allyl amine precursor **8b** ^20,29^ with one molar equiv. of HB(C_6_F_5_)_2_ in *n*-pentane. This gave a yellow solution within 10 min. Workup after 30 min reaction time eventually gave the zwitterionic iminium/hydridoborate product **12b**, which we isolated in 89% yield (see Scheme 7). The NMR spectra showed the presence of the iminium ion moiety (^13^C: *δ* 187.1 ppm), and a ^11^B NMR resonance at *δ* –21.1 ppm (d, ^1^ *J*_BH_ ∼ 92 Hz). We observed the ^1^H NMR signals of the [N]=CH– proton at *δ* 8.40 ppm, the –CHMe_2_ isopropyl–H at *δ* 2.06 and the [B]H at ∼2.84 ppm (broad 1 : 1 : 1 : 1 quartet).

We also did the analogous reaction of **8b** with the isotopically labelled DB(C_6_F_5_)_2_ reagent. In the product we now find the deuterium atom scrambled over these two CH and the BH position listed above, which indicates that in this case the isomerization reaction of **8b** to the respective enamine is probably taking place by means of hydroboration/retro-hydroboration (see Scheme 7, for further details see the ESI†).

**Fig. 5:**
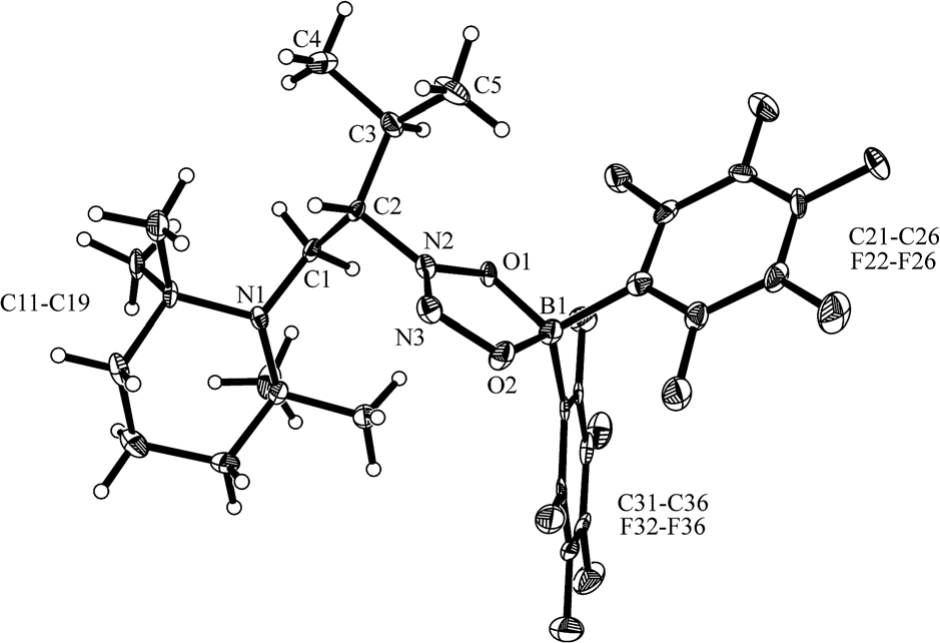
A view of the molecular structure of the NO coupling product **17b** (thermal ellipsoids are shown at the 50% probability level). Selected bond lengths (Å) and angles (°): N1–C1 1.455(5), N2–C2 1.482(5), N2–N3 1.252(5), N2–O1 1.334(4), N3–O2 1.323(5), O1–B1 1.508(6), O2–B1 1.536(6); N1–C1–C2 113.5(4), C2–N2–O1 118.8(3), O1–N2–N3 117.4(4), N2–O1–B1 105.4(3), N3–O2–B1 109.4(3), O1–B1–O2 98.3(3).

Compound **12b** reacted rapidly with nitric oxide. A solution of **12b** in dichloromethane was stirred for 3 h at r.t. in a NO atmosphere (1.0 bar). Workup involving extraction with *n*-pentane eventually gave the NO coupling product **17b**, which we isolated as a pale yellow solid in 69% yield (see Scheme 7). It was characterized by X-ray diffraction (see Fig. 5) and by NMR spectroscopy. The ^1^H NMR spectrum shows a total of six methyl group signals and an ABX type pattern of the [N]–CH_2_–CH– moiety [CH_2_: *δ* 2.95, 3.08 (^2^ *J*_HH_ = 16.3 Hz, ^3^ *J*_HH_ = 9.8 Hz), 4.44 (CH); ^13^C: *δ* 44.2, 78.9 ppm]. The ^11^B NMR resonance of compound **17b** occurs at *δ* +12.6 ppm and we have observed two sets of *o*,*p*,*m*-^19^F NMR signals of the pair of diastereotopic C_6_F_5_ substituents at boron.

## Conclusions

The HB(C_6_F_5_)_2_ adduct of the very bulky enamine **8a** reacts very differently with the element oxides CO and NO.^30^ Its facile reaction with carbon monoxide results in a selective head to tail coupling of the CO molecule. Formally, the initial sequence might well be regarded as an addition reaction of a C/B frustrated Lewis pair^31^ to carbon monoxide. We assume a reaction pathway initiated by reversible HB(C_6_F_5_)_2_ cleavage from the adduct. It may then combine *via* nucleophilic enamine addition to Piers' borane carbonyl [(C_6_F_5_)_2_B(H)–CO **14**], a reactive borane carbonyl that we had recently prepared and characterized. Hydridoborate carbonyl reduction and B/O addition to a second borane Lewis acid CO equivalent might then close the reaction cycle as it was shown in Scheme 3. It seems an essential consequence of this reaction that the enamine/HB(C_6_F_5_)_2_ adduct formation is reversible and that, consequently, the **10**/HB(C_6_F_5_)_2_ ⇄ **12a** system may function as a reactive C/B frustrated Lewis pair.

**Scheme 8:**
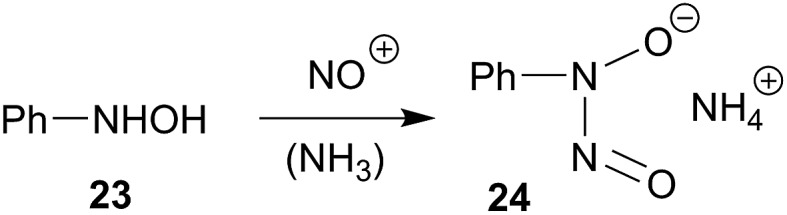


The reaction of **12a** with NO revealed another reaction type of the HB(C_6_F_5_)_2_/enamine C/B Lewis pair adduct. We here observe the formation of the product of selective NO/NO coupling. This is a typical reaction mode observed for many metal alkyls.^10–15^ Here we have probably found the analogous reaction of an alkyl–B(C_6_F_5_)_2_ functional group. Therefore, we assume that this reaction is initiated by the reaction of NO with the –CHR–B(C_6_F_5_)_2_ function of the *in situ* generated vicinal N/B Lewis pair, which may be formed by cleavage of the enamine/HB(C_6_F_5_)_2_ Lewis adduct followed by anti-Markovnikov hydroboration. The reaction then seems to follow the usual pathway, as it is often observed in the selective NO/NO coupling of metal hydrocarbyls,^15^ here to eventually give the respective boron based-ON(R)NO products **17**. The NO coupling products^16^ **17** are topologically related to cupferron (**24**), a reagent that had frequently be used for chelate metal complexation.^32–34^ It is formed by nitrosation of *N*-phenyl-hydroxylamine (**23**) (Scheme 8). We will find out whether the boron based *N*-nitrosohydroxylaminato groups could potentially be used directly as reagents for metal complex formation.

The dual reaction pathway of the enamine/HB(C_6_F_5_)_2_ Lewis pair **12** with CO and with NO indicates a remarkable variability of bulky Lewis acid/Lewis base combinations in small molecule chemistry.
