# Supplementary material for: CO/CO and NO/NO coupling at a hidden frustrated Lewis pair template
Source: Chem Sci. 2017 Jan 4;8(3):2457–63. doi: 10.1039/c6sc04459j (PMC5369402; doi:10.1039/c6sc04459j)
Supplement: Supplementary file 1 [file SC-008-C6SC04459J-s001.pdf]

Electronic Supplementary Material (ESI) for Chemical Science.
This journal is © The Royal Society of Chemistry 2017

Supporting Information

CO/CO and NO/NO coupling at a hidden frustrated Lewis pair template

Tongdao Wang,[a] Long Wang,[a] Constantin G. Daniliuc,[a] Kamil Samigullin,[b] Matthias Wagner,[b] Gerald Kehr[a] and Gerhard Erker*[a]

[a] Organisch-Chemisches Institut, Westfälische Wilhelms-Universität Münster, Corrensstraße 40, 48149 Münster, Germany

[b] Institut für Anorganische und Analytische Chemie Goethe-Universität Frankfurt, Max-von-Laue-Straße 7, 60438 Frankfurt (Main) (Germany)

* erker@uni-muenster.de

General Information: All syntheses involving air- and moisture sensitive compounds were carried out using standard Schlenk-type glassware (or in a glove box) under an atmosphere of argon. Solvents were stored under an argon atmosphere. NMR spectra were recorded on a *Varian* Inova 500 (^{1}H: 500 MHz, ^{13}C: 126 MHz, ^{19}F: 470 MHz, ^{11}B:160 MHz) or a *Varian* Inova 600 (^{1}H: 600 MHz, ^{13}C: 151 MHz, ^{19}F: 564 MHz, ^{11}B:192 MHz). ^{1}H NMR and ^{13}C NMR: chemical shifts δ are given relative to TMS and referenced to the solvent signal. ^{19}F NMR: chemical shifts δ are given relative to $CFCl_3$ (external reference), ^{11}B NMR: chemical shifts δ are given relative to $BF_3{\cdot}Et_2O$ (external reference). NMR assignments were supported by additional 2D NMR experiments. Melting points and decomposition points were obtained with a DSC 2010 (*TA Instruments*). X-Ray diffraction: For compounds **13b**, **17a** and **22** data sets were collected with a Nonius Kappa CCD diffractometer. Programs used: data collection, COLLECT (R. W. W. Hooft, Bruker AXS, 2008, Delft, The Netherlands); data reduction Denzo-SMN (Z. Otwinowski, W. Minor, *Methods Enzymol.* **1997**, *276*, 307-326); absorption correction, Denzo (Z. Otwinowski, D. Borek, W. Majewski, W. Minor, *Acta Crystallogr.* **2003**, *A59*, 228-234); structure solution SHELXS-97 (G. M. Sheldrick, *Acta Crystallogr.* **1990**, *A46*, 467-473); structure refinement SHELXL-97 (G. M. Sheldrick, *Acta Crystallogr.* **2008**, *A64*, 112-122) and graphics, XP (BrukerAXS, 2000). For

compounds **11**, **12a**, **13a**, **17b** and **26** data sets were collected with a D8 Venture Dual Source 100 CMOS diffractometer. Programs used: data collection: APEX2 V2014.5-0 (Bruker AXS Inc., 2014); cell refinement: SAINT V8.34A (Bruker AXS Inc., 2013); data reduction: SAINT V8.34A (Bruker AXS Inc., 2013); absorption correction, SADABS V2014/2 (Bruker AXS Inc., 2014); structure solution SHELXT-2014 (Sheldrick, 2014); structure refinement SHELXL-2014 (Sheldrick, 2014) and graphics, XP (Bruker AXS Inc., 2014). *R*-values are given for observed reflections, and wR^2 values are given for all reflections. *Exceptions and special features*: For compound **26** one C_6F_5 group and one CF_3 group were found disordered over two positions. Compound **13b** presents the iminium moiety disordered over two positions. Several restraints (SADI, SAME, ISOR and SIMU) were used in order to improve refinement stability. For compound **26** one and a half badly disordered dichloromethane molecules, for compound **13b** one THF molecule and for compound **22** one dichloromethane molecule were found in the asymmetrical unit. The program SQUEEZE (A. L. Spek J. Appl. Cryst., 2003, 36, 7-13) was therefore used to remove mathematically the effect of the solvent. The quoted formula and derived parameters are not included the squeezed solvent molecules. In compounds **12a** and **13a** the hydrogen positions at boron atom were refined freely.

Materials: Compounds **8a**,[1] **26**,[2] $HB(C_6F_5)_2$,[3] and $DB(C_6F_5)_2$[4] were prepared according to the literature procedure. [(1) (a) T. Wang, G. Kehr, L. Liu, S. Grimme, C. D. Daniliuc and G. Erker, *J. Am. Chem. Soc.,* 2016, **138**, 4302; (b) V. Sumerin, F. Schulz, M. Atsumi, C. Wang, M. Nieger, M. Leskelä, T. Repo, P. Pyykkö and B. Rieger, *J. Am. Chem. Soc.,* 2008, **130**, 14117. (2) L. Wang, K. Samigullin, M. Wagner, A. C. McQuilken, T. H. Warren, C. G. Daniliuc, G. Kehr and G. Erker, *Chem. -Eur. J.,* 2016, **22**, 11015. (3) (a) D. J. Parks, R. E. von H. Spence and W. E. Piers, *Angew. Chem., Int. Ed. Engl.*, 1995, **34**, 809; (b) D. J. Parks, W. E. Piers, G. P. A. Yap, *Organometallics,* 1998, **17**, 5492. (4) B.-H. Xu, G. Kehr, R. Fröhlich, S. Grimme, G. Erker, *J. Am. Chem. Soc.,* 2011, **133**, 3480.]

Synthesis of compound trans/cis-10

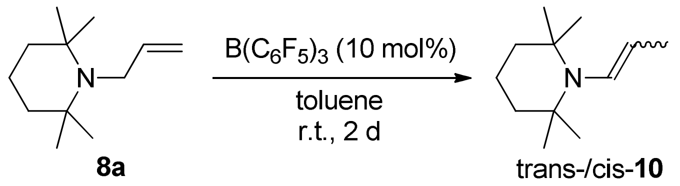

A solution of 1-allyl-2,2,6,6-tetramethylpiperidine (**8a**) (2.8665 g, 15.8 mmol) in toluene (5 mL) was added to a suspension of $B(C_6F_5)_3$ (809.5 mg, 1.58 mmol) in toluene (30 mL), which initially resulted in a yellow solution, then after 20 min. a brown suspension. The mixture was stirred at room temperature for about 2 days to give a dark brown solution,

which was distilled to give the colorless solution of compound **10** in toluene (bp. 80 °C, 3×10^{-1} mbar). The solvent was removed from the obtained solution in vacuo at 0 °C to give a oil. Yield: 2.0639 g, 11.4 mmol, 72%.

Anal. Calcd. calc. for $C_{12}H_{23}N$: C, 79.49; H, 12.79; N, 7.72. Found: C, 79.28; H, 12.80; N, 7.83.

A solution of the obtained oil in C_6D_6 showed a mixture of trans-/cis-**10** [1:0.8 (1H)]

NMR data for compound *trans*-10 (major isomer assigned from the mixture): [TMP: 2,2,6,6-tetramethylpiperidino]

^{1}H NMR (500 MHz, 299 K, C_6D_6): δ = 5.75 (dq, $^3J_{HH}$ = 13.4 Hz, $^4J_{HH}$ = 1.6 Hz, 1H, NCH=), 5.30 (dq, $^3J_{HH}$ = 13.4 Hz, $^3J_{HH}$ = 6.6 Hz, 1H, =CH), 1.62 (dd, $^3J_{HH}$ = 6.6 Hz, $^4J_{HH}$ = 1.6 Hz, 3H, CH_3), 1.46 (m, 2H, CH_2^{TMP}), 1.43 (m, 4H, $^CCH_2^{TMP}$), 1.09 (s, 12H, CH_3^{TMP}).

^{13}C{^{1}H} NMR (126 MHz, 299 K, C_6D_6): δ = 132.6 (NCH=), 121.0 (=CH), 53.7 (NC^{TMP}), 41.5 ($^CCH_2^{TMP}$), 27.9 (CH_3^{TMP}), 18.1 (CH_2^{TMP}), 15.5 (CH_3).

NMR data for compound *cis*-10 (minor isomer assigned from the mixture): [TMP: 2,2,6,6-tetramethylpiperidino]

^{1}H NMR (500 MHz, 299 K, C_6D_6): δ = 5.68 (dq, $^3J_{HH}$ = 7.8 Hz, $^4J_{HH}$ = 1.8 Hz, 1H, NCH=), 5.40 (dq, $^3J_{HH}$ = 7.8 Hz, $^3J_{HH}$ = 6.6 Hz, 1H, =CH), 1.69 (dd, $^3J_{HH}$ = 6.6 Hz, $^4J_{HH}$ = 1.8 Hz, 3H, CH_3), 1.47 (m, 2H, CH_2^{TMP}),

1.46 (m, 4H, $^{C}CH_2^{TMP}$), 1.01 (s, 12H, CH_3^{TMP}).

^{13}C{^{1}H} NMR (126 MHz, 299 K, C_6D_6): δ = 132.3 (NCH=), 127.2 (=CH), 54.2 (NC^{TMP}), 41.0 ($^{C}CH_2^{TMP}$), 26.8 (br, CH_3^{TMP}), 18.2 (CH_2^{TMP}), 13.2 (CH_3).

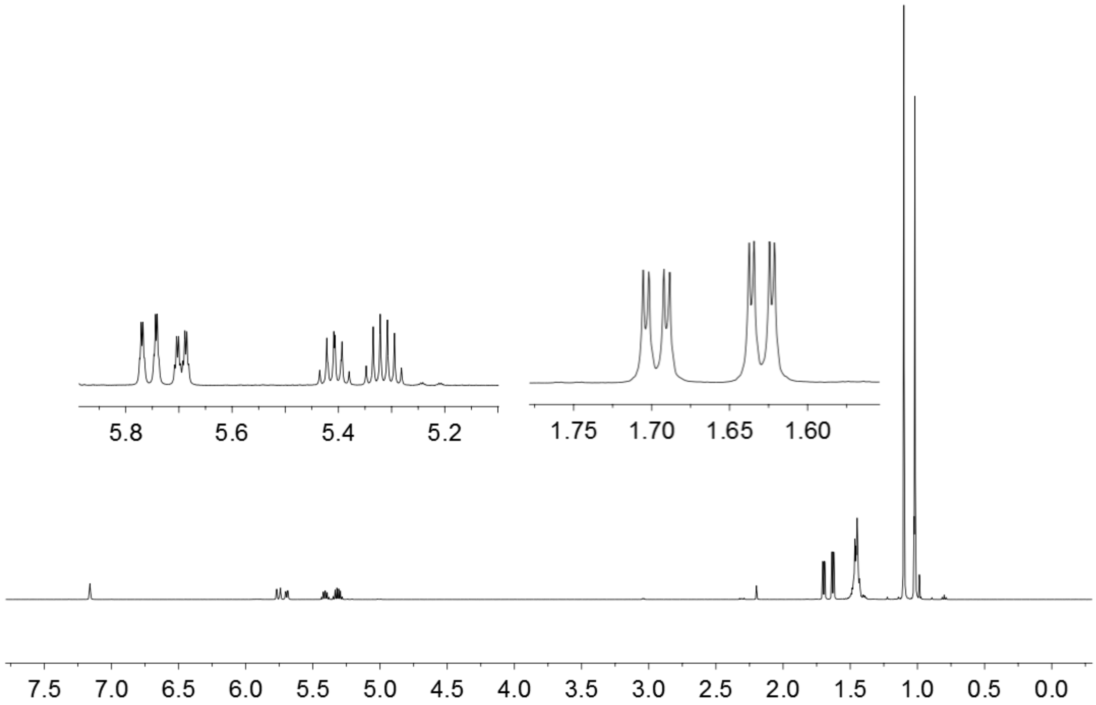

Figure S1. ^{1}H NMR (500 MHz, 299 K, C_6D_6) spectrum of compound **10**.

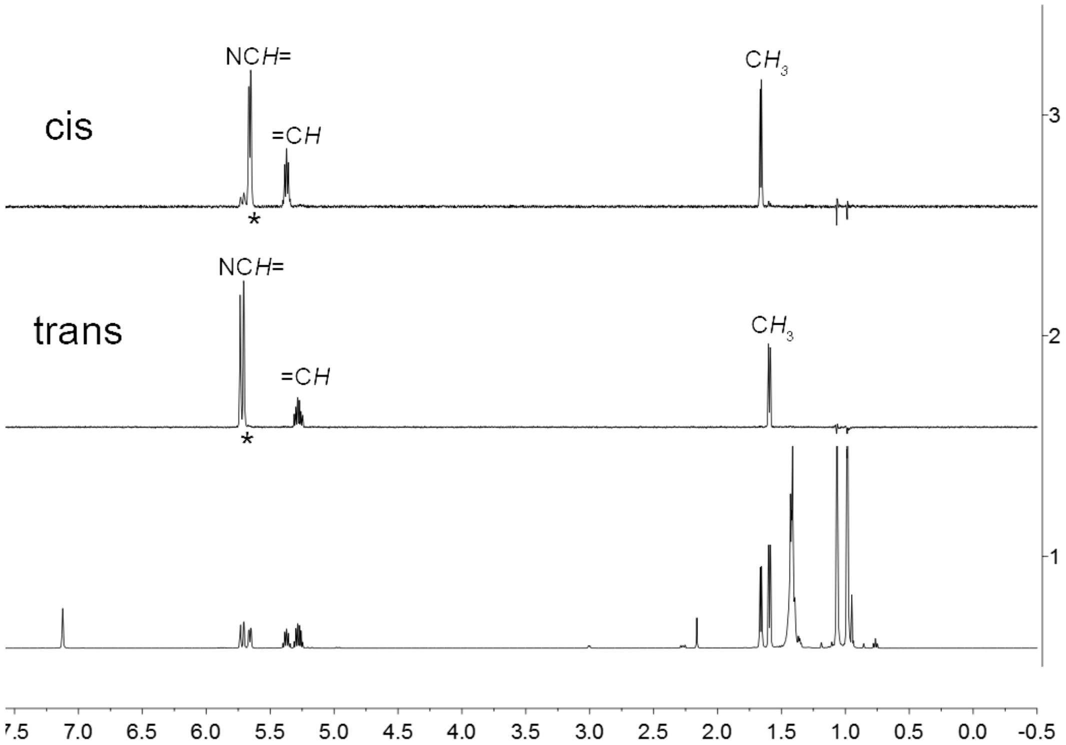

Figure S2. (1) ^{1}H NMR and (2,3) ^{1}H{^{1}H} TOCSY (500 MHz, 299 K, C_6D_6) spectra of compound **10**. * Irradiation points: (2) $^{1}H_{irr}$ = 5.76 (NCH=^{trans}), (3) 5.69 (NCH=^{cis}).

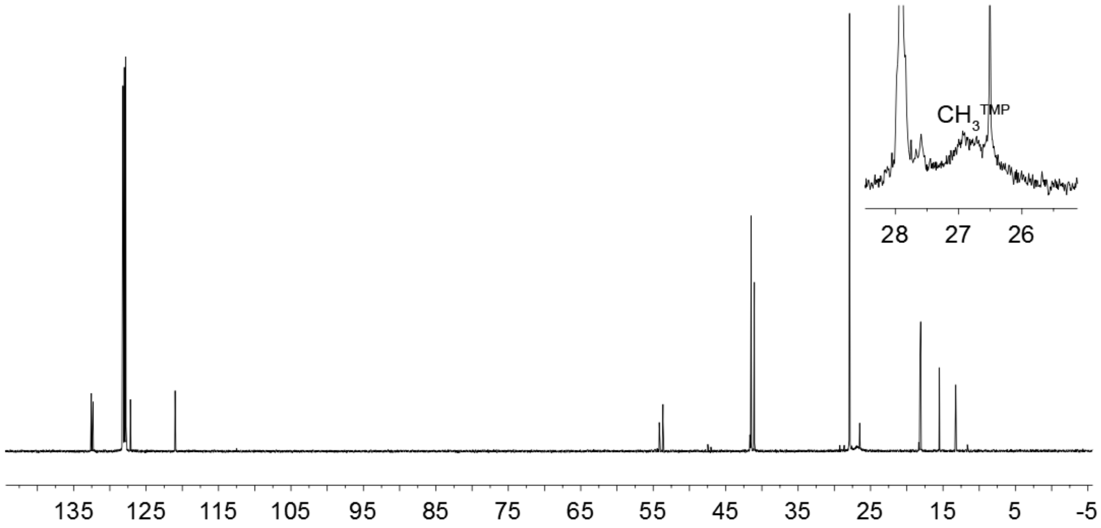

Figure S3. ^{13}C{^{1}H} NMR (126 MHz, 299 K, C_6D_6) spectrum of compound **10**.

Synthesis of compound 11

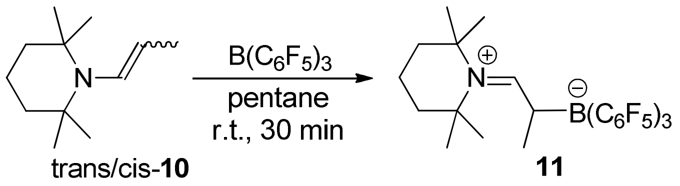

Method A (*starting from trans-/cis-**10***): A solution of trans-/cis-**10** (90.7 mg, 0.50 mmol) in *n*-pentane (2 mL) was added to a suspension of $B(C_6F_5)_3$ (255.9 mg, 0.50 mmol) in *n*-pentane (30 mL), which firstly resulted in a pale yellow solution immediately. The mixture was stirred at room temperature for 30 min to give a white precipitate. The product was collected by filtration and dried in vacuo to give a white solid. Yield: 322.3 mg, 0.46 mmol, 93%.

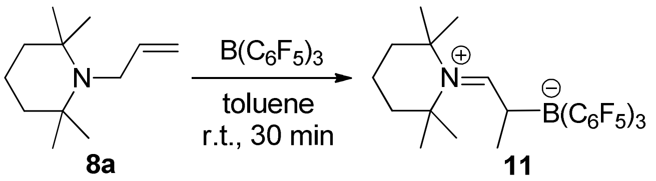

Method B (*starting from 1-allyl-2,2,6,6-tetramethylpiperidine **8a***): 1-Allyl-2,2,6,6-tetramethylpiperidine (**8a**) (181.3 mg, 1.00 mmol) was added to the suspension of $B(C_6F_5)_3$ (511.5 mg, 1.00 mmol) in toluene (20 mL). The mixture was stirred at room temperature for about 30 min to give a white precipitate. The product was collected by filtration, washed with toluene (3×3 mL) and dried in vacuo to give a white solid. Yield: 563.2 mg, 0.81 mmol, 81%.

Anal. Calcd. for $C_{30}H_{23}F_{15}NB$: C, 51.97; H, 3.34; N, 2.02. Found: C, 51.64; H, 3.21; N, 2.11.

Decomp. (DSC): 150°C

[Comment: a solution of the white solid in CD_2Cl_2 decomposed after a few hours at 299K]

[TMP: 2,2,6,6-tetramethylpiperidino]

^{1}H NMR (600 MHz, 238 K, CD_2Cl_2): δ = 8.74 (d, $^3J_{HH}$ = 12.6 Hz, 1H, N=CH), 3.21 (br, 1H, BCH), 2.07/1.78 (each 1H), 1.87 (2H) (each m, $^CCH_2^{TMP}$), 1.81 (m, 2H, CH_2^{TMP}), 1.54/1.52, 1.52/1.21 (each s, each 3H, CH_3^{TMP}), 1.01 (br m, 3H, CH_3).

^{13}C{^{1}H} NMR (151 MHz, 238 K, CD_2Cl_2): δ = 189.3 (br, N=CH), 147.7 (dm, $^1J_{FC}$ ~ 236 Hz, C_6F_5), 138.1 (dm, $^1J_{FC}$ ~ 247 Hz, C_6F_5), 136.5 (dm, $^1J_{FC}$ ~ 246 Hz, C_6F_5), 122.5 (i-C_6F_5), 69.1, 64.9 (NC^{TMP}), 36.5, 33.2 ($^CCH_2^{TMP}$), 34.8 (br, BCH), 32.0/28.5, 30.0/27.6 (CH_3^{TMP}), 15.3 (CH_3), 13.2 (CH_2^{TMP}).

^{11}B{^{1}H} NMR (192 MHz, 238 K, CD_2Cl_2): δ = -11.9 ($\nu_{1/2}$ ~ 15 Hz).

^{19}F NMR (564 MHz, 238 K, CD_2Cl_2): δ = -130.5 (br, 2F, o-C_6F_5), -160.7 (t, $^3J_{FF}$ = 20.9 Hz, 1F, p-C_6F_5), -165.2 (br, 2F, m-C_6F_5) [$\Delta\delta^{19}F_{m,p}$ = 4.5].

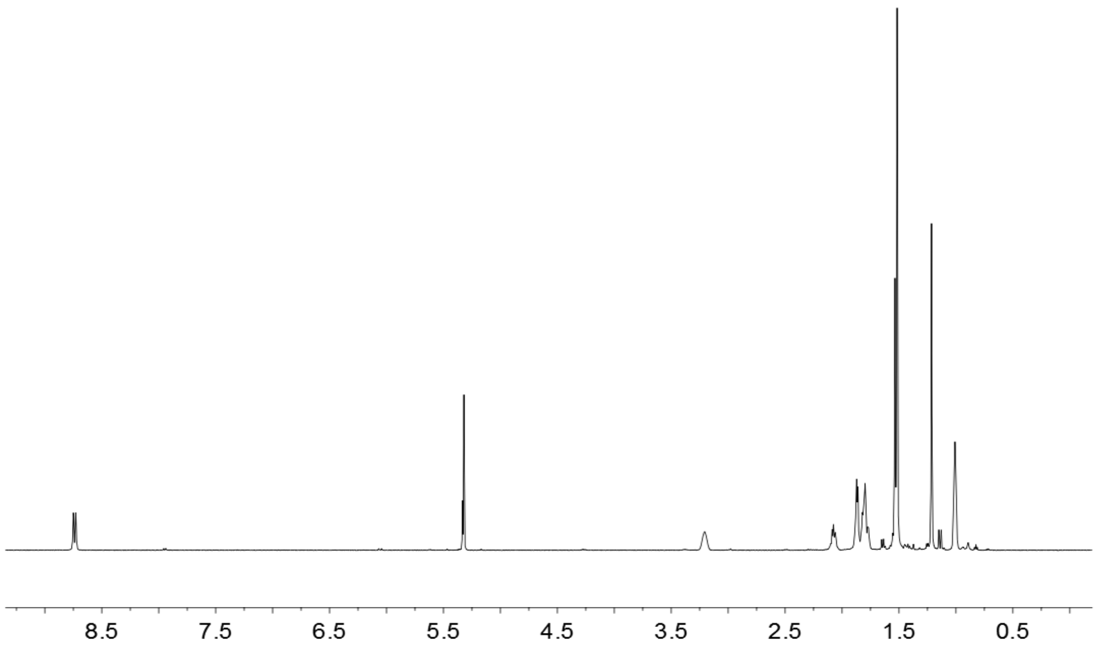

Figure S4. ^{1}H NMR (600 MHz, 238 K, CD_2Cl_2) spectrum of compound **11**.

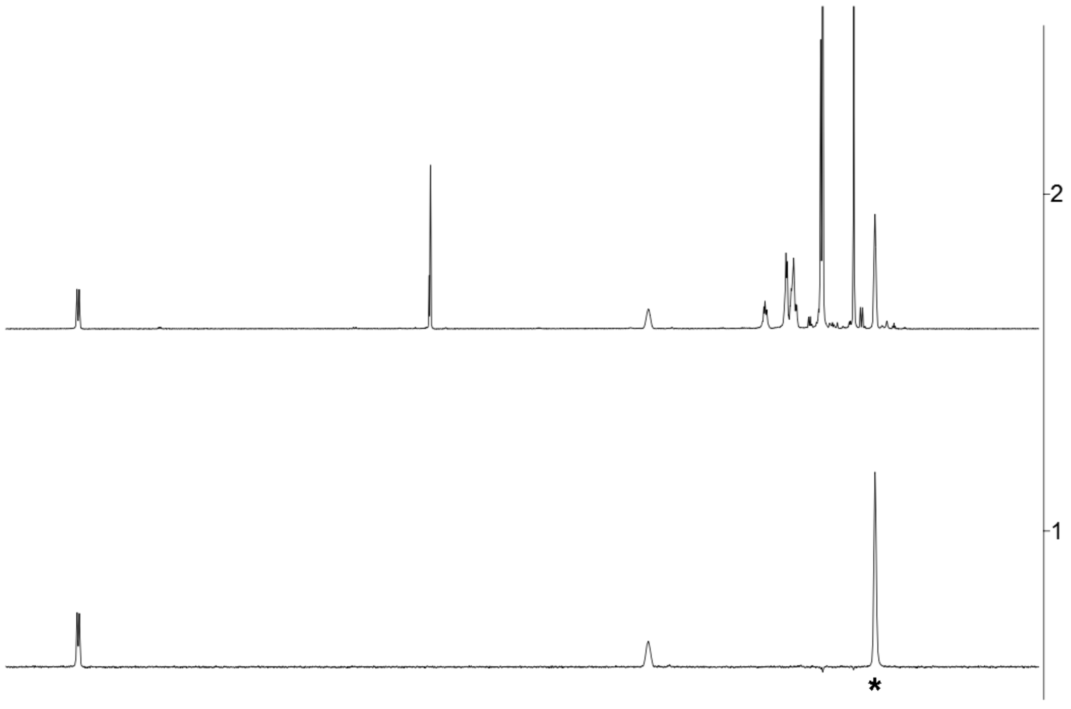

Figure S5. (2) ^{1}H NMR and (1) $^{1}H\{^{1}H\}$ TOCSY (600 MHz, 238 K, CD_2Cl_2) spectra of compound **11**. * Irradiation point: $^{1}H_{irr}$ = 1.01 (CH_3).

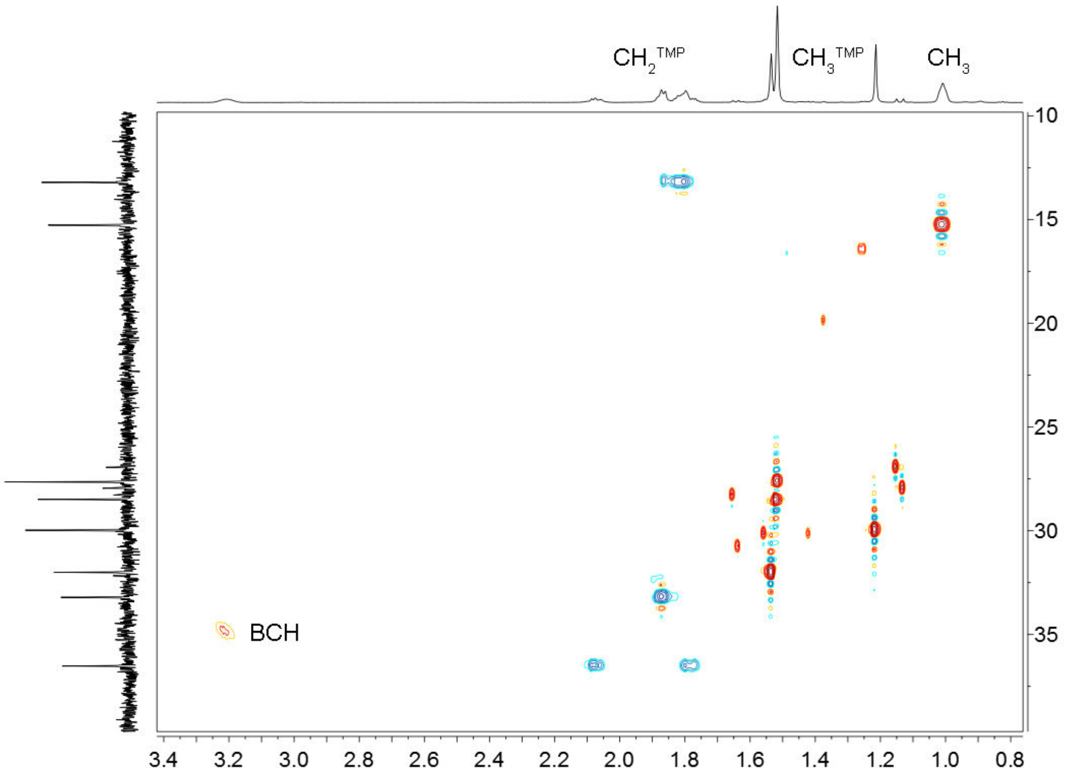

Figure S6. $^{1}H,^{13}C$ ghsqc (600/151 MHz, 238 K, CD_2Cl_2) spectrum of compound **11**.

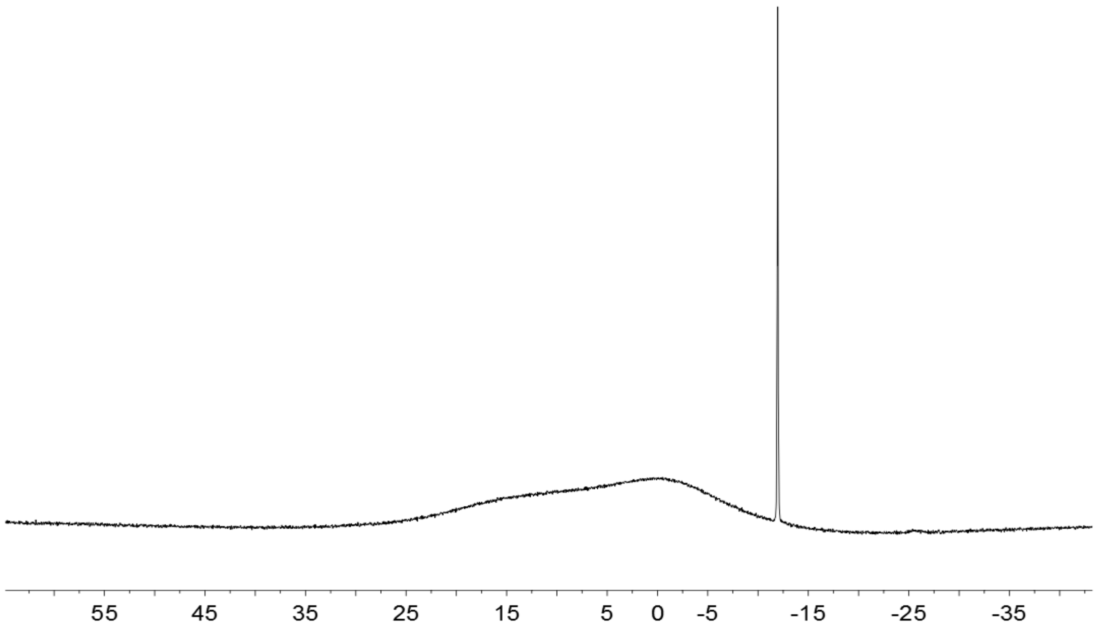

Figure S7. $^{11}B\{^{1}H\}$ NMR (192 MHz, 238 K, CD_2Cl_2) spectrum of compound **11**.

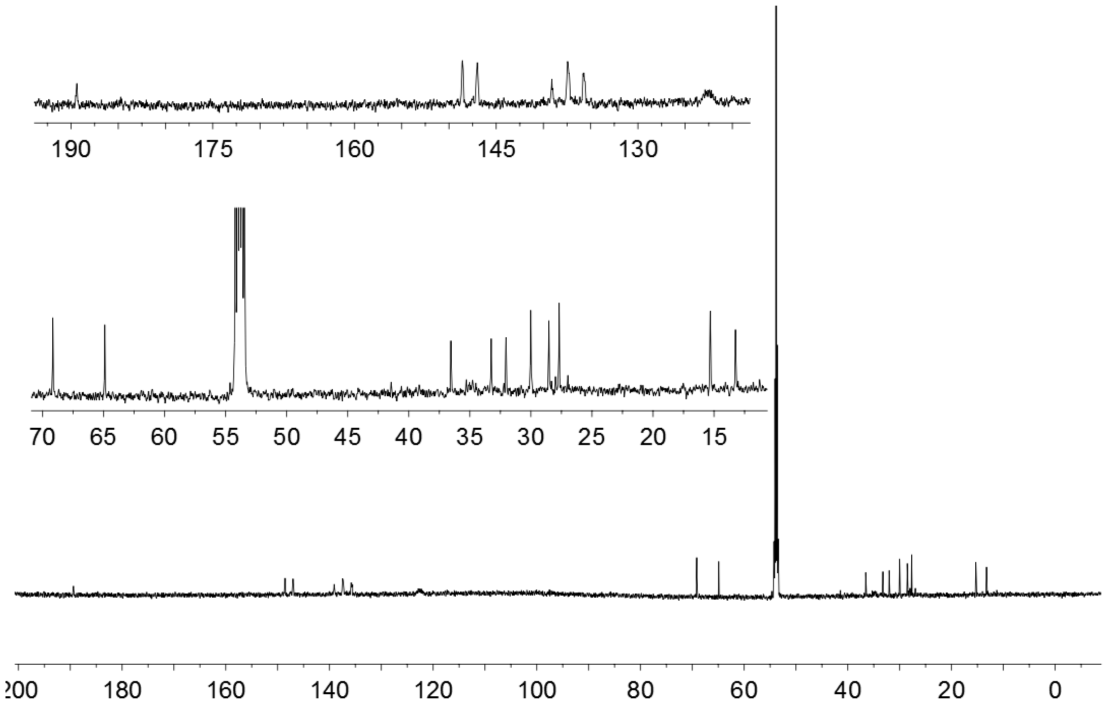

Figure S8. $^{13}C\{^{1}H\}$ NMR (151 MHz, 238 K, CD_2Cl_2) spectrum of compound **11**.

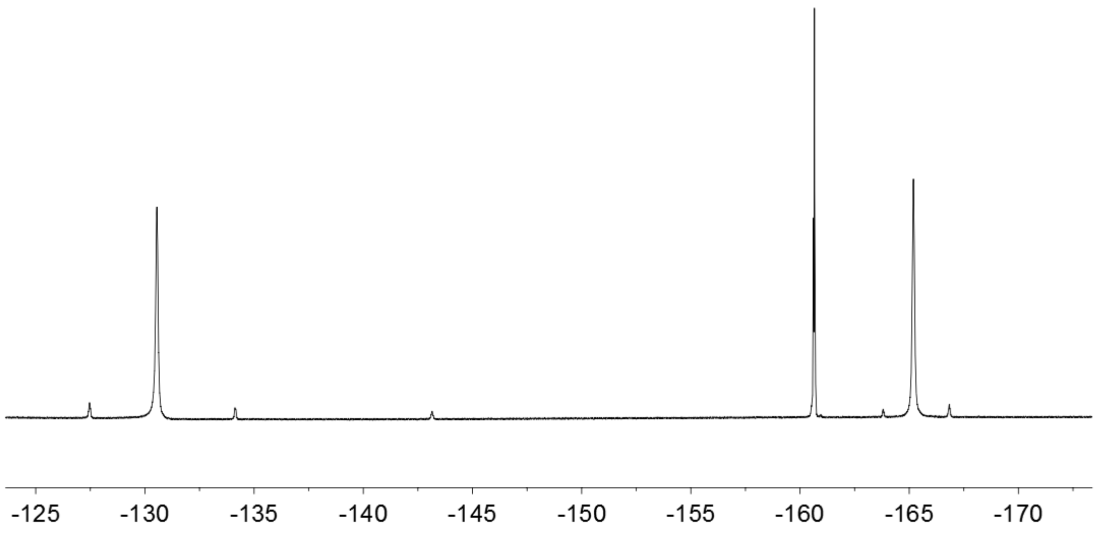

Figure S9. ^{19}F NMR (564 MHz, 238 K, CD_2Cl_2) spectrum of compound **11**.

Crystals suitable for the X-ray crystal structure analysis were obtained from a two-layer procedure using a solution of compound **11** in CH_2Cl_2 covered with *n*-pentane at -35 °C.

X-ray crystal structure analysis of compound 11: A colorless plate-like specimen of $C_{30}H_{23}BF_{15}N$, approximate dimensions 0.044 mm x 0.217 mm x 0.388 mm, was used for the X-ray crystallographic analysis. The X-ray intensity data were measured. A total of 492 frames were collected. The total exposure time was 5.47 hours. The frames were integrated with the Bruker SAINT software package using a narrow-frame algorithm. The integration of the data using a monoclinic unit cell yielded a total of 38910 reflections to a maximum θ angle of 24.71° (0.85 Å resolution), of which 9575 were independent (average redundancy 4.064, completeness = 99.8%, R_{int} = 13.74%, R_{sig} = 10.90%) and 6526 (68.16%) were greater than 2σ(F^2). The final cell constants of a = 9.1176(12) Å, b = 16.071(2) Å, c = 19.360(2) Å, β = 98.211(4)°, volume = 2807.7(6) $Å^3$, are based upon the refinement of the XYZ-centroids of 8325 reflections above 20 σ(I) with 4.706° < 2θ < 48.56°. Data were corrected for absorption effects using the multi-scan method (SADABS). The ratio of minimum to maximum apparent transmission was 0.702. The calculated minimum and maximum transmission coefficients (based on crystal size) are 0.9390 and 0.9930. The structure was solved and refined using the Bruker SHELXTL Software Package, using the space group P 1 21 1, with Z = 4 for the

formula unit, $C_{30}H_{23}BF_{15}N$. The final anisotropic full-matrix least-squares refinement on F^2 with 857 variables converged at R1 = 6.12%, for the observed data and wR2 = 12.84% for all data. The goodness-of-fit was 1.045. The largest peak in the final difference electron density synthesis was 0.442 $e^-/Å^3$ and the largest hole was -0.373 $e^-/Å^3$ with an RMS deviation of 0.074 $e^-/Å^3$. On the basis of the final model, the calculated density was 1.640 g/cm^3 and F(000), 1400 e^-.

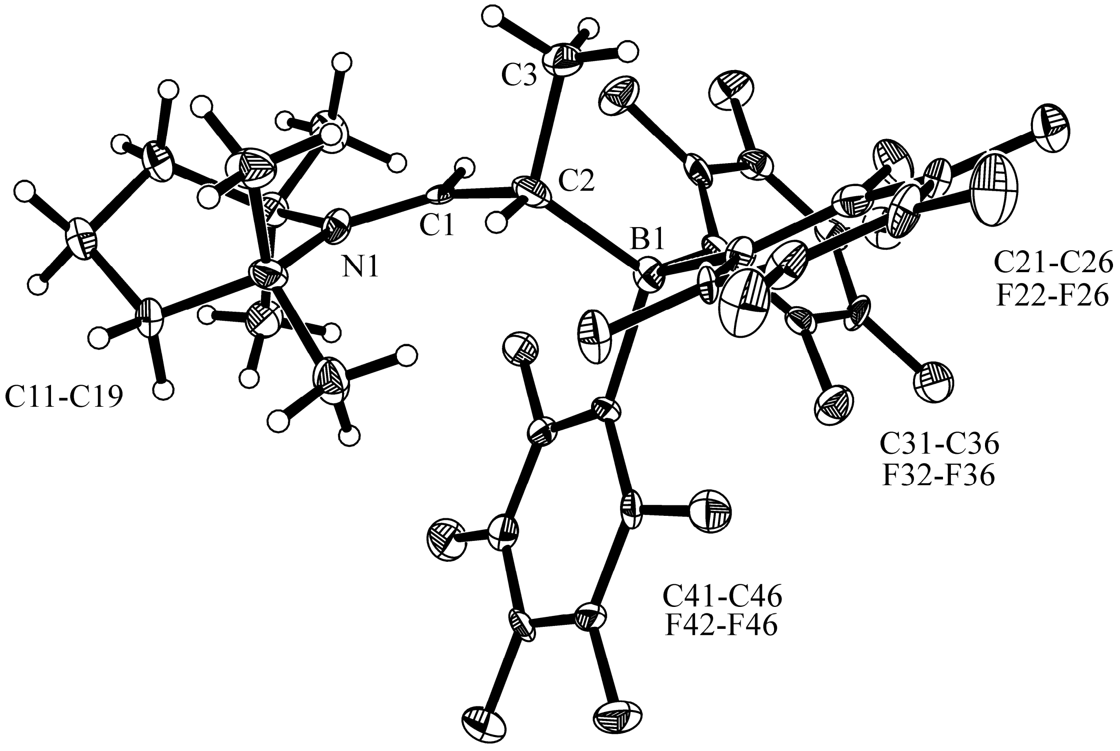

Figure S10. A view of the molecular structure of compound **11**.

Synthesis of compound 12a

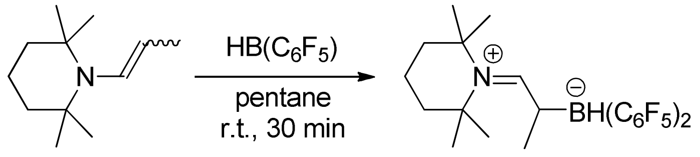

A solution of trans-/cis-**10** (181.5 mg, 1.00 mmol) in *n*-pentane (2 mL) was added to a suspension of $HB(C_6F_5)_2$ (346.0 mg, 1.00 mmol) in *n*-pentane (20 mL), which resulted in a yellow solution after 10 min. The mixture was stirred at room temperature for 30 min to give a white precipitate. The precipitate was collected by filtration and dried in vacuo to give a white solid. Yield: 458.8 mg, 0.87mmol, 87%.

Anal. Calcd. for $C_{24}H_{24}F_{10}NB$: C, 54.67; H, 4.59; N, 2.66. Found: C, 54.32; H, 4.55; N, 2.32.

Mp (DSC): 126°C

[TMP: 2,2,6,6-tetramethylpiperidino]

^{1}H NMR (600 MHz, 299 K, CD_2Cl_2): δ = 8.12 (d, $^3J_{HH}$ = 13.1 Hz, 1H, N=CH), 3.32 (m, 1H, BCH), 2.74 (br 1:1:1:1 q[a], $^1J_{BH}$ ~ 93 Hz, 1H, BH), 1.97/1.85 (each 1H), 1.83 (2H)(each m, $^CCH_2{}^{TMP}$), 1.86/1.79 (each m, each 1H, $CH_2{}^{TMP}$), 1.63/1.33, 1.52/1.48 (each s, each 3H, $CH_3{}^{TMP}$), 1.18 (d, $^3J_{HH}$ = 5.8 Hz, 3H, CH_3), [[a] partially relaxed].

^{13}C{^{1}H} NMR (151 MHz, 299 K, CD_2Cl_2): δ = 189.2 (N=CH), 67.8, 65.1 (NC^{TMP}), 38.1, 34.9 ($^CCH_2{}^{TMP}$), 37.4 (br, BCH), 32.0/30.7, 29.51/29.46 ($CH_3{}^{TMP}$), 16.3 (CH_3), 14.3 ($CH_2{}^{TMP}$), [C_6F_5 not listed].

^{11}B{^{1}H} NMR (192 MHz, 299 K, CD_2Cl_2): δ = -20.0 ($\nu_{1/2}$ ~ 40 Hz).

^{11}B NMR (192 MHz, 299 K, CD_2Cl_2): δ = -20.0 (d, $^{1}J_{HB}$ ~ 93 Hz).

^{19}F NMR (564 MHz, 299 K, CD_2Cl_2): δ = -131.8 (m, 2F, o-C_6F_5), -161.8 (t, $^{3}J_{FF}$ = 20.0 Hz, 1F, p-C_6F_5), -165.9 (m, 2F, m-C_6F_5)[$\Delta\delta^{19}F_{m,p}$ = 4.1]; -132.2 (m, 2F, o-C_6F_5), -162.3 (t, $^{3}J_{FF}$ = 20.0 Hz, 1F, p-C_6F_5), -166.1 (m, 2F, m-C_6F_5)[$\Delta\delta^{19}F_{m,p}$ = 3.8].

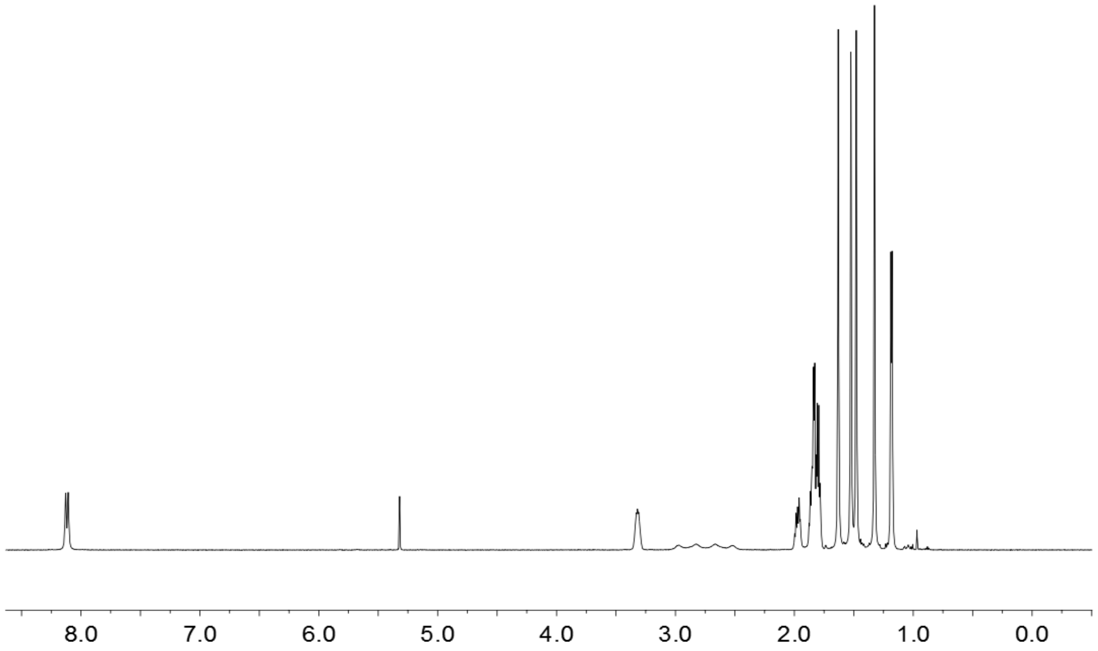

Figure S11. ^{1}H NMR (600 MHz, 299 K, CD_2Cl_2) spectrum of compound **12a**.

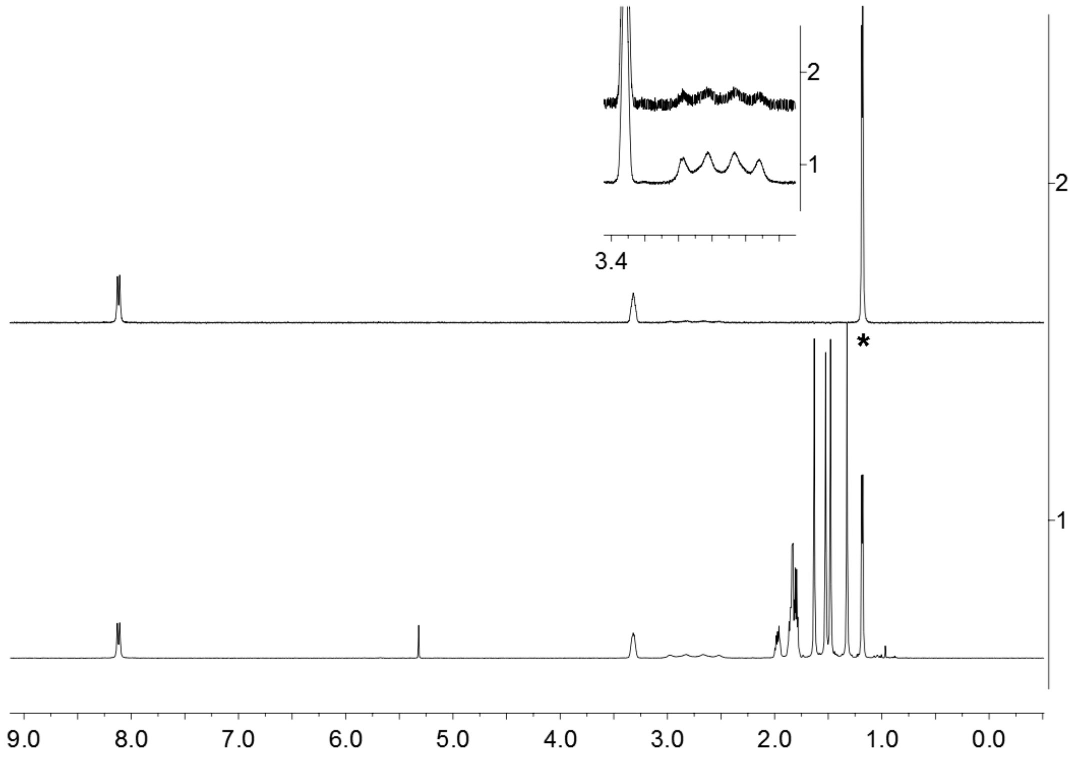

Figure S12. (1) ^{1}H NMR and (2) ^{1}H{^{1}H} TOCSY (600 MHz, 299 K, CD_2Cl_2) spectra of compound **12a**. * Irradiation point: $^1H_{irr}$ = 1.18 (CH_3).

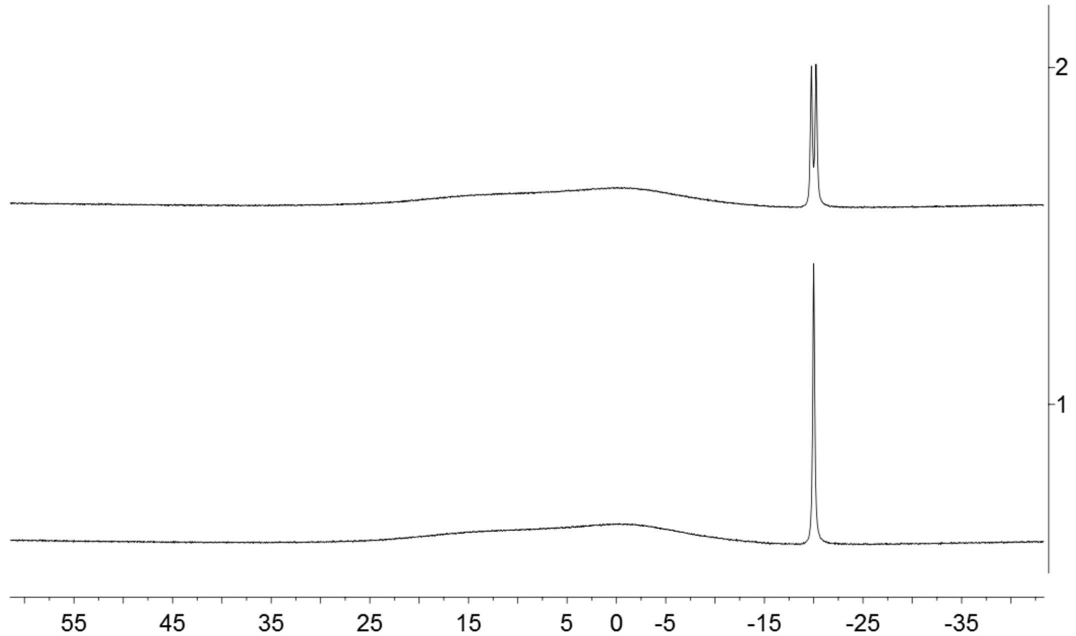

Figure S13. (1) ^{11}B{^{1}H} and (2) ^{11}B NMR (192 MHz, 299 K, CD_2Cl_2) spectra of compound **12a**.

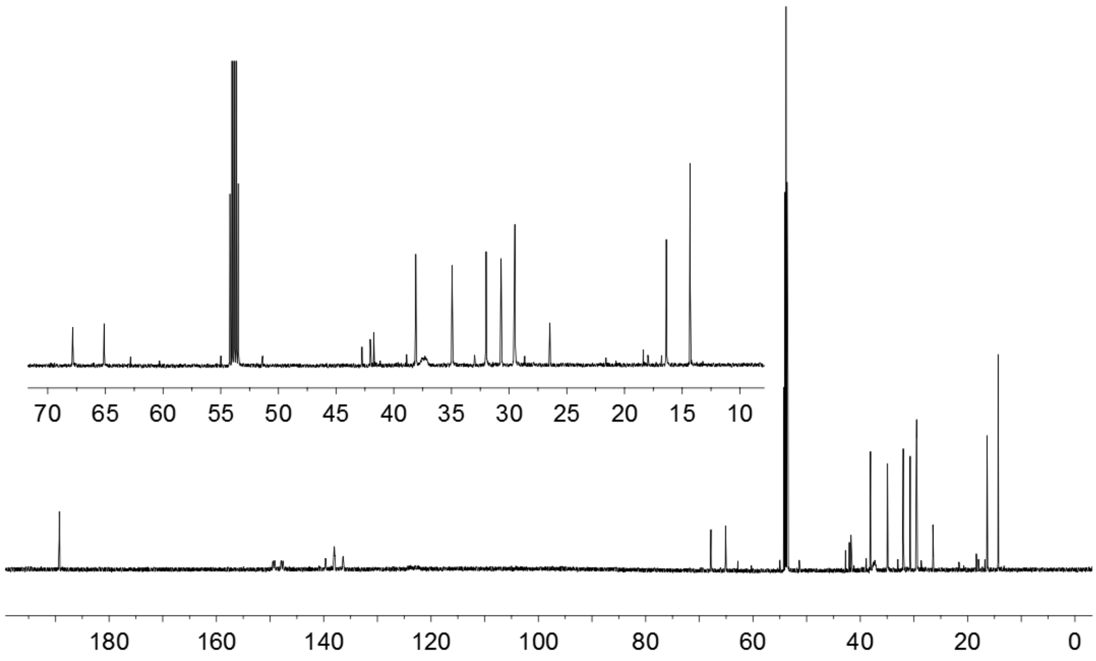

Figure S14. $^{13}C\{^{1}H\}$ NMR (151 MHz, 299 K, CD_2Cl_2) spectrum of compound **12a**.

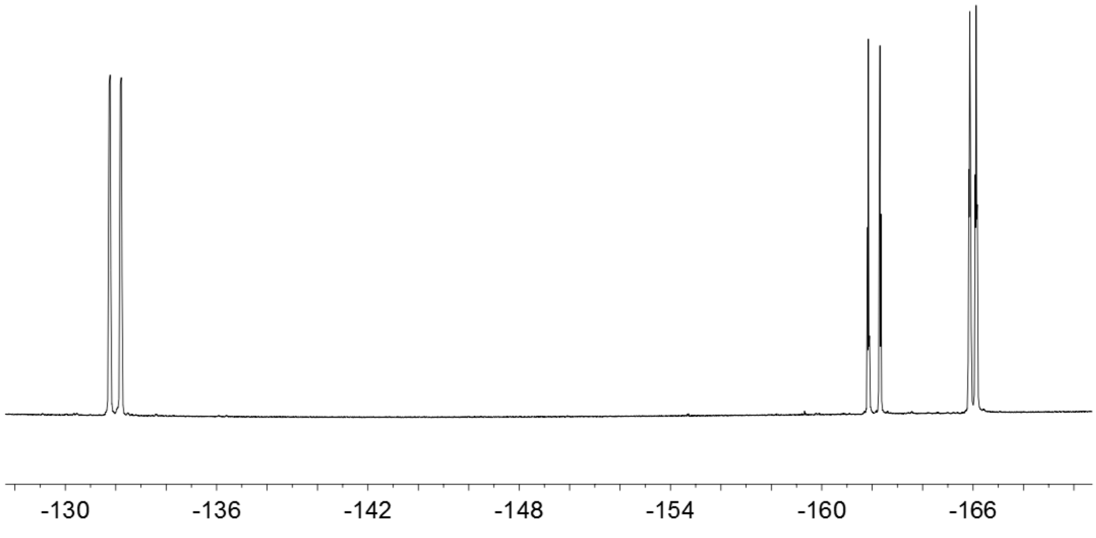

Figure S15. ^{19}F NMR (564 MHz, 299 K, CD_2Cl_2) spectrum of compound **12a**.

Crystals suitable for the X-ray crystal structure analysis were obtained from a two-layer procedure using a solution of compound **12a** in CH_2Cl_2 covered with *n*-pentane at -35 °C.

X-ray crystal structure analysis of compound 12a: A colorless prism-like specimen of $C_{24}H_{24}BF_{10}N$, approximate dimensions 0.044 mm x 0.093 mm x 0.152 mm, was used for the X-ray crystallographic analysis. The X-ray intensity data were measured. A total of 342 frames were collected. The total exposure time was 5.70 hours. The frames were integrated with the Bruker SAINT software package using a narrow-frame algorithm. The integration of the data using a triclinic unit cell yielded a total of 14961 reflections to a maximum θ angle of 26.37° (0.80 Å resolution), of which 4710 were independent (average redundancy 3.176, completeness = 99.7%, R_{int} = 5.38%, R_{sig} = 5.53%) and 3370 (71.55%) were greater than $2\sigma(F^2)$. The final cell constants of a = 9.6021(5) Å, b = 9.8581(6) Å, c = 13.1600(7) Å, α = 73.124(2)°, β = 77.164(2)°, γ = 81.941(2)°, volume = 1158.44(11) $Å^3$, are based upon the refinement of the XYZ-centroids of 4139 reflections above 20 σ(I) with 4.686° < 2θ < 54.84°. Data were corrected for absorption effects using the multi-scan method (SADABS). The ratio of minimum to maximum apparent transmission was 0.962. The calculated minimum and maximum transmission coefficients (based on crystal size) are 0.9780 and 0.9940. The final anisotropic full-matrix least-squares refinement on F^2 with 334

variables converged at R1 = 4.27%, for the observed data and wR2 = 9.76% for all data. The goodness-of-fit was 1.014. The largest peak in the final difference electron density synthesis was 0.292 $e^{-}/Å^{3}$ and the largest hole was -0.306 $e^{-}/Å^{3}$ with an RMS deviation of 0.055 $e^{-}/Å^{3}$. On the basis of the final model, the calculated density was 1.512 g/cm^{3} and F(000), 540 e^{-}.

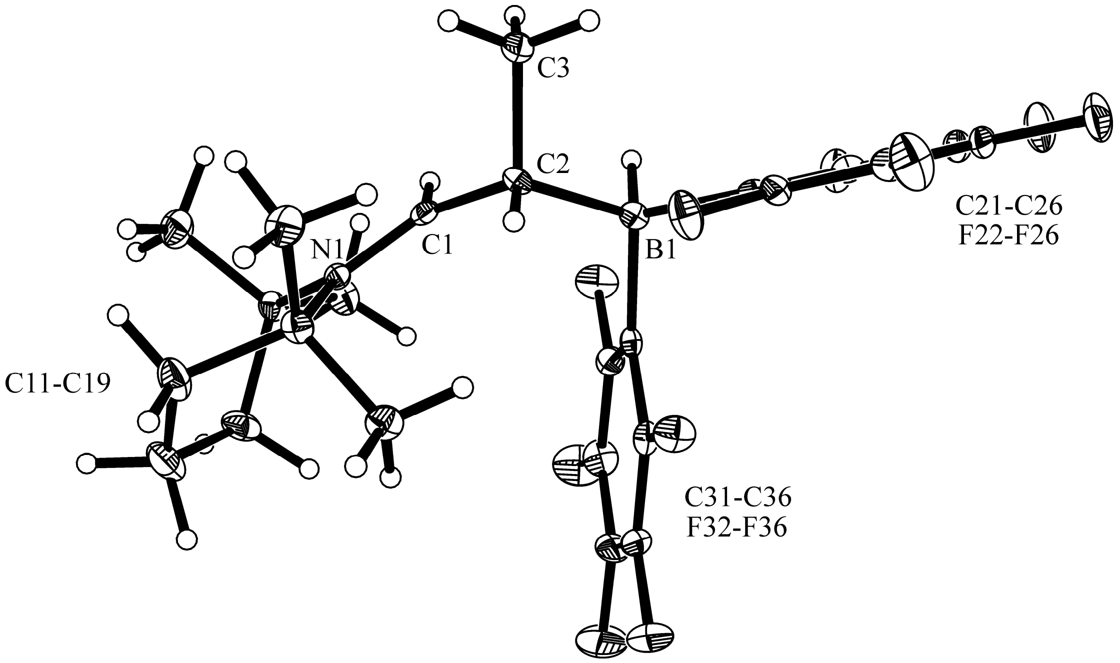

Figure S16. A view of the molecular structure of compound **12a**.

Synthesis of compound 13a

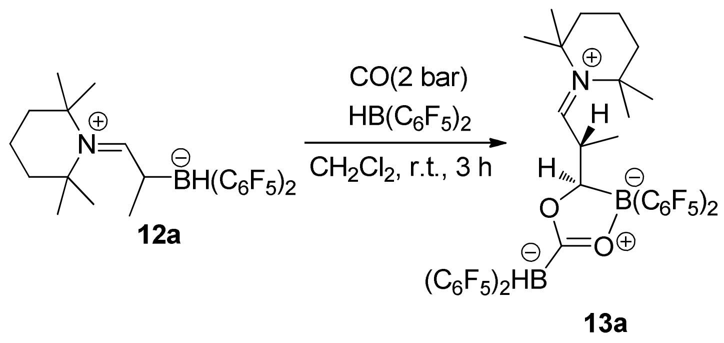

Method A (*with additional* $HB(C_6F_5)_2$): Compound **12a** (105.5 mg, 0.20 mmol) and $HB(C_6F_5)_2$ (69.2 mg, 0.20 mmol) were weighed together, then CH_2Cl_2 (5 mL) was added to give a colorless solution. The solution was degassed by applying vacuum carefully at r.t. After that, CO (2.0 bar) was pressed over the solution, and the mixture was stirred at room temperature for 3 h. Then all the volatiles were removed under reduced pressure. The obtained residue was washed with CH_2Cl_2 (3×2 mL) and dried in vacuo to give a white solid. Yield: 75.6 mg, 0.08 mmol, 41%.

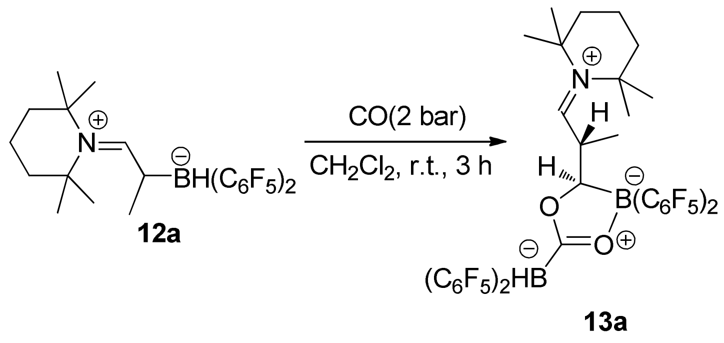

Method B (*without additional* $HB(C_6F_5)_2$): Compound **12a** (158.2 mg, 0.30 mmol) was dissolved in CH_2Cl_2 (3 mL) to give a colorless solution. The solution was degassed by applying vacuum carefully at r.t.. Then CO (2.0 bar) was pressed over the solution, and the mixture was stirred at

room temperature for 3 h. All the volatiles were removed under reduced pressure. The obtained residue was washed with *n*-pentane (3×3 mL) and dried in vacuo to give a white solid. Yield: 119.9 mg, 0.13 mmol, 43%.

Anal. Calcd. for $C_{38}H_{25}F_{20}NO_2B_2$: C, 49.12; H, 2.71; N, 1.51. Found: C, 48.65; H, 2.48; N, 1.53.

Decomp. (DSC): 216°C

[TMP: 2,2,6,6-tetramethylpiperidino]

^{1}H NMR (600 MHz, 299 K, THF-d_8): δ = 8.68 (d, $^3J_{HH}$ = 10.4 Hz, 1H, N=CH), 5.10 (d, $^3J_{HH}$ = 11.0 Hz, 1H, BCH), 3.48 (m, 1H, CH), 3.45 (br 1:1:1:1 q[a], $^1J_{BH}$ ~ 90 Hz, 1H, BH), 2.11/1.97, 1.92/1.76 (each m, each 1H, $^C CH_2^{TMP}$), 1.93[t]/1.83 (each m, each 1H, CH_2^{TMP}), 1.64/1.54, 1.63/1.27 (each s, each 3H, CH_3^{TMP}), 1.11 (d, $^3J_{HH}$ = 6.5 Hz, 3H, CH_3), [[t] tentative assignment; [a] partially relaxed]

^{13}C{^{1}H} NMR (151 MHz, 299 K, THF-d_8): δ = 213.7 (br, OC=O), 184.5 (N=CH), 86.2 (br, BCH), 73.4, 69.6 (NC^{TMP}), 44.0 (CH), 38.0, 34.6 ($^C CH_2^{TMP}$), 31.9/28.5, 31.6/30.0 (CH_3^{TMP}), 14.5 (CH_2^{TMP}), 12.2 (m, CH_3), [C_6F_5 not listed].

^{11}B{^{1}H} NMR (192 MHz, 299 K, THF-d_8): δ = 2.6 ($\nu_{1/2}$ ~ 300 Hz), -26.1 ($\nu_{1/2}$ ~ 60 Hz).

^{11}B NMR (192 MHz, 299 K, THF-d_8): δ = 2.6 ($\nu_{1/2}$ ~ 300 Hz), -26.1 (d, $^1J_{HB}$ ~ 86 Hz).

^{19}F NMR (564 MHz, 299 K, THF-d_8): δ = -130.9 (m, 2F, *o*-C_6F_5), -162.5

(t, $^3J_{FF}$ = 19.9 Hz, 1F, *p*-C_6F_5), -166.8 (m, 2F, *m*-C_6F_5)[$\Delta\delta^{19}F_{m,p}$ = 4.3]; -131.4 (m, 2F, *o*-C_6F_5), -162.7 (t, $^3J_{FF}$ = 19.9 Hz, 1F, *p*-C_6F_5), -167.4 (m, 2F, *m*-C_6F_5)[$\Delta\delta^{19}F_{m,p}$ = 3.7]; -133.69 (m, 2F, *o*-C_6F_5), -159.5 (t, $^3J_{FF}$ = 20.1 Hz, 1F, *p*-C_6F_5), -166.4 (m, 2F, *m*-C_6F_5)[$\Delta\delta^{19}F_{m,p}$ = 6.9]; -133.74 (m, 2F, *o*-C_6F_5), -157.6 (t, $^3J_{FF}$ = 20.1 Hz, 1F, *p*-C_6F_5), -164.5 (m, 2F, *m*-C_6F_5)[$\Delta\delta^{19}F_{m,p}$ = 6.9].

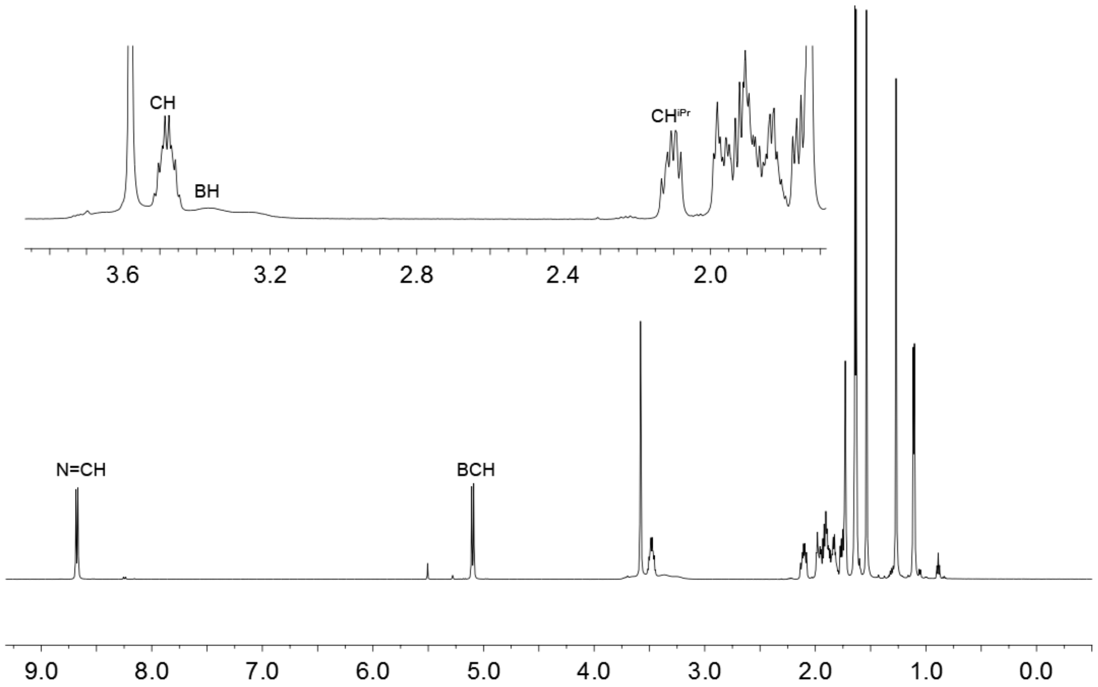

Figure S17. ^{1}H NMR (600 MHz, 299 K, THF-d_8) spectrum of compound **13a**.

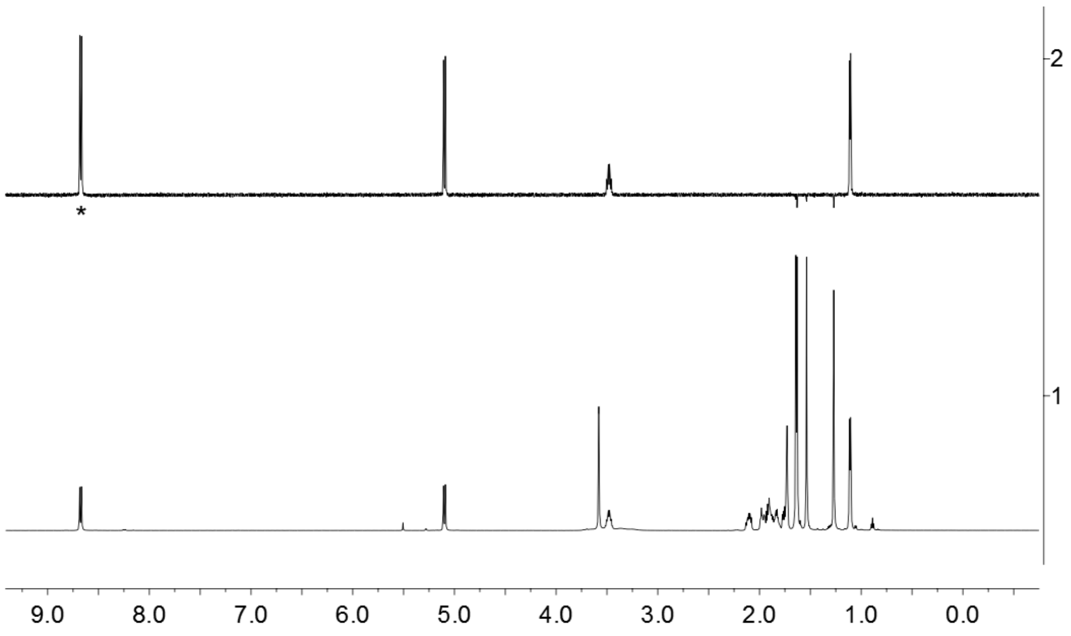

Figure S18. (1) ^{1}H NMR and (2) $^{1}H\{^{1}H\}$ TOCSY (600 MHz, 299 K, THF-d_8) spectra of compound **13a**. * Irradiation point: $^{1}H_{irr}$ = 8.68 (N=CH).

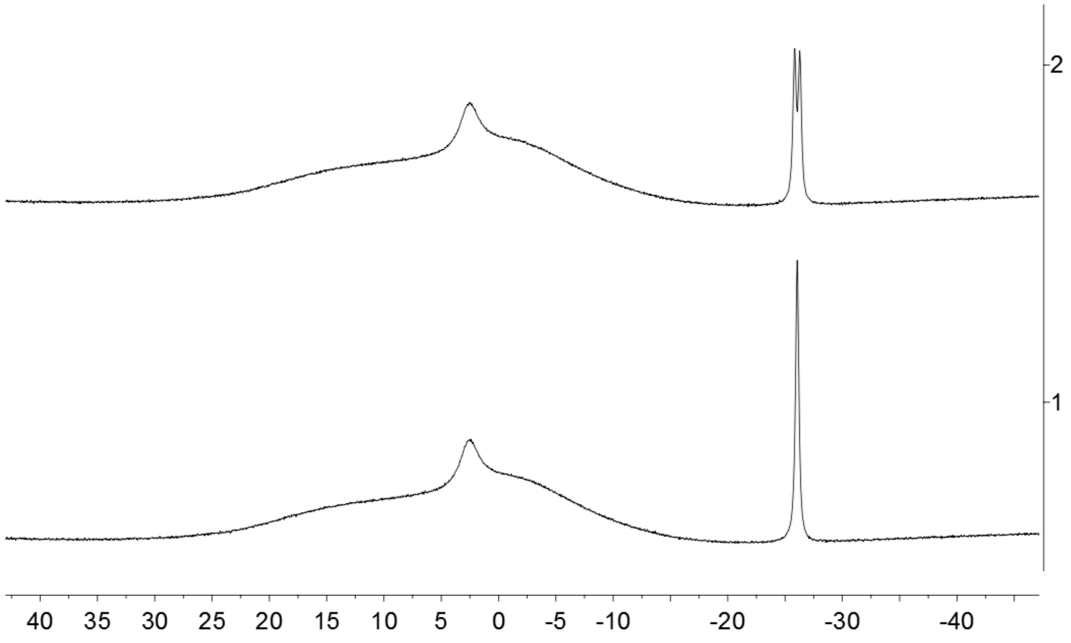

Figure S19. (1) $^{11}B\{^{1}H\}$ NMR and (2) ^{11}B NMR (192 MHz, 299 K, THF-d_8) spectra of compound **13a**.

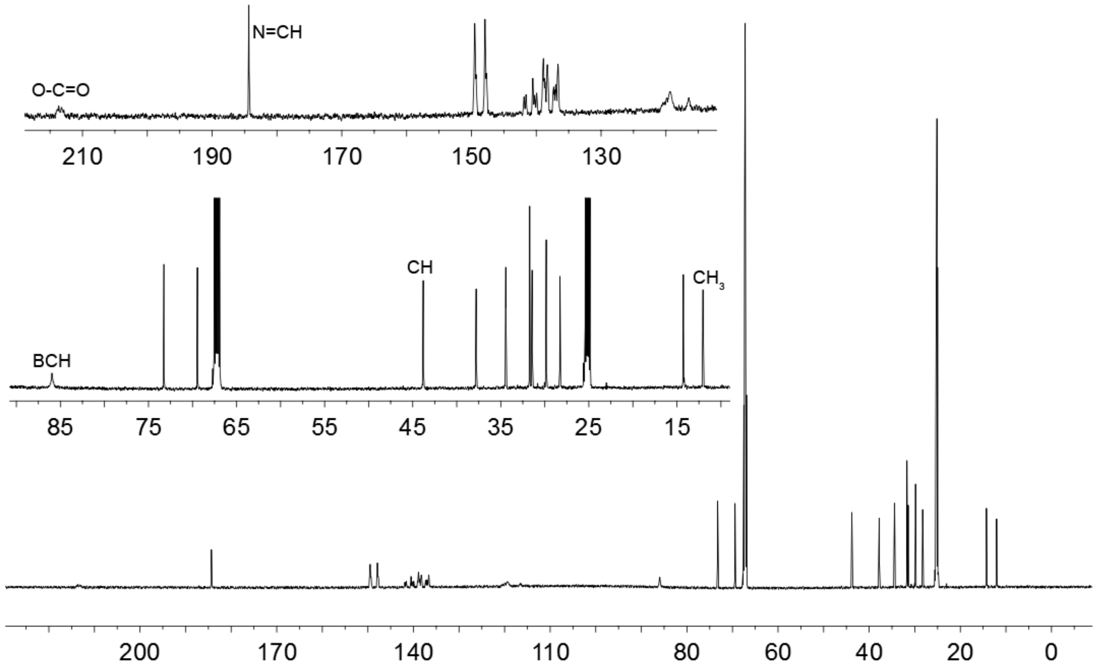

Figure S20. $^{13}C\{^1H\}$ NMR (151 MHz, 299 K, THF-d$_8$) spectrum of compound **13a**.

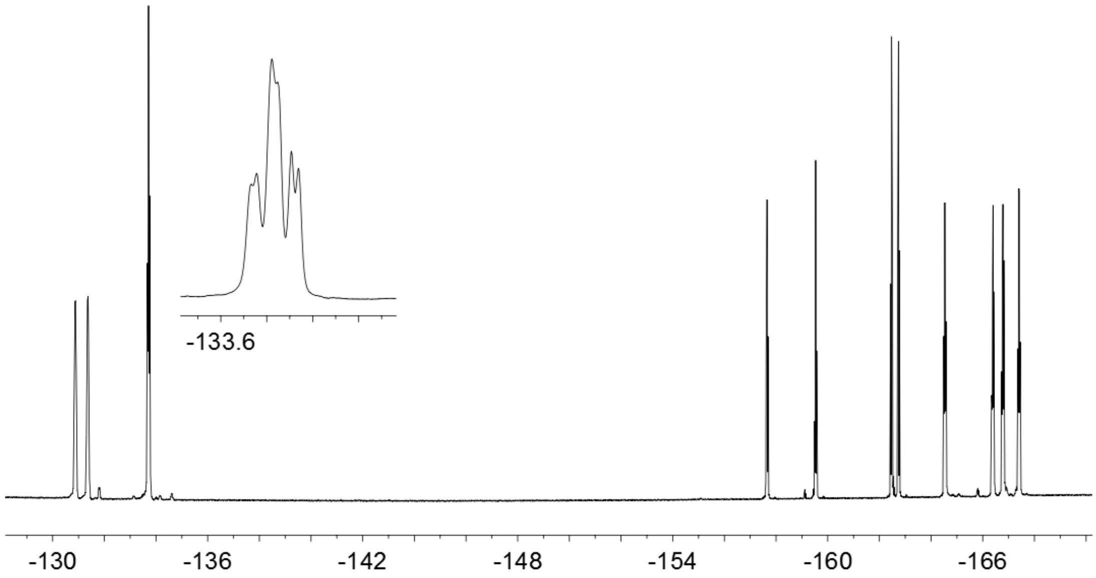

Figure S21. ^{19}F NMR (564 MHz, 299 K, THF-d$_8$) spectrum of compound **13a**.

Crystals suitable for the X-ray crystal structure analysis were obtained from a two-layer procedure using a solution of compound **13a** in THF covered with *n*-pentane at -35 °C.

X-ray crystal structure analysis of compound 13a: A colorless prism-like specimen of $C_{38}H_{25}B_2F_{20}NO_2$, approximate dimensions 0.064 mm x 0.082 mm x 0.168 mm, was used for the X-ray crystallographic analysis. The X-ray intensity data were measured. A total of 406 frames were collected. The total exposure time was 4.51 hours. The frames were integrated with the Bruker SAINT software package using a narrow-frame algorithm. The integration of the data using a monoclinic unit cell yielded a total of 47361 reflections to a maximum θ angle of 25.35° (0.83 Å resolution), of which 6661 were independent (average redundancy 7.110, completeness = 99.9%, R_{int} = 8.30%, R_{sig} = 4.67%) and 4857 (72.92%) were greater than $2\sigma(F^2)$. The final cell constants of a = 14.4987(7) Å, b = 11.0883(4) Å, c = 23.6041(10) Å, β = 106.5030(10)°, volume = 3638.4(3) $Å^3$, are based upon the refinement of the XYZ-centroids of 9910 reflections above 20 σ(I) with 4.724° < 2θ < 51.72°. Data were corrected for absorption effects using the multi-scan method (SADABS). The ratio of minimum to maximum apparent transmission was 0.939. The calculated minimum and maximum transmission coefficients (based on crystal size) are 0.9710 and 0.9890. The final anisotropic full-matrix least-squares refinement on F^2 with 577

variables converged at R1 = 4.05%, for the observed data and wR2 = 8.67% for all data. The goodness-of-fit was 1.033. The largest peak in the final difference electron density synthesis was 0.296 $e^-/Å^3$ and the largest hole was -0.291 $e^-/Å^3$ with an RMS deviation of 0.056 $e^-/Å^3$. On the basis of the final model, the calculated density was 1.696 g/cm^3 and F(000), 1864 e^-.

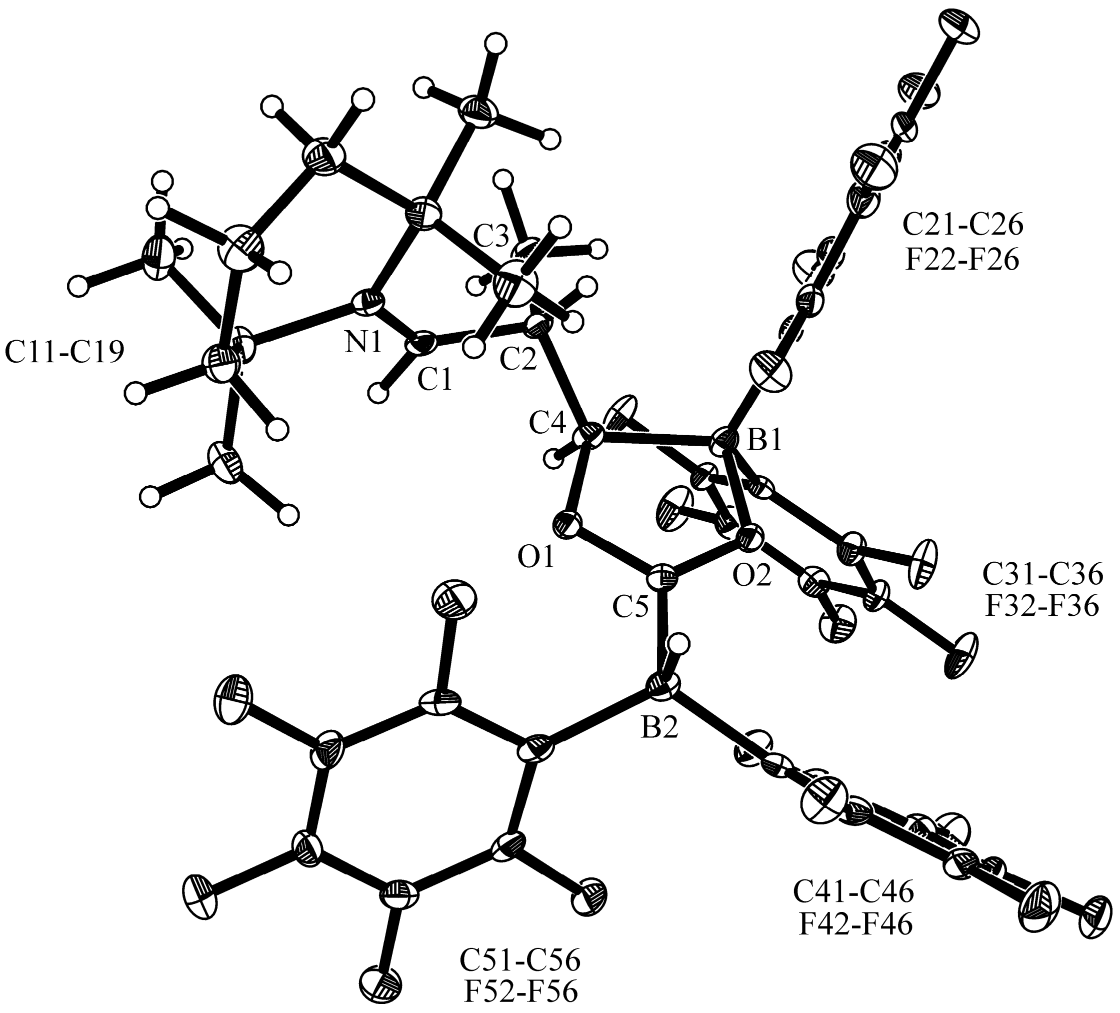

Figure S22. A view of the molecular structure of compound **13a**.

In situ reaction of compound 12a with CO:

generation of compound 13a and trans-/cis-10

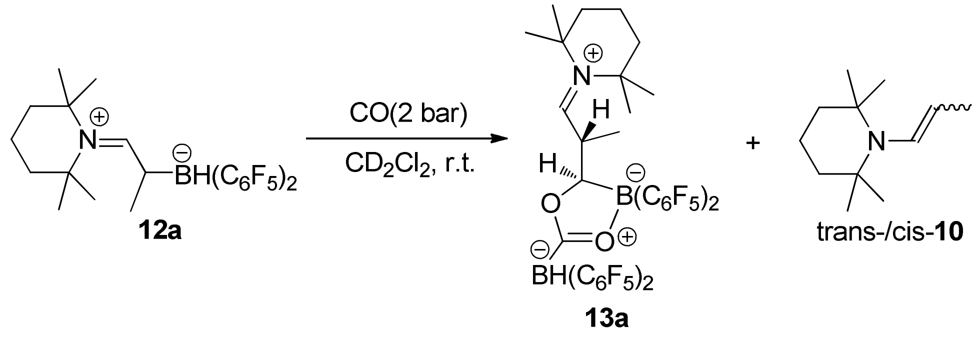

In a Young NMR tube a solution of compound **12a** (25.6 mg, 0.05 mmol) in CD_2Cl_2 (0.6 mL) was degassed, then carefully evacuated and exposed to CO gas (2.0 bar). Subsequently the mixture was characterized by NMR experiments. [Comment: after ca. 2 hours at r.t. the ratio between compound **10** and compound **13a** was ca. 1 : 0.7 (^{1}H)].

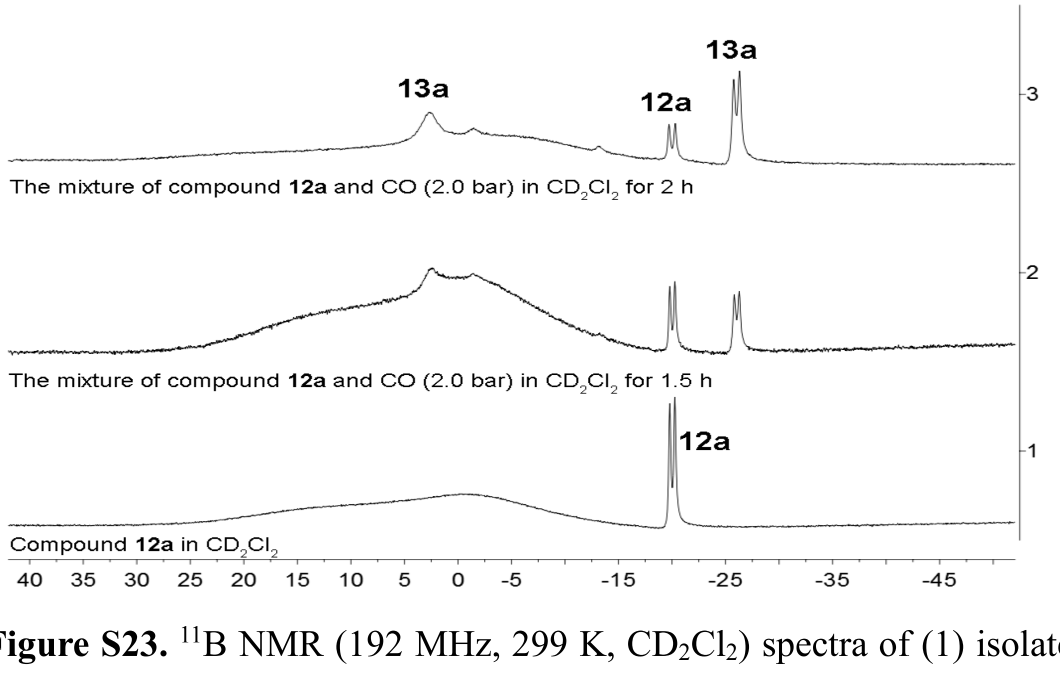

Figure S23. ^{11}B NMR (192 MHz, 299 K, CD_2Cl_2) spectra of (1) isolated compound **12a**, (2) the mixture of compounds **12a** and CO (2 bar) for 1.5 h at r.t., (3) the mixture of compounds **12a** and CO (2 bar) for 2 h at r.t..

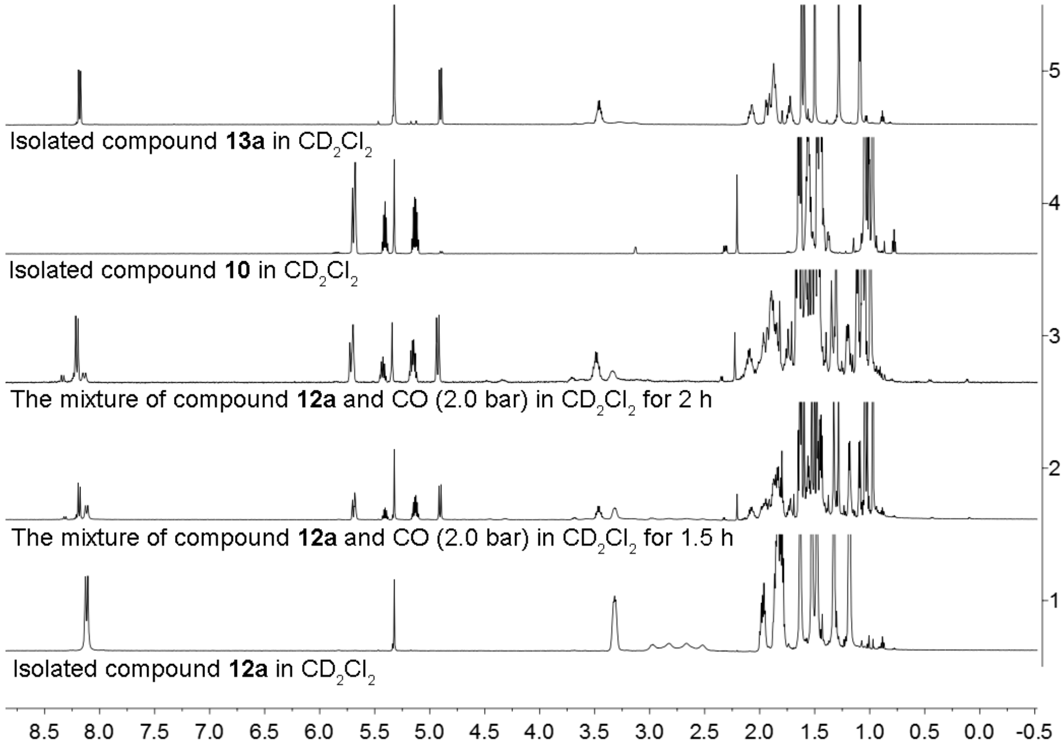

Figure S24. ^{1}H NMR (600 MHz, 299 K, CD_2Cl_2) spectra of (1) isolated compound **12a**, (2) the mixture of compounds **12a** and CO (2 bar) after 1.5 h at r.t., (3) the mixture of compounds **12a** and CO (2 bar) after 2 h at r.t., (4) isolated compound **10**, (5) isolated compound **13a**.

Synthesis of compound 13b

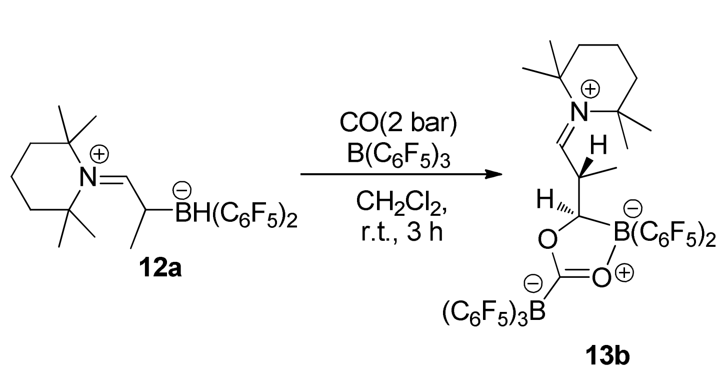

Compound **12a** (105.5 mg, 0.20 mmol) and $B(C_6F_5)_3$ (102.3 mg, 0.20 mmol) were weighed together, then CH_2Cl_2 (5 mL) was added to give a

colorless solution. The solution was degassed by applying vacuum carefully at r.t.. After that, CO (2.0 bar) was pressed over the solution, and the mixture was stirred at room temperature for 3 h. Then all the volatiles were removed under reduced pressure. The obtained residue was washed with the mixture of CH_2Cl_2 and *n*-pentane (V/V = 1:5) (3×3 mL) and dried in vacuo to give a white solid. Yield: 166.3 mg, 0.15 mmol, 76%.

Anal. Calcd. for $C_{44}H_{24}F_{25}NO_2B_2$: C, 48.25; H, 2.21; N, 1.28. Found: C, 48.84; H, 2.57; N, 1.53.

Decomp. (DSC): 198°C

[TMP: 2,2,6,6-tetramethylpiperidino]

^{1}H NMR (500 MHz, 299 K, THF-d_8): δ = 8.84 (d, $^3J_{HH}$ = 9.8 Hz, 1H, N=CH), 5.21 (d, $^3J_{HH}$ = 10.9 Hz, 1H, BCH), 3.42 (m, 1H, CH), 2.12/1.91, 1.98/1.90 (each m, each 1H, $^CCH_2^{TMP}$), 1.93/1.87 (each m, each 1H, CH_2^{TMP}), 1.67/1.49, 1.50/1.37 (each s, each 3H, CH_3^{TMP}), 1.15 (d, $^3J_{HH}$ = 6.4 Hz, 3H, CH_3).

^{13}C{^{1}H} NMR (126 MHz, 299 K, THF-d_8): δ = 209.3 (br 1:1:1:1 q[a], $^1J_{BC}$ ~ 62 Hz, OC=O), 184.8 (N=CH), 88.3 (br, BCH), 73.8, 69.7 (NC^{TMP}), 43.2 (CH), 37.2, 34.2 ($^CCH_2^{TMP}$), 32.1/29.9, 31.9/27.9 (CH_3^{TMP}), 14.2 (CH_2^{TMP}), 12.8 (m, CH_3), [C_6F_5 not listed; [a] partially relaxed].

^{11}B{^{1}H} NMR (160 MHz, 299 K, THF-d_8): δ = 2.6 ($ν_{1/2}$ ~ 400 Hz), -17.7 ($ν_{1/2}$ ~ 25 Hz).

^{19}F NMR (564 MHz, 299 K, THF-d_8): δ = -130.8 (m, 6F, o), -161.5 (t, $^3J_{FF}$ = 20.1 Hz, 3F, p), -166.7 (m, 6F, m)[$B(C_6F_5)_3$][$\Delta\delta^{19}F_{m,p}$ = 5.2]; -133.8 (m, 2F, o), -159.2 (t, $^3J_{FF}$ = 20.1 Hz, 1F, p), -166.3 (m, 2F, m)[$B(C_6F_5)_2$][$\Delta\delta^{19}F_{m,p}$ = 7.1]; -134.4 (m, 2F, o), -157.0 (t, $^3J_{FF}$ = 20.1 Hz, 1F, p), -164.9 (m, 2F, m)[$B(C_6F_5)_2$][$\Delta\delta^{19}F_{m,p}$ = 7.9].

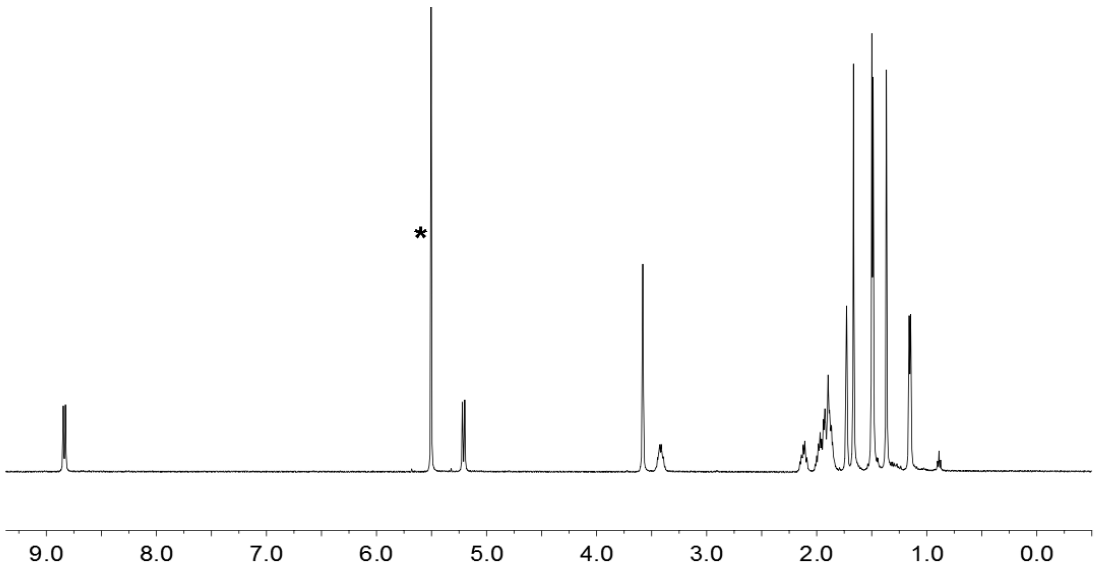

Figure S25. ^{1}H NMR (500 MHz, 299 K, THF-d_8) spectrum of compound **13b**. * δ(CH_2Cl_2): 5.50 (1 equiv.)

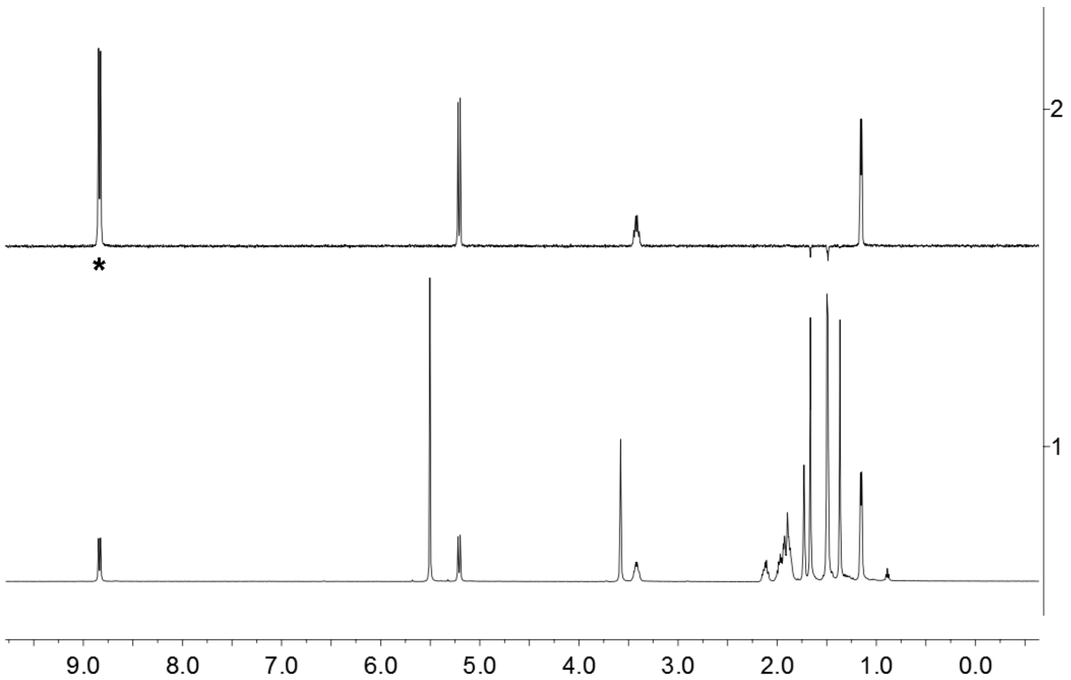

Figure S26. (1) ^{1}H NMR and (2) $^{1}H\{^{1}H\}$ TOCSY (500 MHz, 299 K, THF-d_8) spectra of compound **13b**. * Irradiation point: $^{1}H_{irr}$ = 8.84 (N=CH).

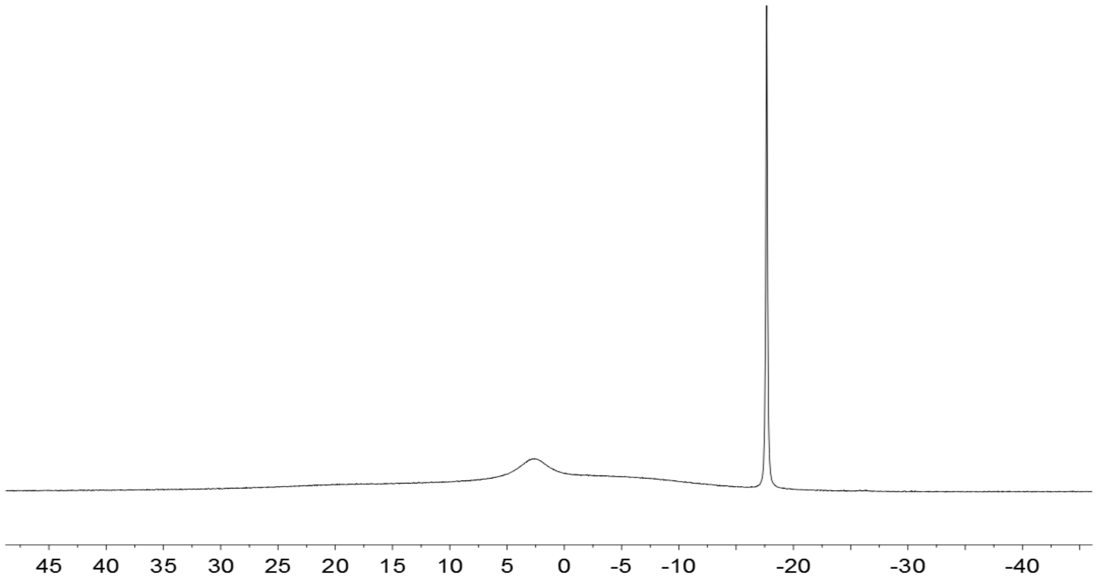

Figure S27. $^{11}B\{^{1}H\}$ NMR (192 MHz, 299 K, THF-d_8) spectrum of compound **13b**.

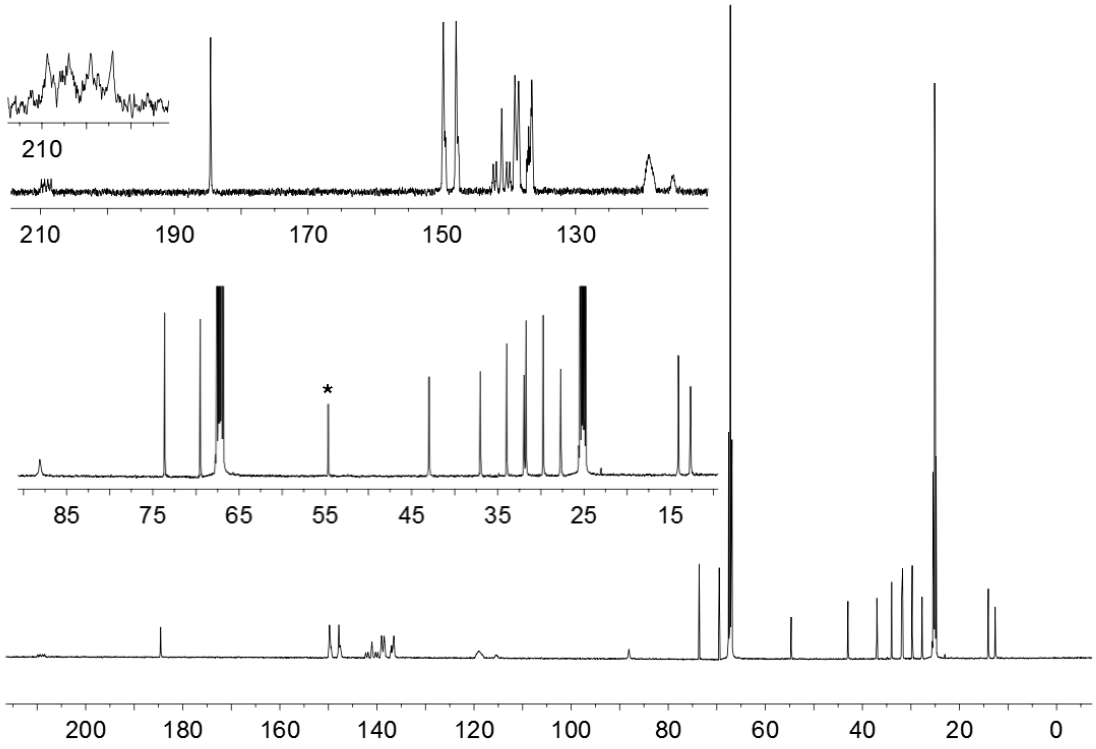

Figure S28. $^{13}C\{^1H\}$ NMR (126 MHz, 299 K, THF-d_8) spectrum of compound **13b**. * δ(CH_2Cl_2): 54.9.

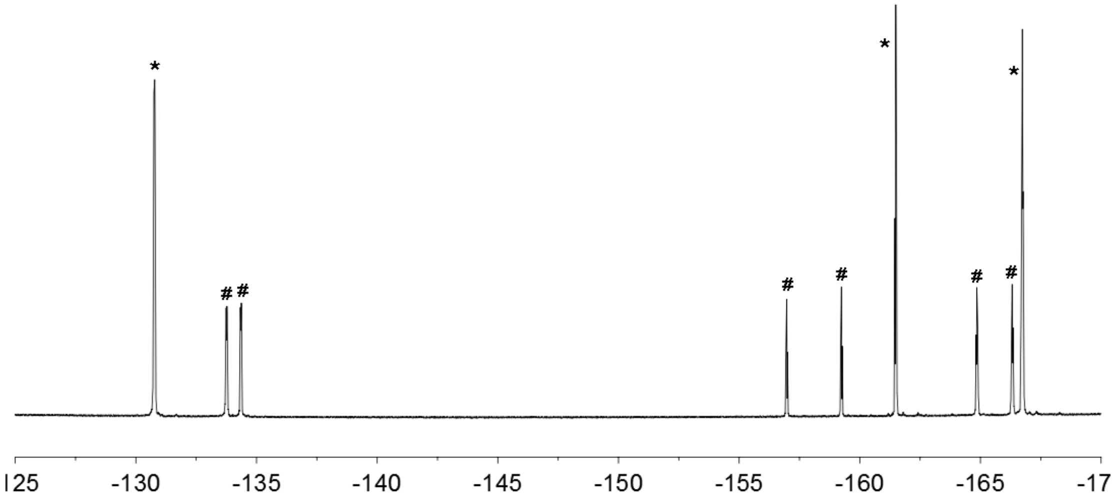

Figure S29. ^{19}F NMR (564 MHz, 299 K, THF-d_8) spectrum of compound **13b**. * $B(C_6F_5)_3$ # $B(C_6F_5)_2$

Crystals suitable for the X-ray crystal structure analysis were obtained from a two-layer procedure using a solution of compound **13b** in THF covered with *n*-pentane at -35 °C.

X-ray crystal structure analysis of compound 13b: formula $C_{44}H_{24}B_2F_{25}NO_2$, M = 1095.26, colourless crystal, 0.16 x 0.06 x 0.03 mm, a = 11.0648(2), b = 22.0848(4), c = 20.1904(4) Å, β = 102.354(2)°, V = 4819.6(2) Å^3, ρ_{calc} = 1.509 gcm^{-3}, μ = 0.158 mm^{-1}, empirical absorption correction ($0.975 \leq T \leq 0.995$), Z = 4, monoclinic, space group $P2_1$/n (No. 14), λ = 0.71073Å, T = 223(2) K, ω and φ scans, 38378 reflections collected ($\pm h$, $\pm k$, $\pm l$), 8350 independent (R_{int} = 0.041) and 5192 observed reflections [I>2σ(I)], 767 refined parameters, R = 0.071, wR^2 = 0.165, max. (min.) residual electron density 0.58 (-0.26) e.Å^{-3}; hydrogen atoms were calculated and refined as riding atoms.

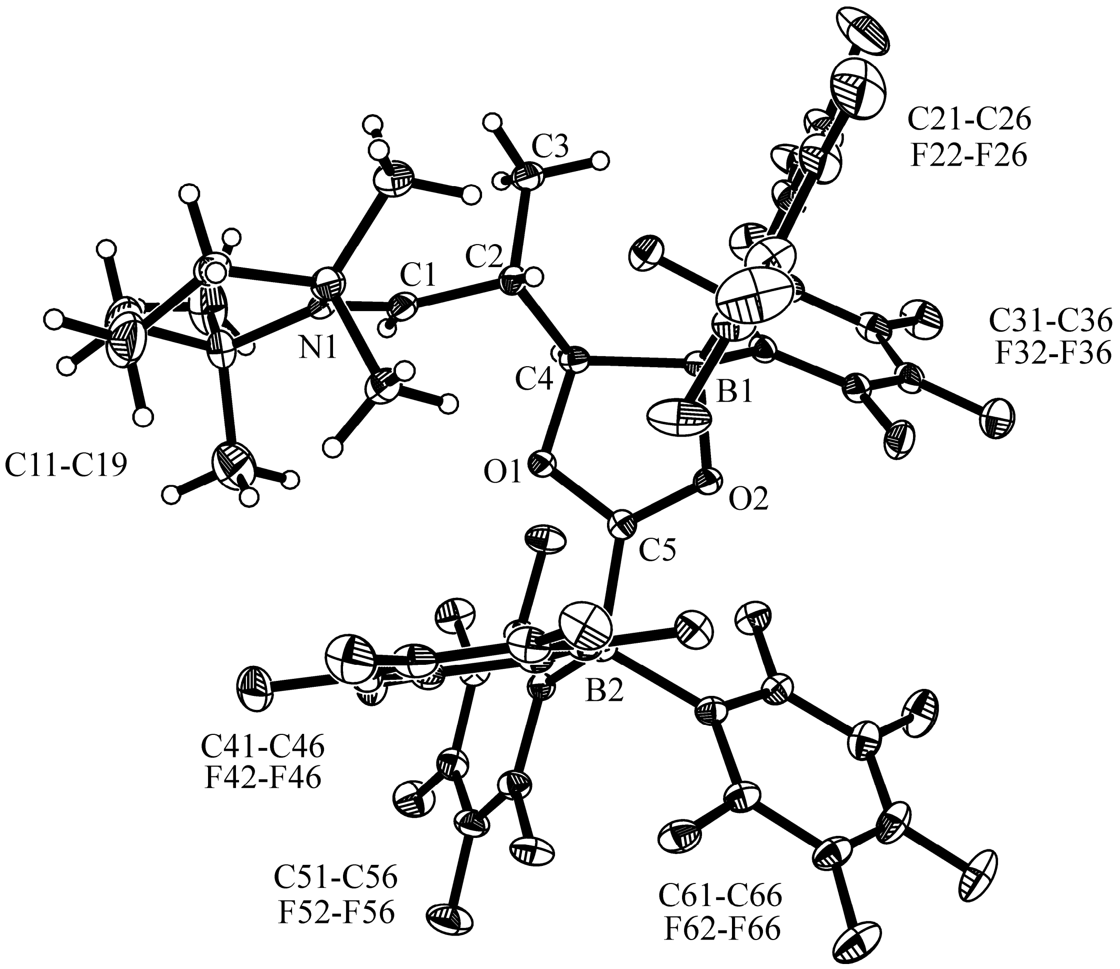

Figure S30. A view of the molecular structure of compound **13b**.

Synthesis of compound 17a

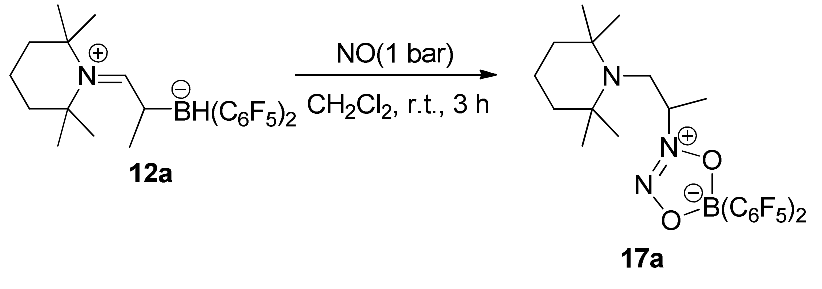

Compound **12a** (265.3 mg, 0.50 mmol) was dissolved in CH_2Cl_2 (5 mL) to give a colorless solution. The solution was degassed by applying vacuum carefully at r.t.. Then NO (1.0 bar) was pressed over the solution,

and the mixture was stirred at room temperature for 3 h. All the volatiles were removed under reduced pressure and the obtained residue was extracted with *n*-pentane (3×30 mL). The pentane solution was filtered and the solvent was removed from the filtrate in vacuo to give a pale yellow solid. Yield: 224.6 mg, 0.38 mmol, 76%.

Anal. Calcd. for $C_{24}H_{24}F_{10}N_3O_2B$: C, 49.08; H, 4.12; N, 7.16. Found: C, 48.92; H, 3.95; N, 7.16.

Mp (DSC): 109 °C

a) NMR data of compound 17a at 233 K

[TMP: 2,2,6,6-tetramethylpiperidino]

^{1}H NMR (600 MHz, 233 K, CD_2Cl_2): δ = 4.80 (m, CH), 3.05 (dd, $^{2}J_{HH}$ = 16.4 Hz, $^{3}J_{HH}$ = 9.5 Hz), 2.72 (d, $^{2}J_{HH}$ = 16.4 Hz)(each 1H, NCH_2), 1.55 (d, $^{3}J_{HH}$ = 6.6 Hz, 3H, CH_3), 1.53/1.32 (each m, each 1H, CH_2^{TMP}), 1.39/1.34, 1.23/1.14 (each m, each 1H, $^{C}CH_2^{TMP}$), 1.04/0.96, 0.79/0.31 (each s, each 3H, CH_3^{TMP}).

^{13}C{^{1}H} NMR (151 MHz, 233 K, CD_2Cl_2): δ = 69.3 (CH), 55.2, 53.9 (NC^{TMP}), 47.9 (NCH_2), 40.3, 40.1 ($^{C}CH_2^{TMP}$), 34.1/19.7, 32.2/21.2, (CH_3^{TMP}), 17.1 (CH_2^{TMP}), 15.0 (CH_3), [C_6F_5 not listed].

^{11}B{^{1}H} NMR (192 MHz, 233 K, CD_2Cl_2): δ = 12.2 ($\nu_{1/2}$ ~ 580 Hz).

^{19}F NMR (564 MHz, 233 K, CD_2Cl_2): δ = -136.1 (m, 2F, o-C_6F_5), -156.1 (t, $^{3}J_{FF}$ = 20.4 Hz, 1F, p-C_6F_5), -163.5 (m, 2F, m-C_6F_5)[$\Delta\delta^{19}F_{m,p}$ = 7.4];

-136.2 (m, 2F, o-C_6F_5), -154.6 (t, $^3J_{FF}$ = 20.6 Hz, 1F, p-C_6F_5), -163.4 (m, 2F, m-C_6F_5)[$\Delta\delta^{19}F_{m,p}$ = 8.8].

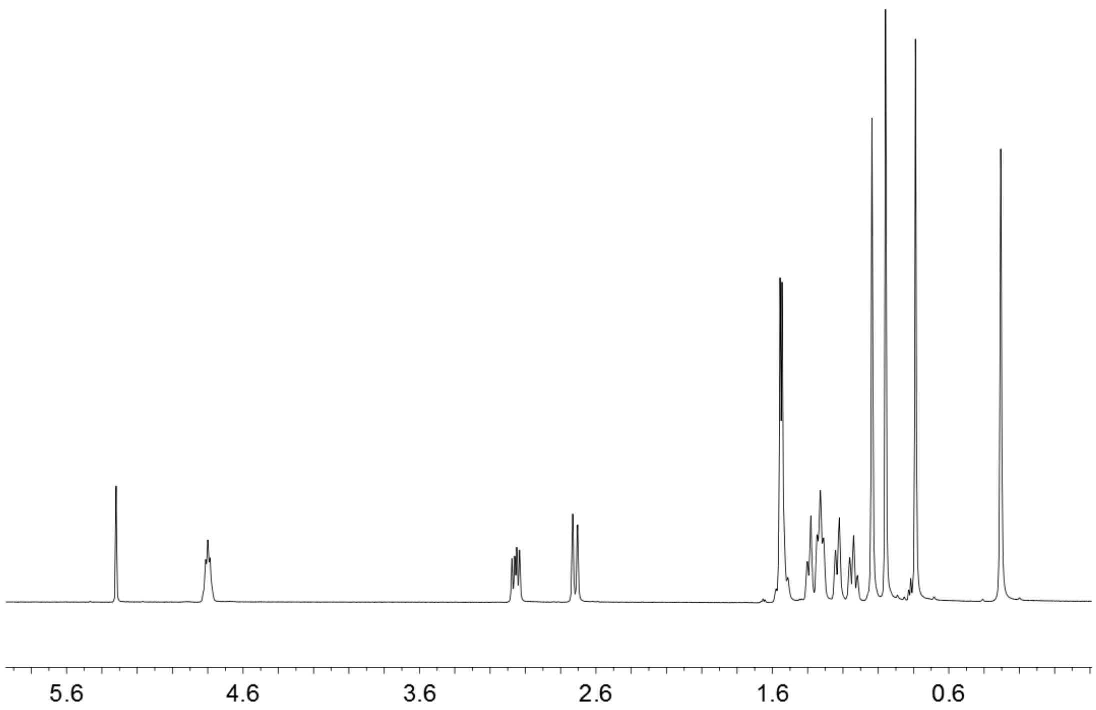

Figure S31. ^{1}H NMR (600 MHz, 233 K, CD_2Cl_2) spectrum of compound **17a**.

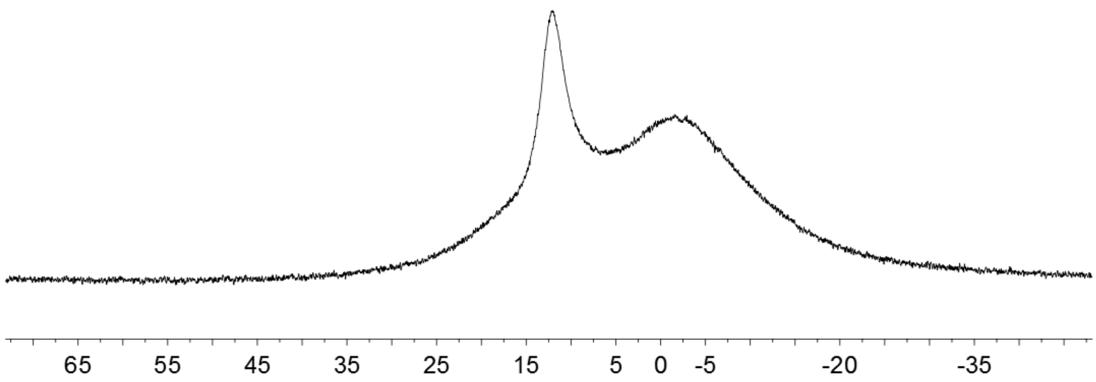

Figure S32. $^{11}B\{^1H\}$ NMR (192 MHz, 233 K, CD_2Cl_2) spectrum of compound **17a**.

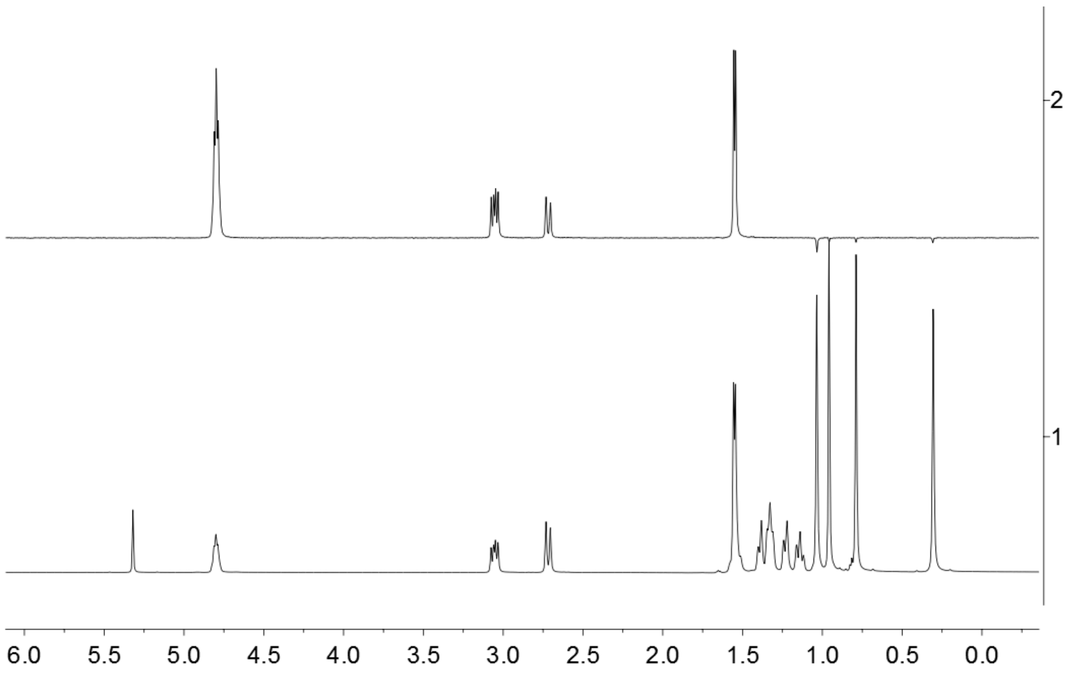

Figure S33. (1) ^{1}H NMR and (2) $^{1}H\{^{1}H\}$ TOCSY (600 MHz, 233 K, CD_2Cl_2) spectra of compound **17a**. * Irradiation point: $^{1}H_{irr}$ = 4.80 (CH).

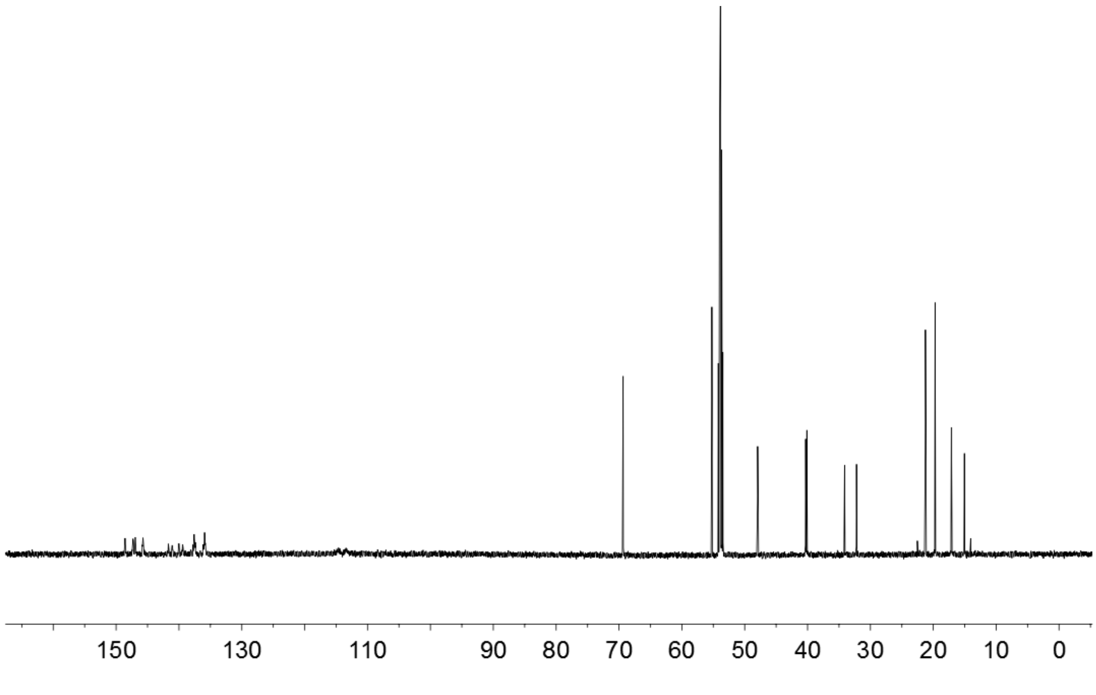

Figure S34. $^{13}C\{^{1}H\}$ NMR (151 MHz, 233 K, CD_2Cl_2) spectrum of compound **17a**.

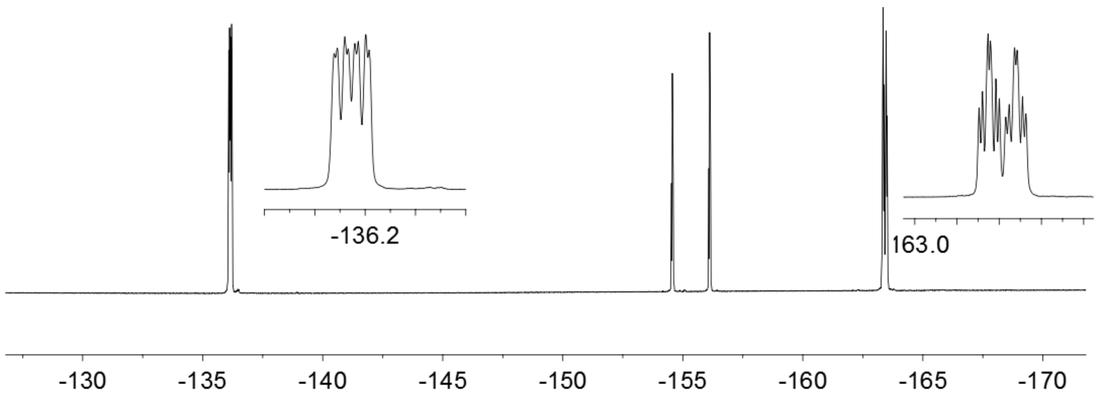

Figure S35. ^{19}F NMR (564 MHz, 233 K, CD_2Cl_2) spectrum of compound **17a**.

b) ^{1}H NMR spectra of compound 12a from 299 K to 233 K

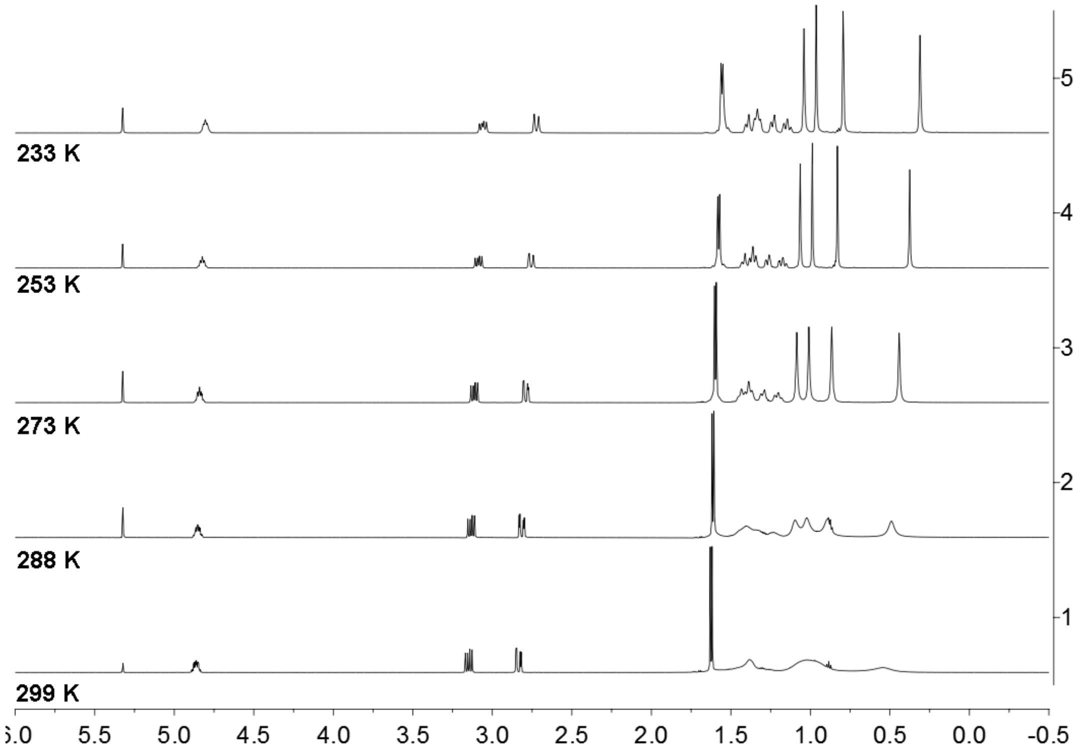

Figure S36. ^{1}H NMR (600 MHz, from 299 K to 233 K, CD_2Cl_2) spectra of compound **17a**.

Crystals suitable for the X-ray crystal structure analysis were obtained from a solution of compound **17a** in *n*-pentane at -35 °C.

X-ray crystal structure analysis of compound 17a: formula $C_{24}H_{24}BF_{10}N_3O_2$, M = 587.27, colourless crystal, 0.14 x 0.12 x 0.07 mm, a = 7.0432(1), b = 18.5654(4), c = 19.9939(4) Å, β = 98.022(1)°, V = 2588.8(1) Å^3, ρ_{calc} = 1.507 gcm^{-3}, μ = 0.145 mm^{-1}, empirical absorption correction (0.980 ≤ T ≤ 0.989), Z = 4, monoclinic, space group $P2_1/n$ (No. 14), λ = 0.71073Å, T = 223(2) K, ω and φ scans, 19379 reflections collected (±h, ±k, ±l), 4490 independent (R_{int} = 0.040) and 3585 observed reflections [I>2σ(I)], 366 refined parameters, R = 0.049, wR^2 = 0.122, max. (min.) residual electron density 0.25 (-0.16) e.Å^{-3}; hydrogen atoms were calculated and refined as riding atoms.

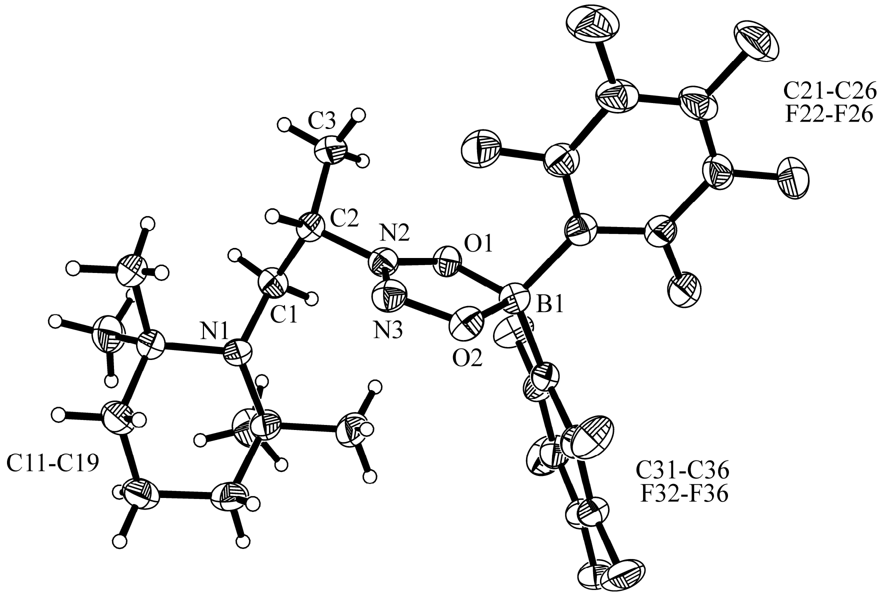

Figure S37. A view of the molecular structure of compound **17a**.

Synthesis of compound 22

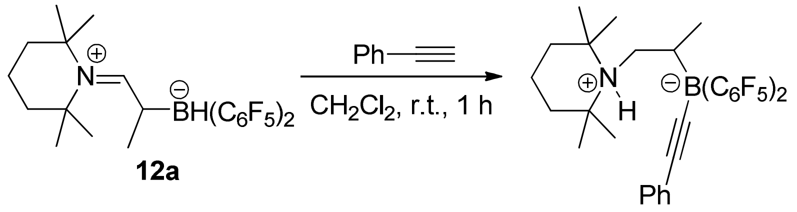

A solution of phenylacetylene (30.6 mg, 0.30 mmol) in CH_2Cl_2 (1 ml) was added to a solution of compound **12a** (105.5 mg, 0.20 mmol) in CH_2Cl_2 (3 ml). The mixture was stirred at room temperature for 1 h. Then all the volatiles were removed under reduced pressure. The obtained residue was washed with *n*-pentane (3×3 mL) and dried in vacuo to give a pale yellow solid. Yield: 114.6 mg, 0.18 mmol, 91%.

Anal. Calcd. for $C_{32}H_{30}F_{10}NB$: C, 61.07; H, 4.80; N, 2.23. Found: C, 60.50; H, 4.56; N, 2.11.

Mp (DSC): 117 °C

[TMP: 2,2,6,6-tetramethylpiperidino]

^{1}H NMR (600 MHz, 299 K, CD_2Cl_2): δ = 7.35 (m, 2H, o-Ph), 7.27 (m, 2H, m-Ph), 7.22 (m, 2H, p-Ph), 6.08 (br, 1H, NH), 3.71, 3.46 (each m, each 1H, NCH_2), 1.93 (br m, 1H, BCH), 1.74 (3H), 1.58 (1H) (each m, $^{C}CH_2^{TMP}$), 1.75/1.49 (each m, each 1H, CH_2^{TMP}), 1.61/1.38, 1.54/1.38 (each s, each 3H, CH_3^{TMP}), 0.93 (d, $^3J_{HH}$ = 6.8 Hz, 3H, CH_3).

$^{13}C\{^1H\}$ NMR (151 MHz, 299 K, CD_2Cl_2): δ = 131.5 (o-Ph), 128.7 (m-Ph), 127.1 (p-Ph), 126.5 (i-Ph), 111.9 (br, BC≡), 98.4 (br m, ≡C), 66.8, 66.7 (NC^{TMP}), 56.6 (NCH_2), 39.8, 39.7 (CH_2^{TMP}), 32.3/21.1, 31.1/21.3, (CH_3^{TMP}), 25.2 (br m, BCH), 16.19 (CH_2^{TMP}), 16.16 (m, CH_3), [C_6F_5 not listed].

$^{11}B\{^1H\}$ NMR (192 MHz, 299 K, CD_2Cl_2): δ = -17.3 ($\nu_{1/2}$ ~ 25 Hz).

^{19}F NMR (564 MHz, 299 K, CD_2Cl_2): δ = -130.0 (m, 2F, o-C_6F_5), -162.8 (t, $^3J_{FF}$ = 20.3 Hz, 1F, p-C_6F_5), -166.3 (m, 2F, m-C_6F_5)[$\Delta\delta^{19}F_{m,p}$ = 3.5]; -132.3 (m, 2F, o-C_6F_5), -163.0 (t, $^3J_{FF}$ = 20.3 Hz, 1F, p-C_6F_5), -166.2 (m, 2F, m-C_6F_5)[$\Delta\delta^{19}F_{m,p}$ = 3.2].

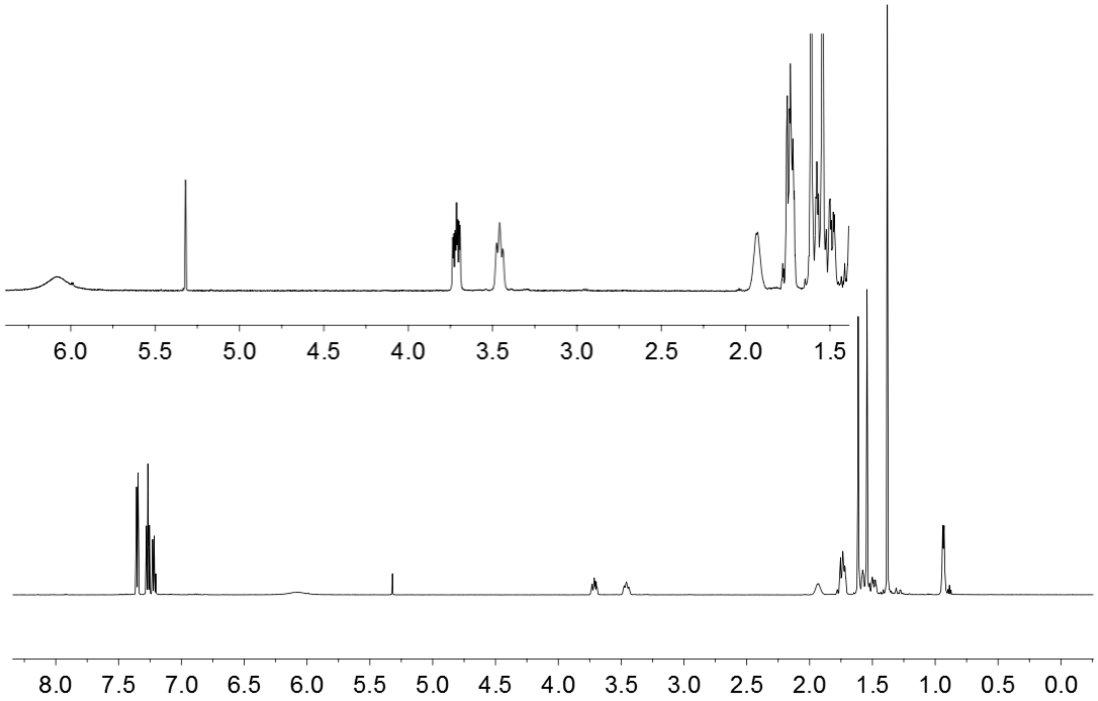

Figure S38. ^1H NMR (600 MHz, 299 K, CD_2Cl_2) spectrum of compound **22**.

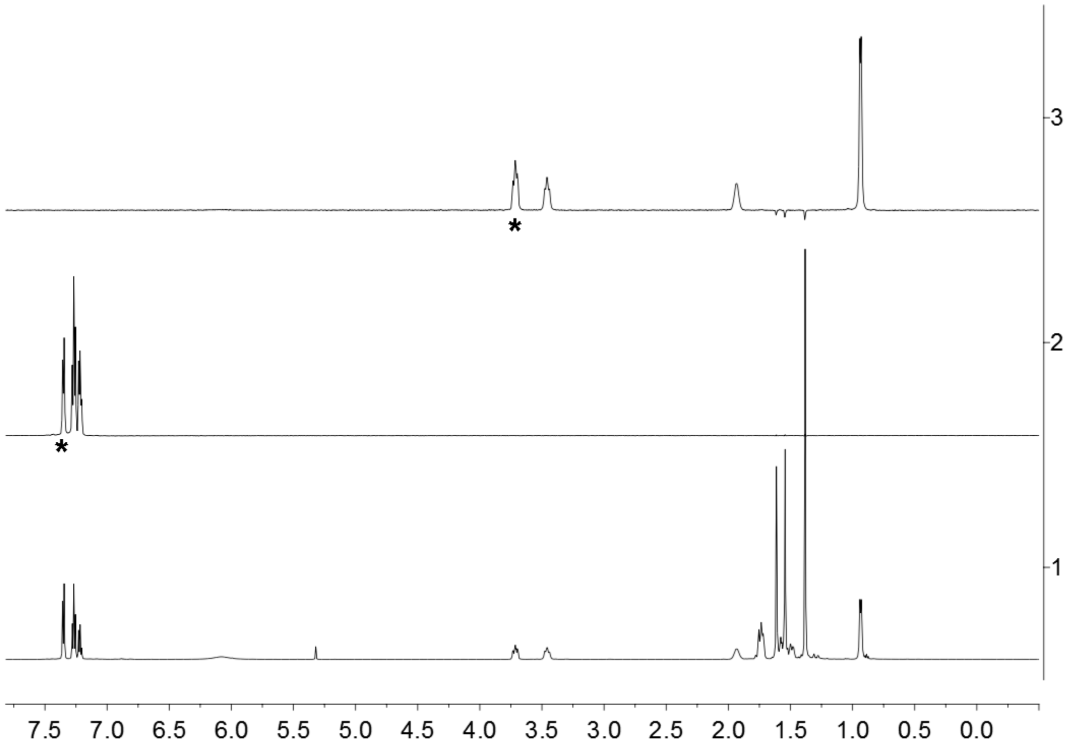

Figure S39. (1) ^{1}H NMR and (2,3) ^{1}H{^{1}H} TOCSY (600 MHz, 299 K, CD_2Cl_2) spectra of compound **22**. * Irradiation points: (2) $^{1}H_{irr}$ = 7.35 (o-Ph), (3) 3.71 (NCH_2).

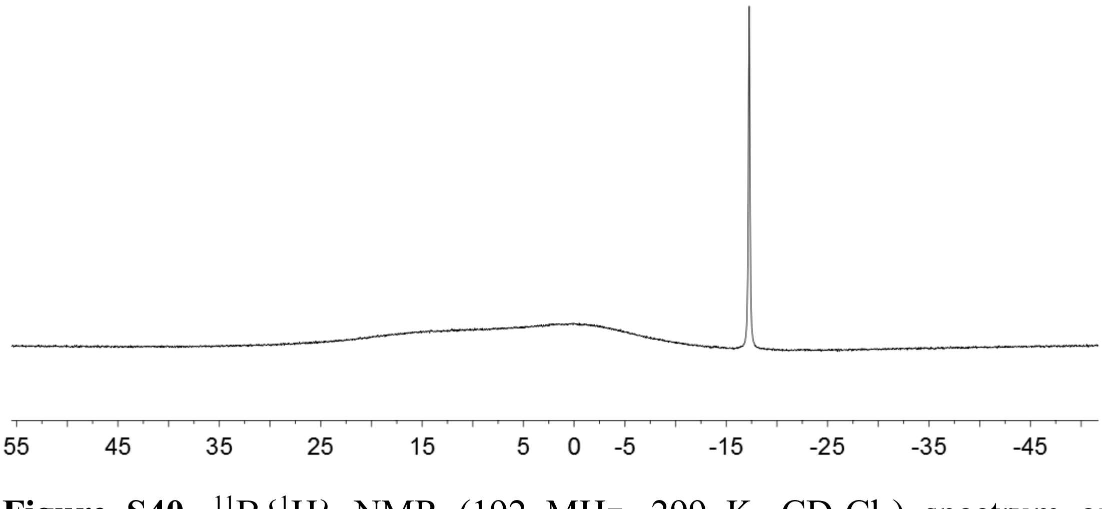

Figure S40. ^{11}B{^{1}H} NMR (192 MHz, 299 K, CD_2Cl_2) spectrum of compound **22**.

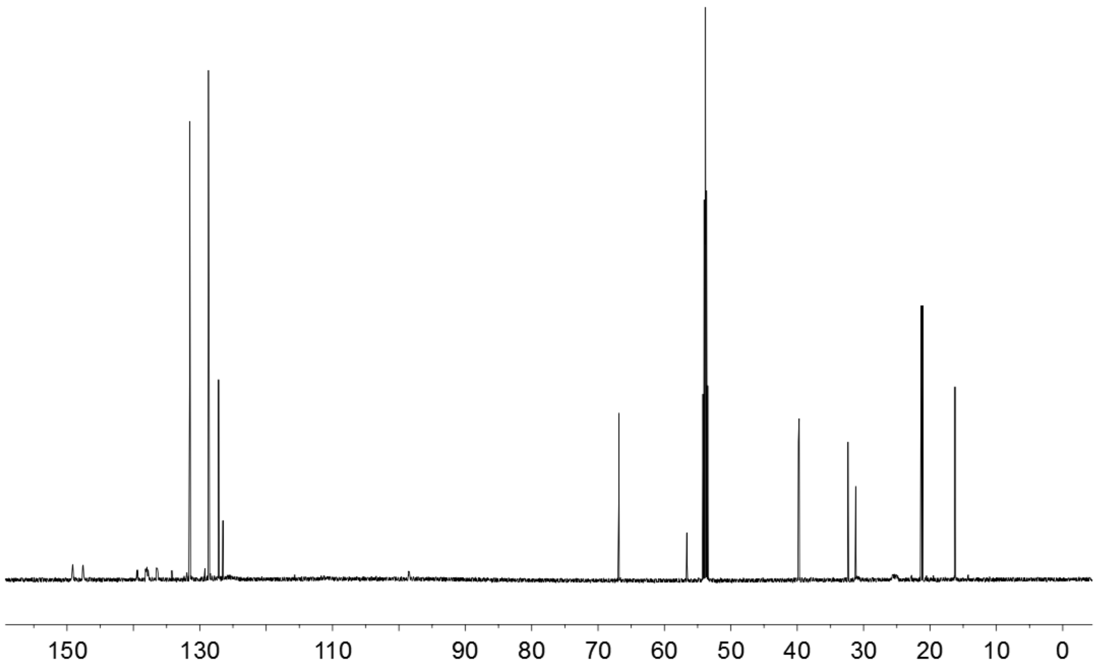

Figure S41. $^{13}C\{^1H\}$ NMR (151 MHz, 299 K, CD_2Cl_2) spectrum of compound **22**.

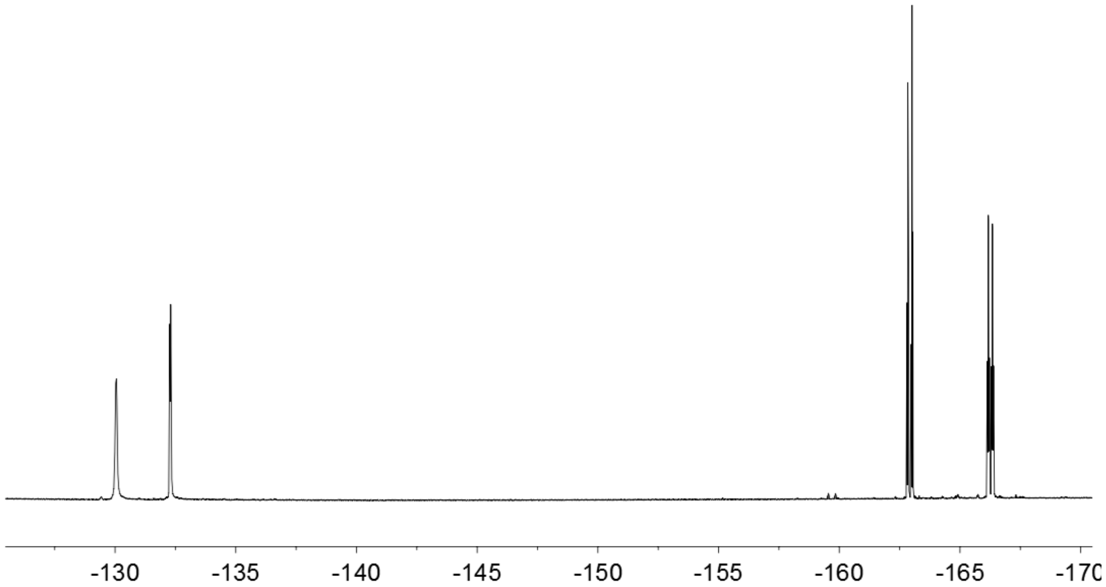

Figure S42. ^{19}F NMR (564 MHz, 299 K, CD_2Cl_2) spectrum of compound **22**.

Crystals suitable for the X-ray crystal structure analysis were obtained from a two-layer procedure using a solution of compound **22** in CH_2Cl_2 covered with *n*-pentane at -35 °C.

X-ray crystal structure analysis of compound 22: formula $C_{32}H_{30}BF_{10}N$, M = 629.38, colourless crystal, 0.13 x 0.12 x 0.05 mm, a = 10.9697(2), b = 11.2538(2), c = 13.9662(4) Å, α = 93.564(1), β = 101.334(1), γ = 106.008(1)°, V = 1612.5(1) Å^3, ρ_{calc} = 1.296 gcm^{-3}, μ = 0.115 mm^{-1}, empirical absorption correction (0.985 ≤ T ≤ 0.994), Z = 2, triclinic, space group $P\bar{1}$ (No. 2), λ = 0.71073 Å, T = 223(2) K, ω and φ scans, 16999 reflections collected (±*h*, ±*k*, ±*l*), 7754 independent (R_{int} = 0.040) and 5594 observed reflections [$I>2\sigma(I)$], 406 refined parameters, R = 0.063 wR^2 = 0.150, max. (min.) residual electron density 0.24 (-0.20) e.Å^{-3}, the hydrogen position at N1 was refined freely, others hydrogens were calculated and refined as riding atoms.

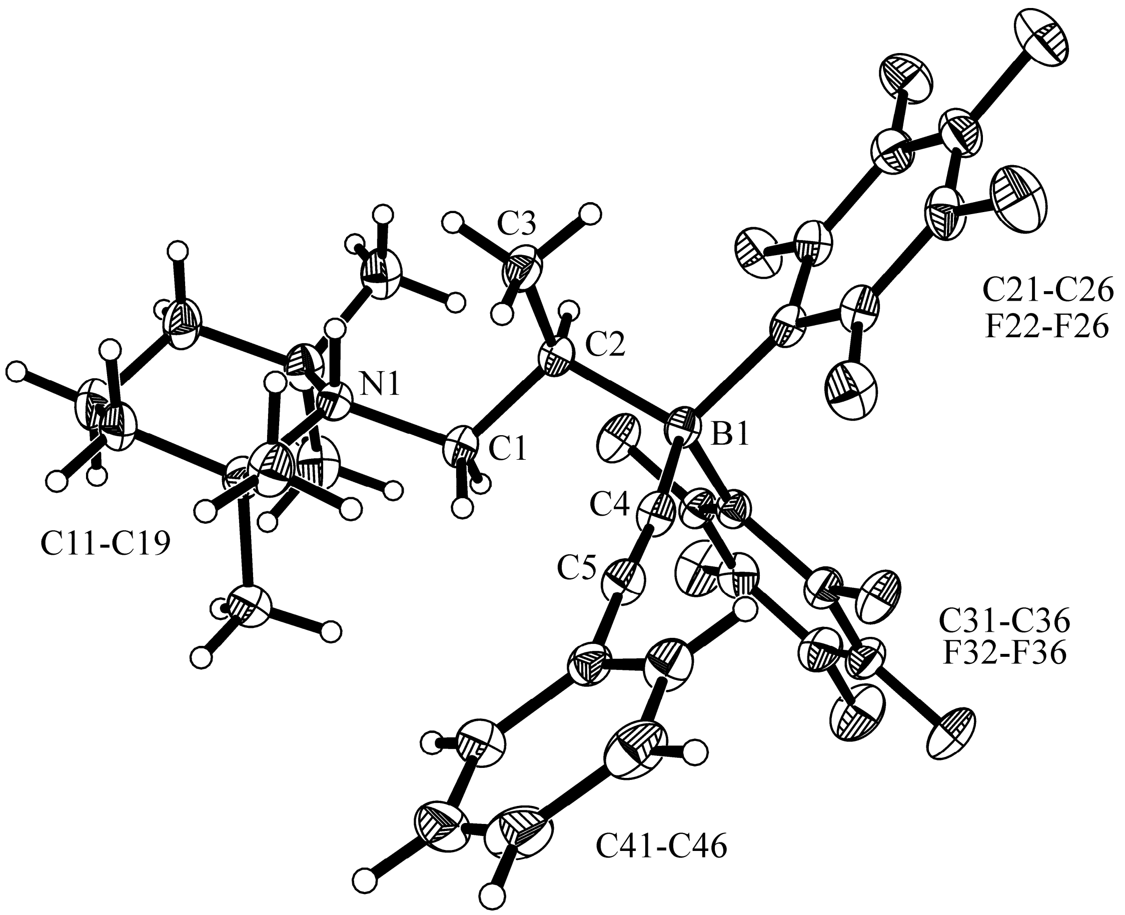

Figure S43. A view of the molecular structure of compound **22**.

Synthesis of compound 8b

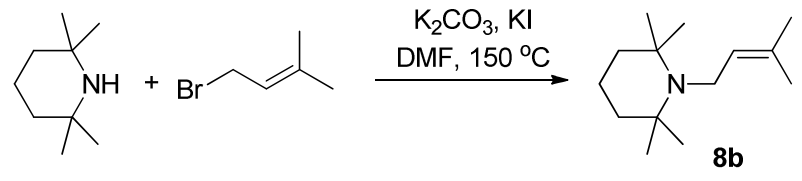

K_2CO_3 (10.20 g, 73.8 mmol, 2.5 equiv) and KI (5.40 g, 32.6 mmol, 1.1 equiv) were weighed together in a Schlenk flask. The flask was purged by repetitions of vacuum and argon filling for three times. Then DMF (80 mL), 2,2,6,6-tetramethylpiperidine (TMPH) (5.0 mL, 29.6 mmol, 1.0

equiv) and 3,3-dimethylallyl bromide (7.5 mL, 65.2 mmol, 2.2 equiv) was added to the mixture, respectively, and heated at 150 °C for 16 h. Then it was cooled to r.t. and the formed solid was removed by filtration. The filtrate was added to diethyl ether (100 mL), and washed with water (5×20 mL). The aqueous phase was collected, extracted with diethyl ether (100 mL), and the ether phases washed with water (5×20 mL). The combined organic solutions were dried with $MgSO_4$, filtered, concentrated and purified by silica gel column chromatography (eluent: *n*-pentane: ethyl acetate: triethylamine = 100:5:1). The product was dried in vacuo and obtained as a yellow oil. Yield: 4.35 g, 20.8 mmol, 70%.

Anal. Calcd. for $C_{14}H_{27}N$: C, 80.31; H, 13.00; N, 6.69. Found: C, 80.33; H, 13.29; N, 6.62.

[TMP: 2,2,6,6-tetramethylpiperidino]

^{1}H NMR (600 MHz, 299 K, $CDCl_3$): δ = 5.15 (m, 1H, CH=), 3.05 (dm, $^3J_{HH}$ = 5.7 Hz, 2H, NCH_2), 1.65^E, 1.60^Z (each m, each 3H, CH_3), 1.53 (m, 2H, CH_2^{TMP}), 1.41 (m, 4H, $^CCH_2^{TMP}$), 1.01 (s, 12H, CH_3^{TMP}).

^{13}C{^{1}H} NMR (151 MHz, 299 K, $CDCl_3$): δ = 130.9 (CH=), 127.1 (=C), 54.6 (NC^{TMP}), 42.2 (NCH_2), 41.3 ($^CCH_2^{TMP}$), 27.4 (br, CH_3^{TMP}), 25.7^E, 17.9^Z (CH_3), 17.8 (CH_2^{TMP}).

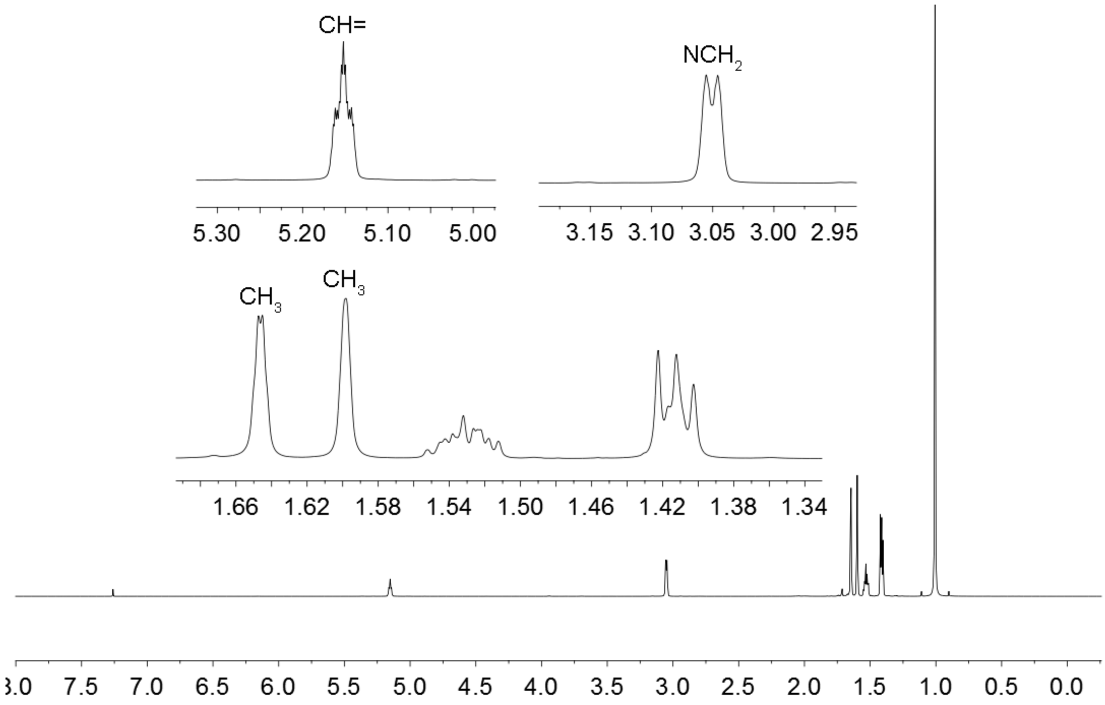

Figure S44. ^{1}H NMR (600 MHz, 299 K, $CDCl_3$) spectrum of compound **8b**.

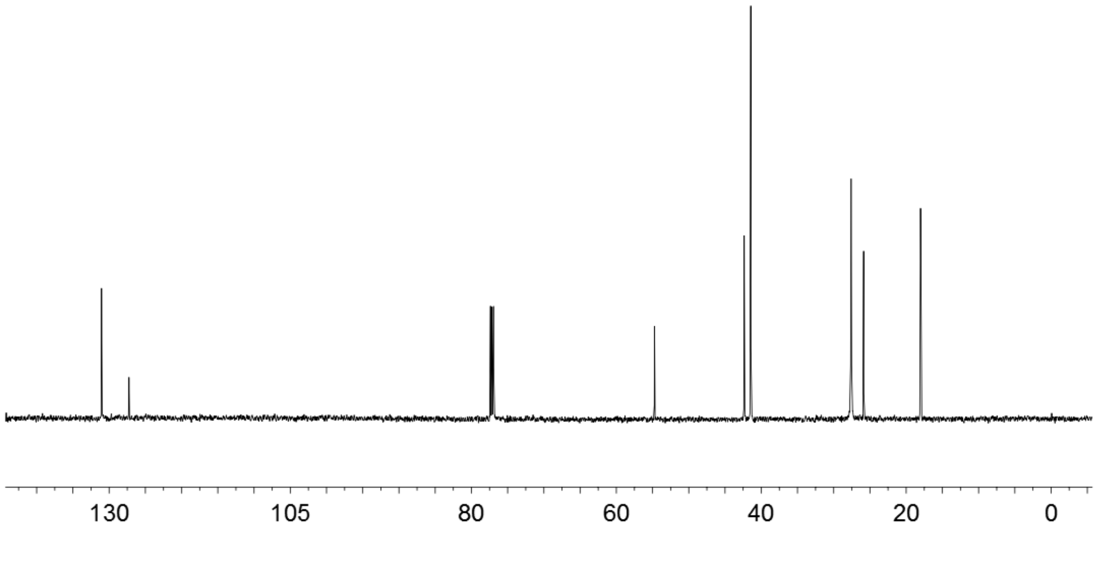

Figure S45. $^{13}C\{^{1}H\}$ NMR (151 MHz, 299 K, $CDCl_3$) spectrum of compound **8b**.

Synthesis of compound 12b

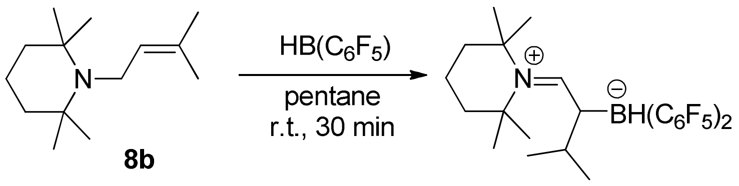

A solution of compound **8b** (209.5 mg, 1.00 mmol) in *n*-pentane (2 mL) was added to a suspension of $HB(C_6F_5)_2$ (346.0 mg, 1.00 mmol) in *n*-pentane (20 mL), which resulted in a yellow solution after 10 min. The mixture was stirred at room temperature for 30 min to give a white precipitate. The solid was collected by filtration and dried in vacuo to give a white solid. Yield: 494.3 mg, 0.89 mmol, 89%.

Anal. Calcd. for $C_{26}H_{28}F_{10}NB$: C, 56.24; H, 5.08; N, 2.52. Found: C, 56.28; H, 5.33; N, 2.60.

Mp (DSC): 88°C

[TMP: 2,2,6,6-tetramethylpiperidino]

^{1}H NMR (600 MHz, 299 K, CD_2Cl_2): δ = 8.40 (d, $^3J_{HH}$ = 13.4 Hz, 1H, N=CH), 3.35 (m, 1H, BCH), 2.84 (br 1:1:1:1 q[a], $^1J_{BH}$ ~ 95 Hz, 1H, BH), 2.06 (m, 1H, CH^{iPr}), 1.95/1.81 (each 1H), 1.88 (2H) (each m, $^{C}CH_2^{TMP}$), 1.79 (br m, 2H, CH_2^{TMP}), 1.67, 1.57, 1.55, 1.23 (each s, each 3H, CH_3^{TMP}), 1.00 (d, $^3J_{HH}$ = 6.8 Hz, 3H, CH_3^{iPr}), 0.89 (d, $^3J_{HH}$ = 6.9 Hz, 3H, CH_3^{iPr}), [[a] partially relaxed].

^{13}C{^{1}H} NMR (151 MHz, 299 K, CD_2Cl_2): δ = 187.1 (N=CH), 148.6 (dm,

$^1J_{FC}$ ~ 235 Hz, C_6F_5), 138.8 (dm, $^1J_{FC}$ ~ 245 Hz, C_6F_5), 137.2 (dm, $^1J_{FC}$ ~ 240 Hz, C_6F_5), 123.5 (i-C_6F_5), 68.4, 64.5 (br, NC^{TMP}), 48.9 (br m, BCH), 38.3, 35.3 (each br, $^{C}CH_2^{TMP}$), 33.4 (CH^{iPr}), 32.6, 30.3, 30.0, 29.9 (each br, CH_3^{TMP}), 25.0, 22.3 (CH_3^{iPr}), 14.4 (CH_2^{TMP}).

$^{11}B\{^1H\}$ NMR (192 MHz, 299 K, CD_2Cl_2): δ = -21.1 ($\nu_{1/2}$ ~ 45 Hz).

^{11}B NMR (192 MHz, 299 K, CD_2Cl_2): δ = -21.1 (d, $^1J_{HB}$ ~ 92 Hz).

^{19}F NMR (564 MHz, 299 K, CD_2Cl_2): δ = -131.3 (m, 2F, o-C_6F_5), -162.1 (t, $^3J_{FF}$ = 19.5 Hz, 1F, p-C_6F_5), -166.0 (m, 2F, m-C_6F_5)[$\Delta\delta^{19}F_{m,p}$ = 3.9].

^{19}F NMR (470 MHz, 243 K, CD_2Cl_2): δ = -131.3 (m, 2F, o-C_6F_5), -161.5 (t, $^3J_{FF}$ = 20.7 Hz, 1F, p-C_6F_5), -165.63 (m, 2F, m-C_6F_5)[$\Delta\delta^{19}F_{m,p}$ = 4.1]; -131.6 (m, 2F, o-C_6F_5), -161.7 (t, $^3J_{FF}$ = 20.6 Hz, 1F, p-C_6F_5), -165.57 (m, 2F, m-C_6F_5)[$\Delta\delta^{19}F_{m,p}$ = 3.9].

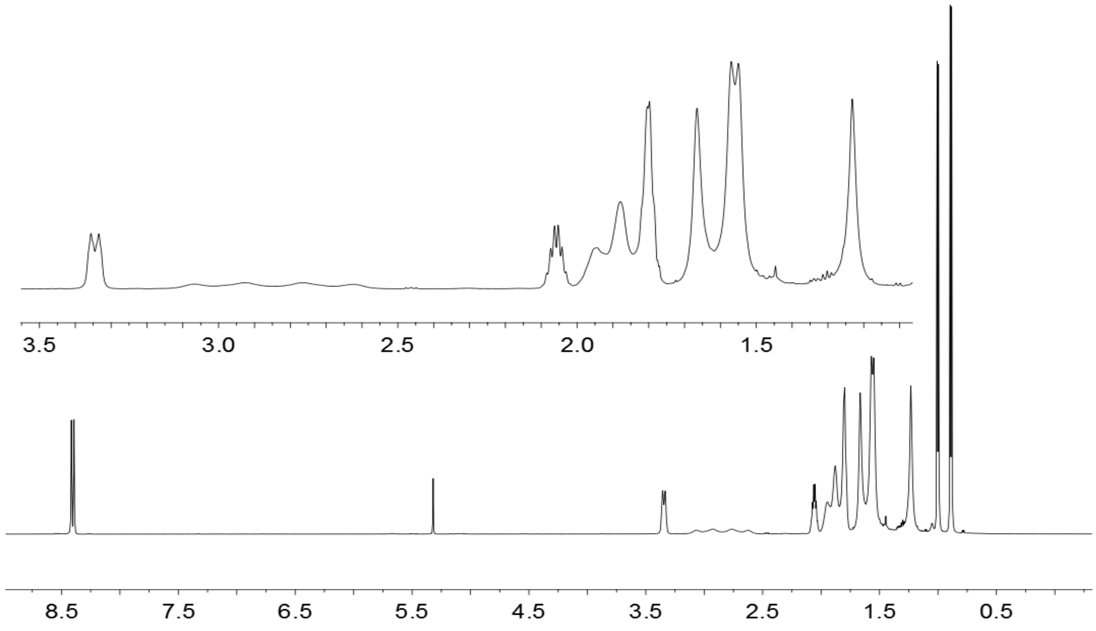

Figure S46. ^1H NMR (600 MHz, 299 K, CD_2Cl_2) spectrum of compound **12b**.

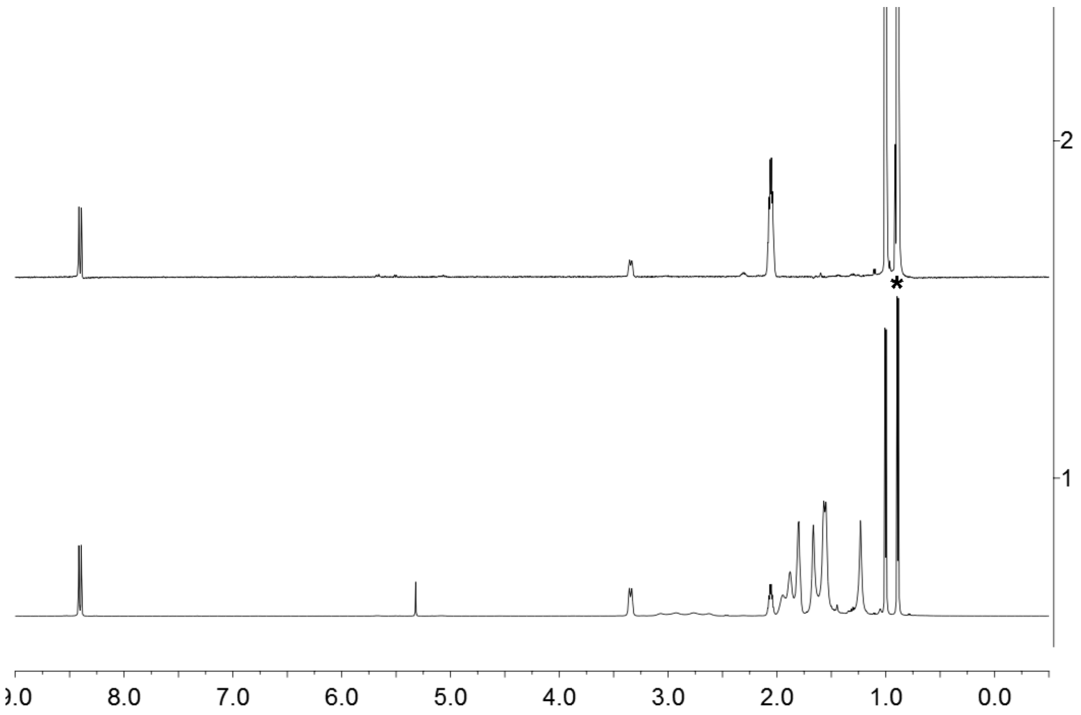

Figure S47. (1) ^{1}H NMR and (2) $^{1}H\{^{1}H\}$ TOCSY (600 MHz, 299 K, CD_2Cl_2) spectra of compound **12b**. * Irradiation point: $\delta^{1}H_{irr}$ = 0.89 (CH_3^{iPr}).

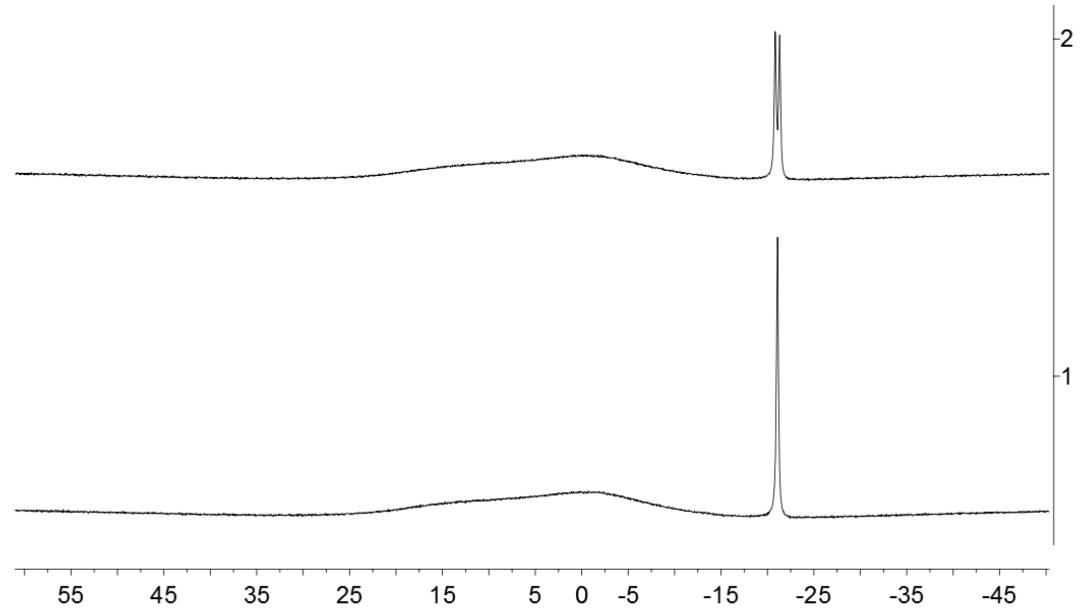

Figure S48. $^{11}B\{^{1}H\}$ NMR (192 MHz, 299 K, CD_2Cl_2) spectrum of compound **12b**.

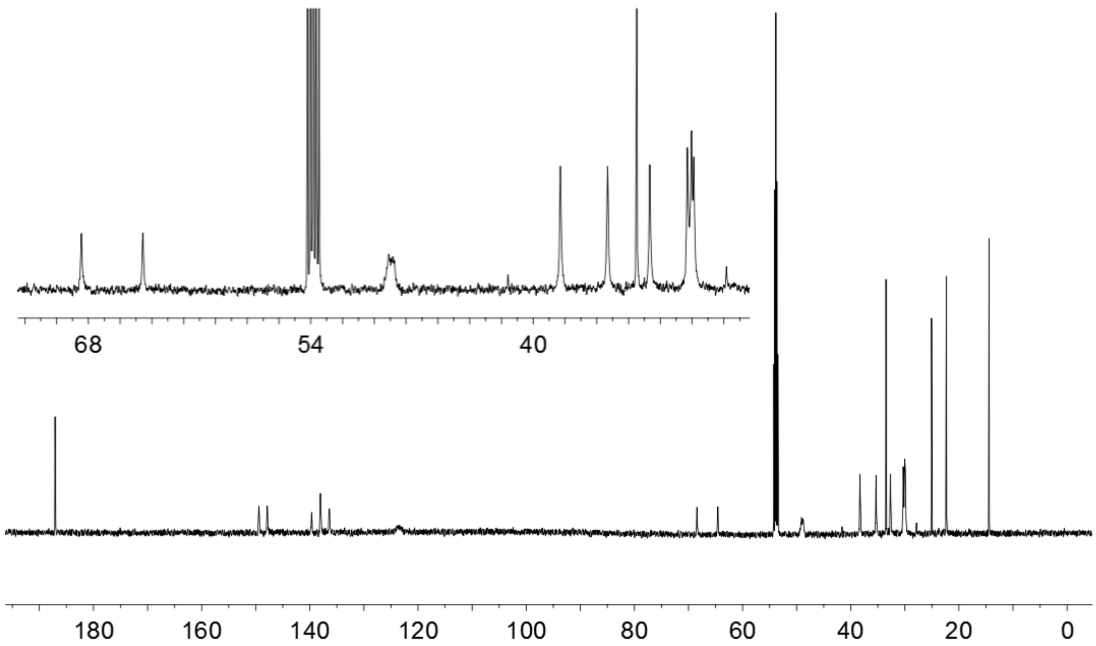

Figure S49. $^{13}C\{^{1}H\}$ NMR (151 MHz, 299 K, CD_2Cl_2) spectrum of compound **12b**.

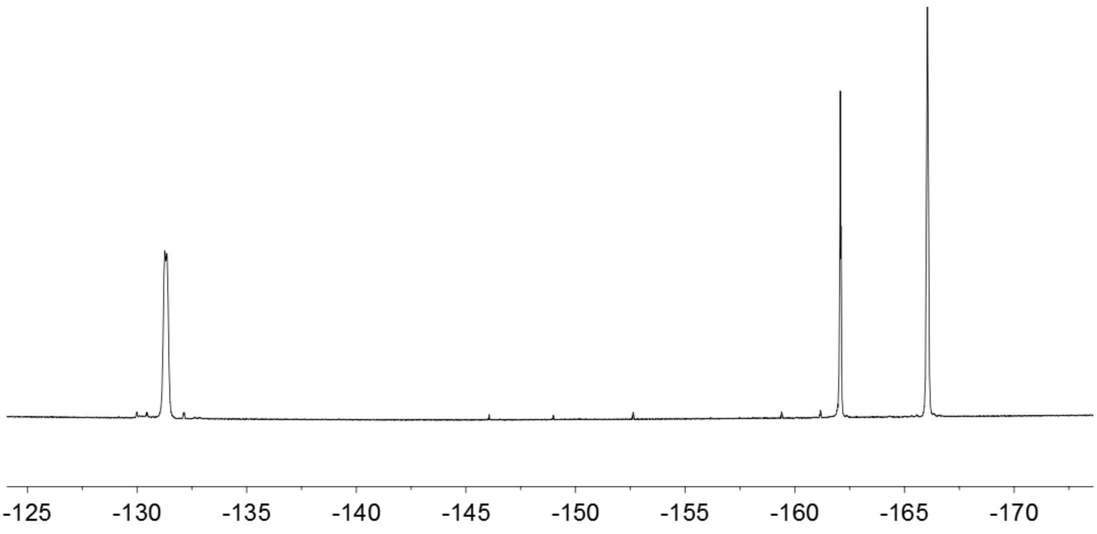

Figure S50. ^{19}F NMR (564 MHz, 299 K, CD_2Cl_2) spectrum of compound **12b**.

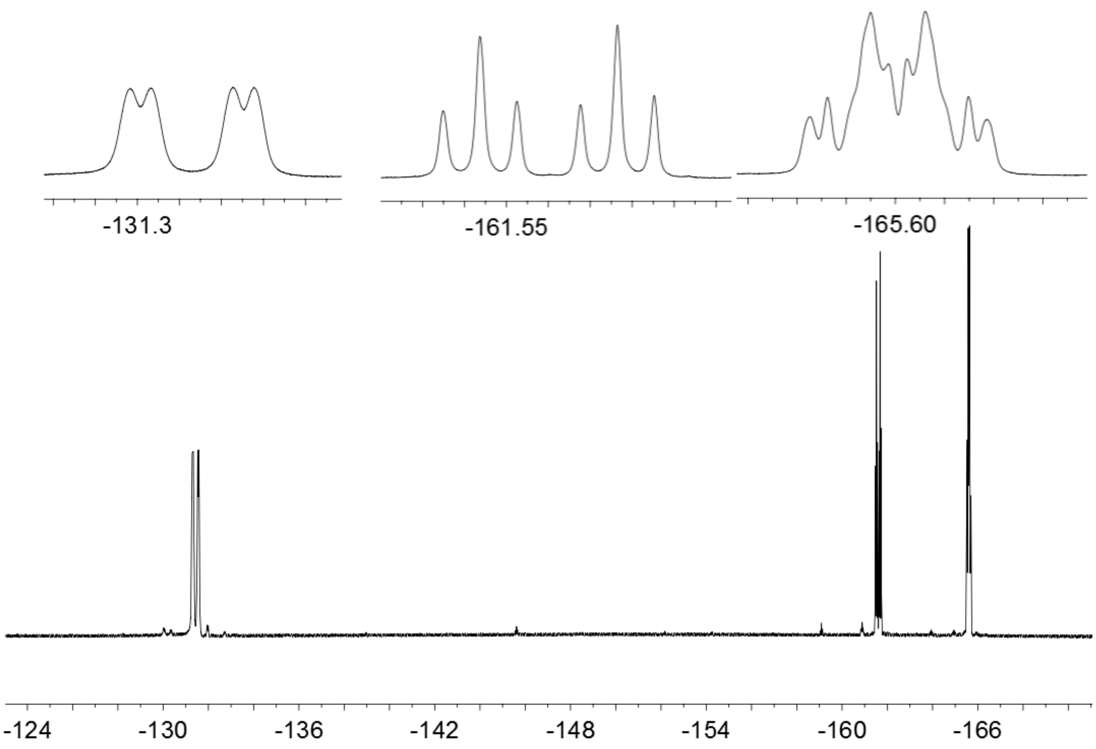

Figure S51. ^{19}F NMR (470 MHz, 243 K, CD_2Cl_2) spectrum of compound **12b**.

Synthesis of compound 12b-D starting from $DB(C_6F_5)_2$

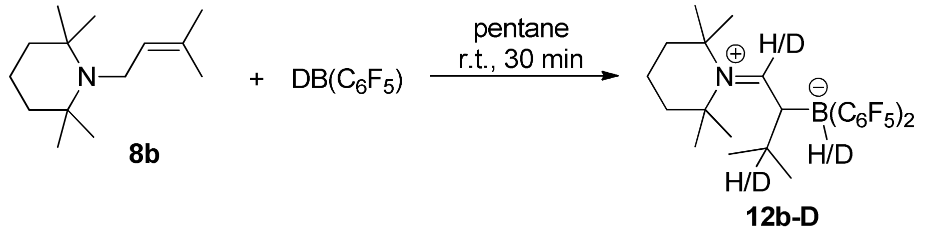

A solution of compound **8b** (41.9 mg, 0.20 mmol) in *n*-pentane (1 mL) was added to a suspension of $DB(C_6F_5)_2$ (69.3 mg, 0.20 mmol) in *n*-pentane (3 mL). The mixture was stirred at room temperature for 30 min to give a white precipitate. The precipitate was collected by filtration

and dried in vacuo to give compound **12b-D** as a white solid. Yield: 86.7 mg, 0.16 mmol, 78%.

^{2}H NMR (92 MHz, 299 K, CH_2Cl_2): δ = 8.41 (br, N=CD), 2.90 (br, BD), 2.04 (m, CD^{iPr}).

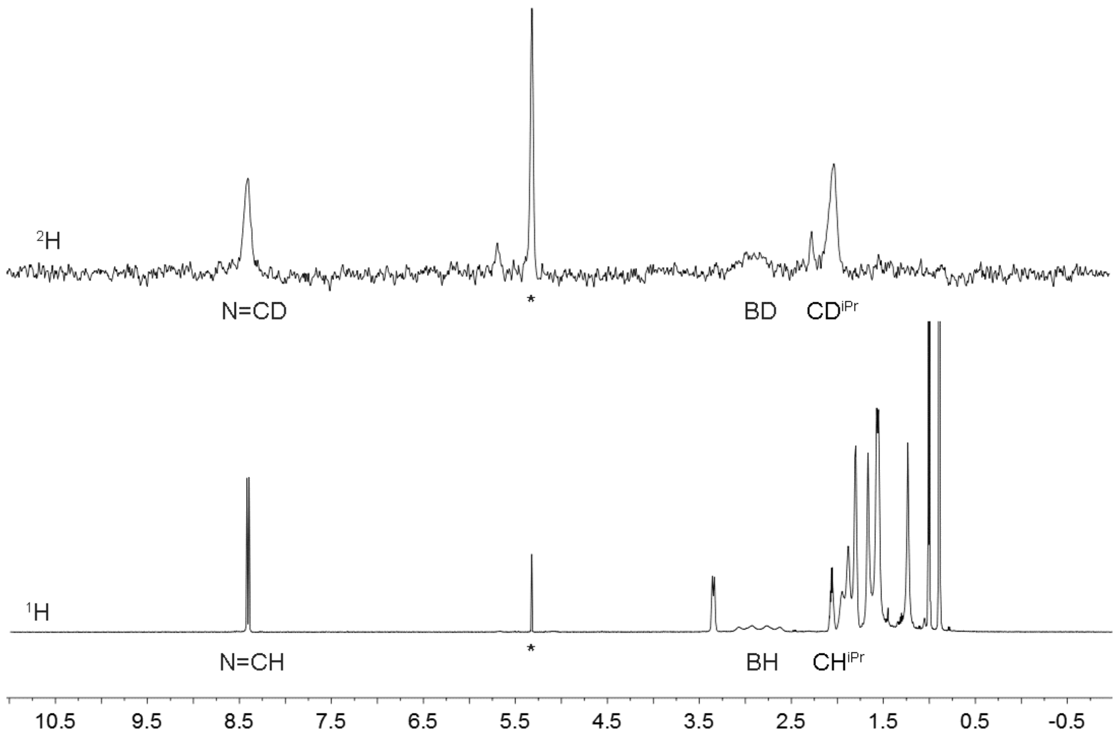

Figure S52. (top) ^{2}H NMR (92 MHz, 299 K, CH_2Cl_2) spectrum of compound **12b-D** and (bottom) ^{1}H NMR (600 MHz, 299 K, CD_2Cl_2) spectrum of compound **12b**. [* $CDHCl_2$]

Synthesis of compound 17b

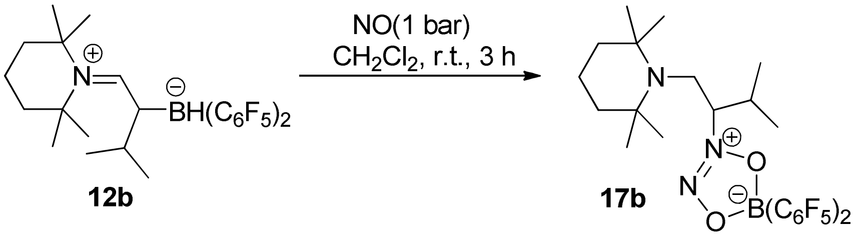

Compound **12b** (180.7 mg, 0.33 mmol) was dissolved in CH_2Cl_2 (3 mL) to give a colorless solution. The solution was degassed by applying vacuum carefully at r.t.. Then NO (1.0 bar) was pressed over the solution, and the mixture was stirred at room temperature for 3 h. Then all volatiles were removed under reduced pressure and the obtained residue was extracted with *n*-pentane (3×30 mL). The solvent was removed from the combined pentane phases in vacuo to give a pale yellow solid. Yield: 138.1 mg, 0.22 mmol, 69%.

Anal. Calcd. for $C_{26}H_{28}F_{10}N_3O_2B$: C, 50.75; H, 4.59; N, 6.83. Found: C, 50.72; H, 4.48; N, 6.75.

Mp (DSC): 115 °C

a) NMR data of compound 17b at 233 K

[TMP: 2,2,6,6-tetramethylpiperidino]

^{1}H NMR (600 MHz, 233 K, CD_2Cl_2): δ = 4.44 (m, CH), 3.08 (dd, $^2J_{HH}$ = 16.3 Hz, $^3J_{HH}$ = 9.8 Hz) and 2.95 (d, $^2J_{HH}$ = 16.3 Hz) (each 1H, NCH_2), 2.32 (m, 1H, CH^{iPr}), 1.54/1.31 (each m, each 1H, CH_2^{TMP}), 1.37/1.33, 1.22/1.13 (each m, each 1H, $^{C}CH_2^{TMP}$), 1.04/0.98, 0.81/0.33 (each s, each 3H, CH_3^{TMP}), 1.07 (d, $^3J_{HH}$ = 6.7 Hz, 3H, CH_3^{iPr}), 0.94 (d, $^3J_{HH}$ = 6.6 Hz,

3H, CH_3^{iPr}).

$^{13}C\{^1H\}$ NMR (151 MHz, 233 K, CD_2Cl_2): δ = 78.9 (CH), 55.4, 54.0 (NC^{TMP}), 44.2 (NCH_2), 40.5, 40.2 ($^{C}CH_2^{TMP}$), 34.3/19.7, 32.3/21.3 (CH_3^{TMP}), 29.3 (CH^{iPr}), 19.0, 18.3 (CH_3^{iPr}), 17.1 (CH_2^{TMP}) [C_6F_5 not listed].

$^{11}B\{^1H\}$ NMR (192 MHz, 233 K, CD_2Cl_2): δ = 12.6 ($\nu_{1/2}$ ~ 650 Hz).

^{19}F NMR (564 MHz, 233 K, CD_2Cl_2): δ = -135.5 (m, 2F, o-C_6F_5), -156.1 (t, $^3J_{FF}$ = 20.6 Hz, 1F, p-C_6F_5), -163.4 (m, 2F, m-C_6F_5)[$\Delta\delta^{19}F_{m,p}$ = 7.3]; -136.0 (m, 2F, o-C_6F_5), -155.8 (t, $^3J_{FF}$ = 20.6 Hz, 1F, p-C_6F_5), -163.3 (m, 2F, m-C_6F_5)[$\Delta\delta^{19}F_{m,p}$ = 7.5].

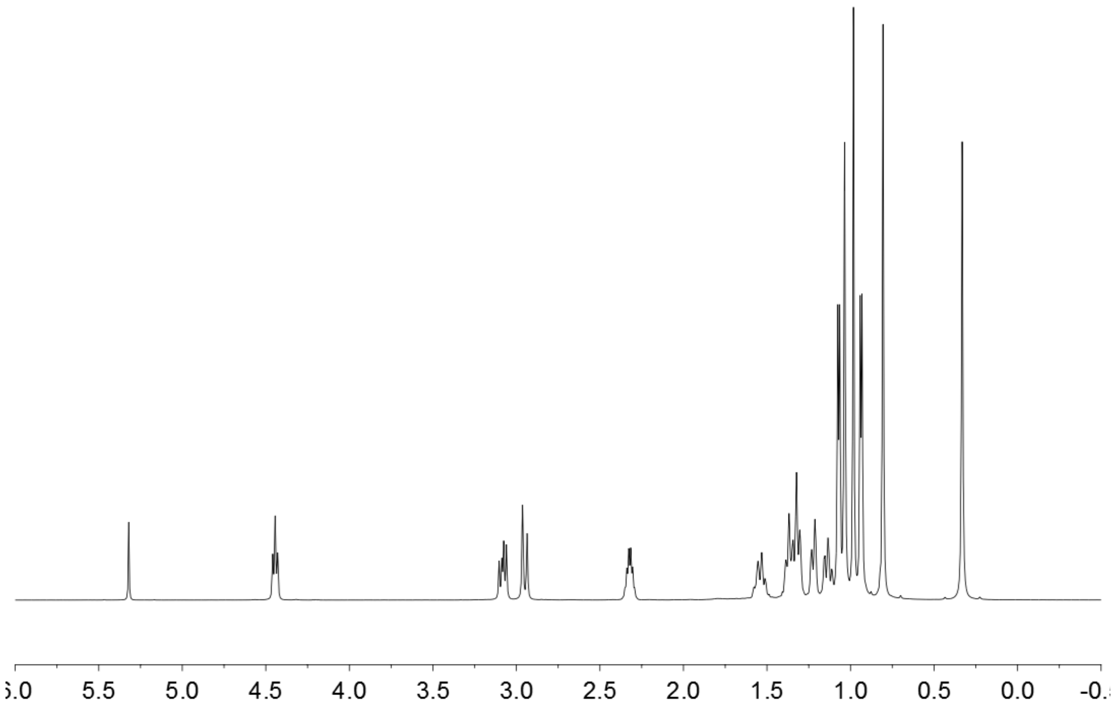

Figure S53. ^1H NMR (600 MHz, 233 K, CD_2Cl_2) spectrum of compound **17b**.

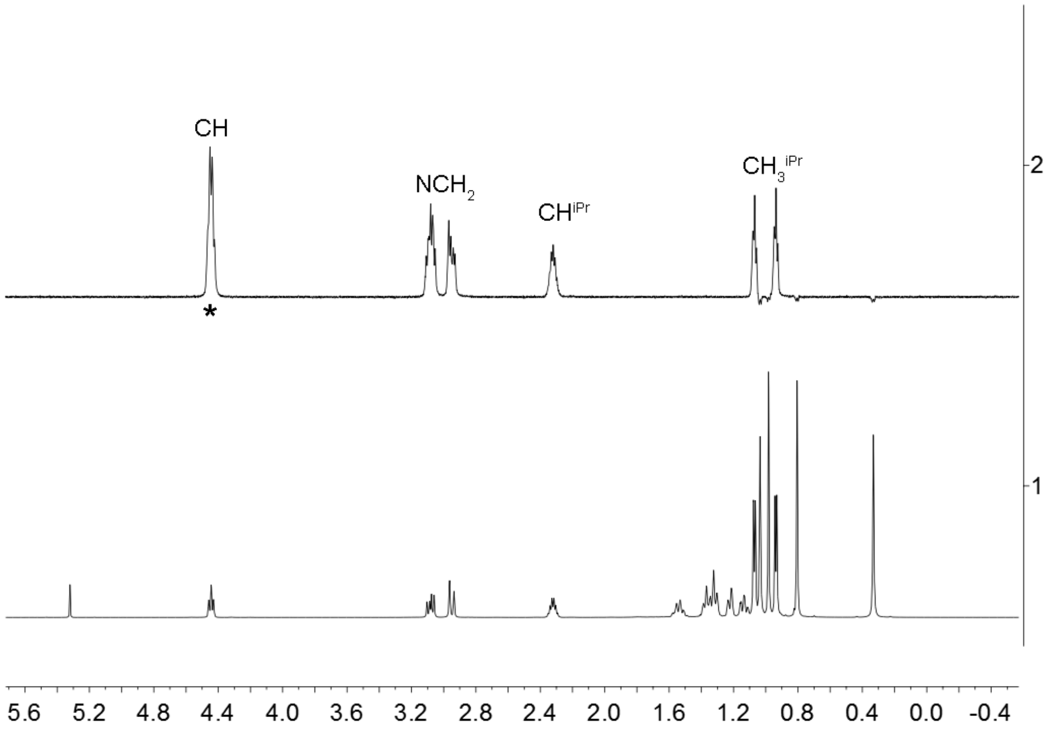

Figure S54. (1) ^{1}H NMR and (2) $^{1}H\{^{1}H\}$ TOCSY (600 MHz, 233 K, CD_2Cl_2) spectra of compound **17b**. * Irradiation point: $^{1}H_{irr}$ = 4.44 (CH).

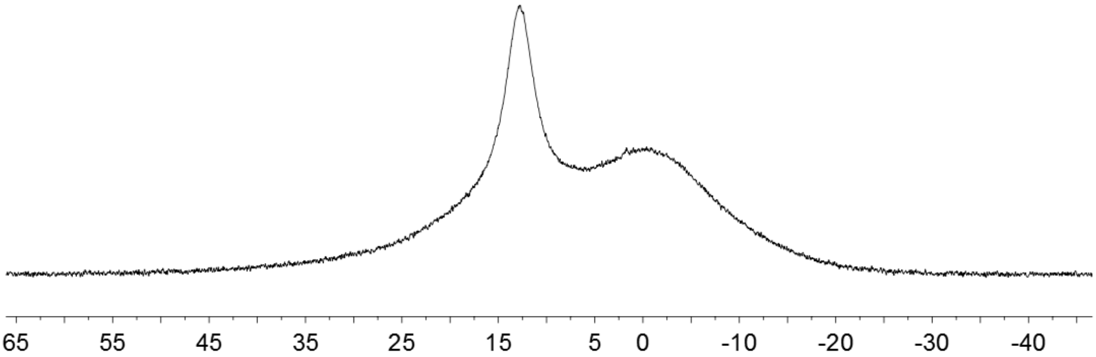

Figure S55. $^{11}B\{^{1}H\}$ NMR (192 MHz, 233 K, CD_2Cl_2) spectrum of compound **17b**.

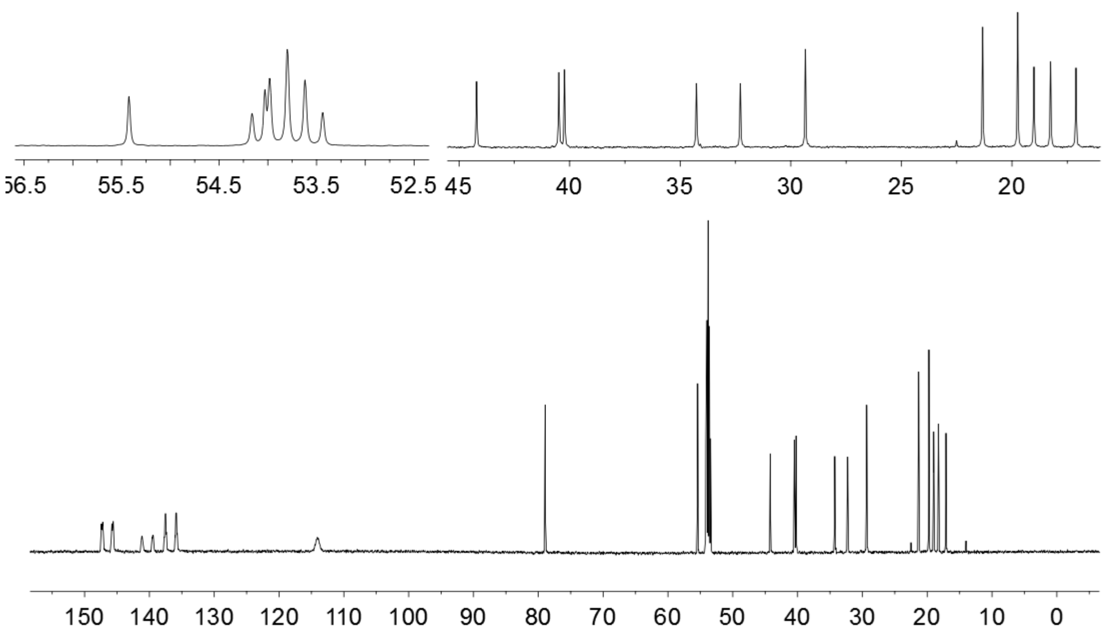

Figure S56. $^{13}C\{^{1}H\}$ NMR (151 MHz, 233 K, CD_2Cl_2) spectrum of compound **17b**.

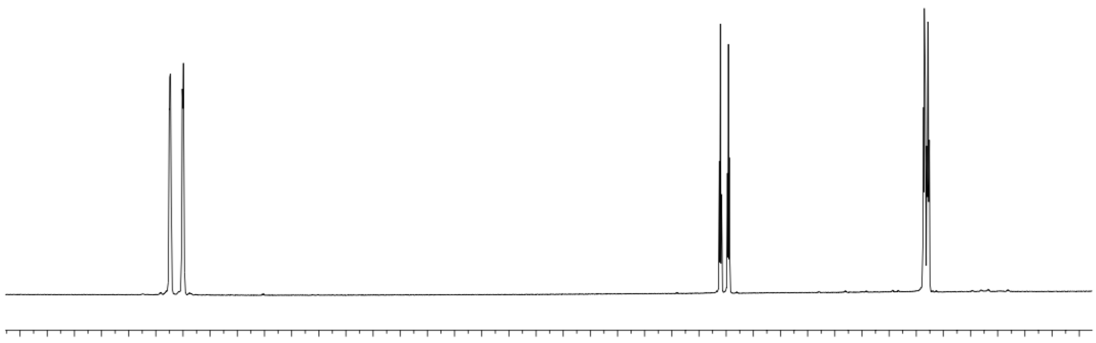

Figure S57. ^{19}F NMR (564 MHz, 233 K, CD_2Cl_2) spectrum of compound **17b**.

b) ^{1}H NMR spectra of compound 17b from 299 K to 233 K

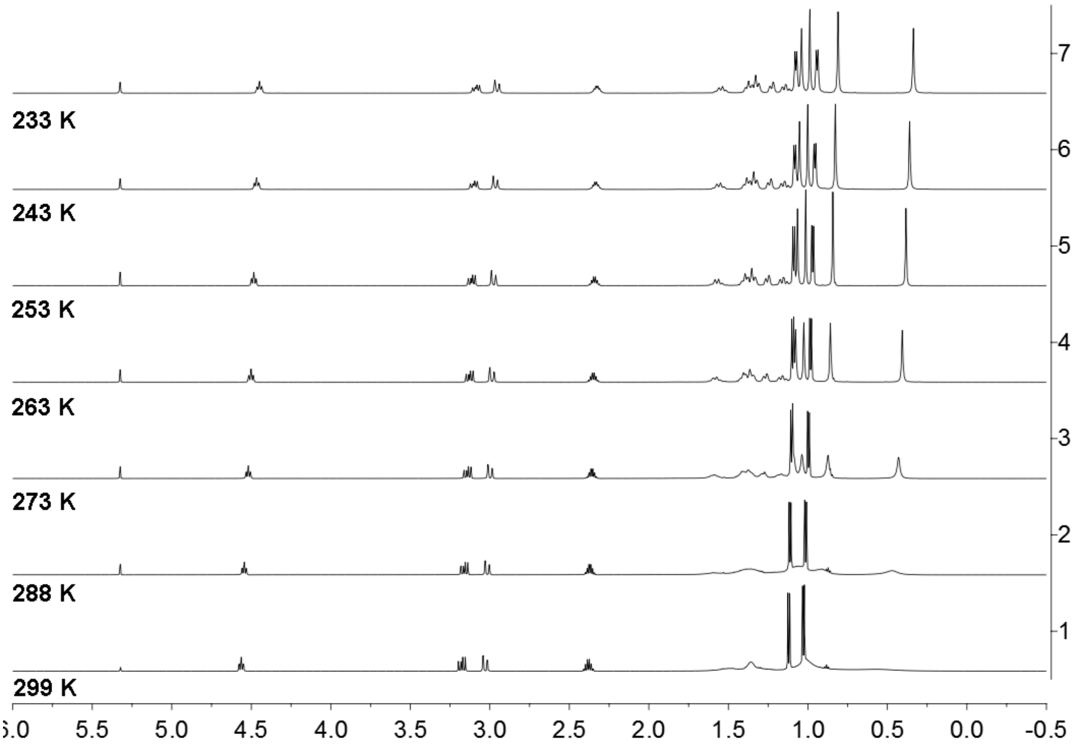

Figure S58. ^{1}H NMR (600 MHz, from 299 K to 233 K, CD_2Cl_2) spectra of compound **17b**.

Crystals suitable for the X-ray crystal structure analysis were obtained from a solution of compound **17b** in *n*-pentane at -35 °C.

X-ray crystal structure analysis of compound 17b: A colorless prism-like specimen of $C_{26}H_{28}BF_{10}N_3O_2$, approximate dimensions 0.078 mm x 0.092 mm x 0.182 mm, was used for the X-ray crystallographic analysis. The X-ray intensity data were measured. A total of 935 frames were collected. The total exposure time was 20.78 hours. The frames

were integrated with the Bruker SAINT software package using a narrow-frame algorithm. The integration of the data using a monoclinic unit cell yielded a total of 45157 reflections to a maximum θ angle of 25.02° (0.84 Å resolution), of which 4865 were independent (average redundancy 9.282, completeness = 99.9%, R_{int} = 10.41%, R_{sig} = 5.69%) and 4015 (82.53%) were greater than 2σ(F^2). The final cell constants of <u>a</u> = 11.4759(7) Å, <u>b</u> = 11.5926(7) Å, <u>c</u> = 20.7695(13) Å, β = 95.877(2)°, volume = 2748.6(3) $Å^3$, are based upon the refinement of the XYZ-centroids of 9919 reflections above 20 σ(I) with 5.008° < 2θ < 55.26°. Data were corrected for absorption effects using the multi-scan method (SADABS). The ratio of minimum to maximum apparent transmission was 0.863. The calculated minimum and maximum transmission coefficients (based on crystal size) are 0.9750 and 0.9890. The final anisotropic full-matrix least-squares refinement on F^2 with 385 variables converged at R1 = 9.45%, for the observed data and wR2 = 18.41% for all data. The goodness-of-fit was 1.200. The largest peak in the final difference electron density synthesis was 0.497 $e^-/Å^3$ and the largest hole was -0.328 $e^-/Å^3$ with an RMS deviation of 0.086 $e^-/Å^3$. On the basis of the final model, the calculated density was 1.487 g/cm^3 and F(000), 1264 e^-.

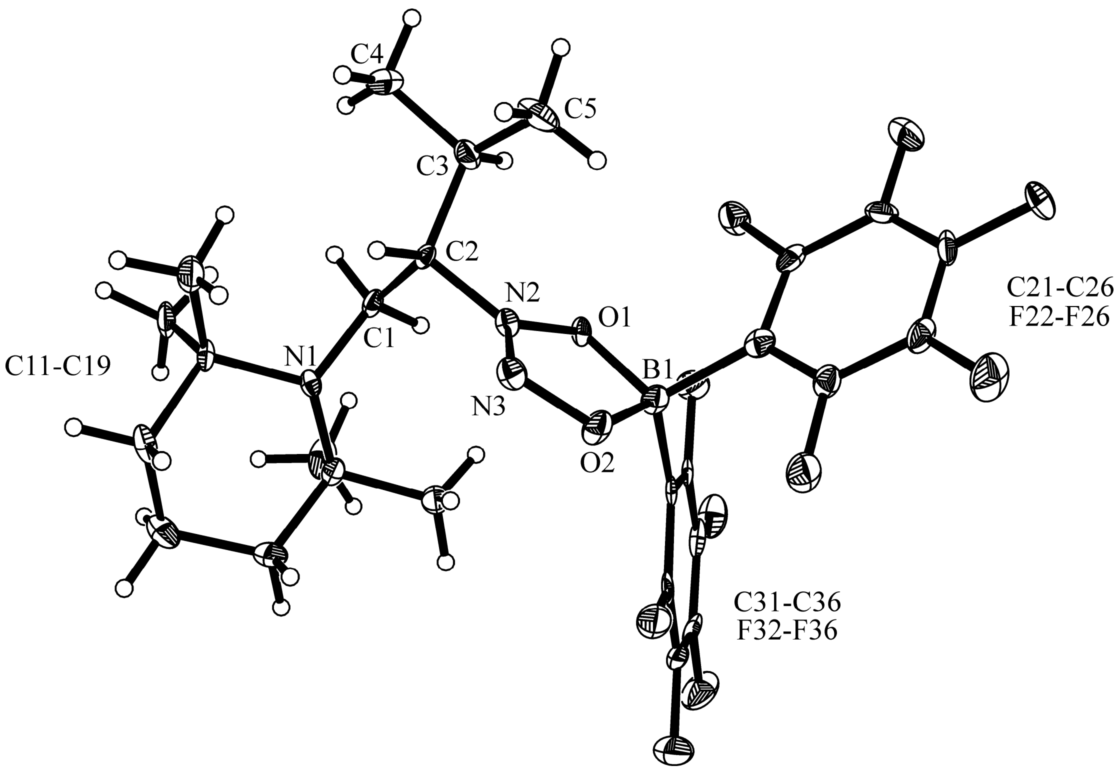

Figure S59. A view of the molecular structure of compound **17b**.

Synthesis of compound 26

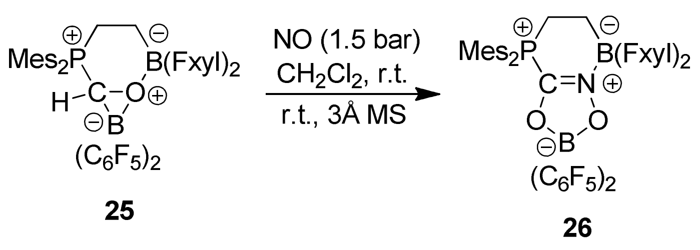

A solution of compound **25** (221.6 mg, 0.20 mmol) in CH_2Cl_2 (6 mL) containing molecular sieves (4Å, 400 mg, fine powder) was degassed by freeze-pump-thaw cycles (× 2). The mixture was carefully evacuated and exposed to NO gas (1.5 bar). After the reaction mixture was stirred at room temperature for 2 hours, it was filtered and the separated molecular

sieves were washed with CH_2Cl_2 (2 mL × 2). Then all volatiles were removed from the filtrate in vacuo and the residue crystallized from CH_2Cl_2/pentane to give compound **7** as a white solid. Yield: 106.8 mg, 0.094 mmol, 47 %.

Anal. Calcd. for $C_{49}H_{32}BF_{12}P$: C 51.75, H 2.84, N 1.23; Found: C 52.48, H 3.10, N 1.33.

Mp (DSC): 221 °C

^{1}H NMR (500 MHz, 299K, dichloromethane-d$_2$) δ = 7.81 (br, 2H, *o*-Fxyl), 7.72 (br, 1H, *p*-Fxyl), 7.08 (d, $^4J_{PH}$ = 5.2 Hz, 2H, *m*-Mes), 3.13 (m, 1H, PCH$_2$), 2.37 (s, 3H, *p*-Me^{Mes}), 2.22 (s, 6H, *o*-Me^{Mes}), 1.55 (dm, 1H, $^3J_{PH}$ = 32.8 Hz, BCH$_2$).

^{13}C{^{1}H} NMR (126 MHz, 299K, dichloromethane-d$_2$) δ = 154.5 (d, $^1J_{PC}$ = 104.5 Hz, C=N), 151.9 (br, *i*-Fxyl)^t, 147.1 (d, $^4J_{PC}$ = 3.1 Hz, *p*-Mes), 143.5 (d, $^2J_{PC}$ = 11.1 Hz, *o*-Mes), 133.3 (d, $^3J_{PC}$ = 12.4 Hz, *m*-Mes), 132.9 (br, *o*-Fxyl), 130.2 (q, $^2J_{FC}$ = 32.0 Hz, *m*-Fxyl), 124.5 (q, $^1J_{FC}$ = 271.5 Hz, CF$_3$), 120.4 (m, *p*-Fxyl), 113.5 (d, $^1J_{PC}$ = 82.1 Hz, *i*-Mes), 25.6 (d, $^1J_{PC}$ = 43.4 Hz, PCH$_2$), 23.2 (d, $^3J_{PC}$ = 5.4 Hz, *o*-Me^{Mes}), 21.4 (d, $^5J_{PC}$ = 1.5 Hz, *p*-Me^{Mes}), 16.7 (br, BCH$_2$)^a, [^{t} tentative assignment; ^{a} from the ^{1}H,^{13}C ghsqc NMR experiment; C_6F_5 not listed].

^{11}B{^{1}H} NMR (160 MHz, 299K, dichloromethane-d$_2$) δ = 11.2 ($\nu_{1/2}$ ~ 275 Hz), -3.8 ($\nu_{1/2}$ ~ 300 Hz).

^{10}B{^{1}H} NMR (54 MHz, 299K, dichloromethane-d$_2$) δ = 11.0, -4.0.

^{19}F NMR (470 MHz, 299K, dichloromethane-d$_2$) δ = -63.1 (s, 6F, CF_3), -136.0 (m, 2F, *o*-C_6F_5), -157.4 (t, $^3J_{FF}$ = 20.0 Hz, 1F, *p*-C_6F_5), -164.6 (m, 2F, *m*-C_6F_5), [$\Delta\delta^{19}F_{m,p}$ = 7.2].

^{31}P{^{1}H} NMR (202 MHz, 299K, dichloromethane-d$_2$) δ = 13.5 ($\nu_{1/2}$ ~ 3 Hz).

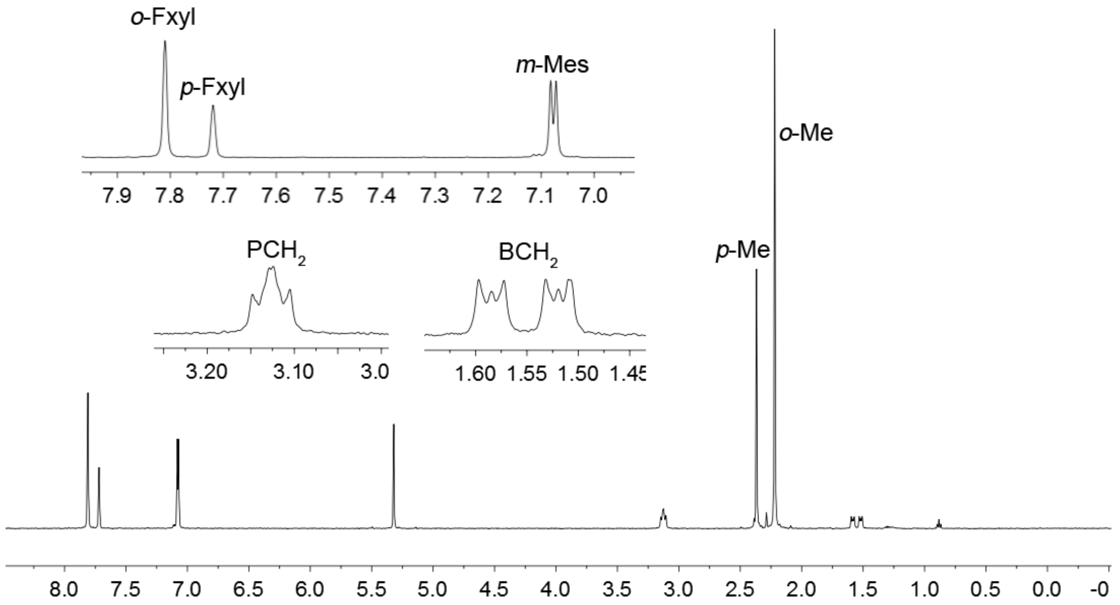

Figure S60. ^{1}H NMR (500 MHz, 299K, dichloromethane-d$_2$) spectrum of compound **26**.

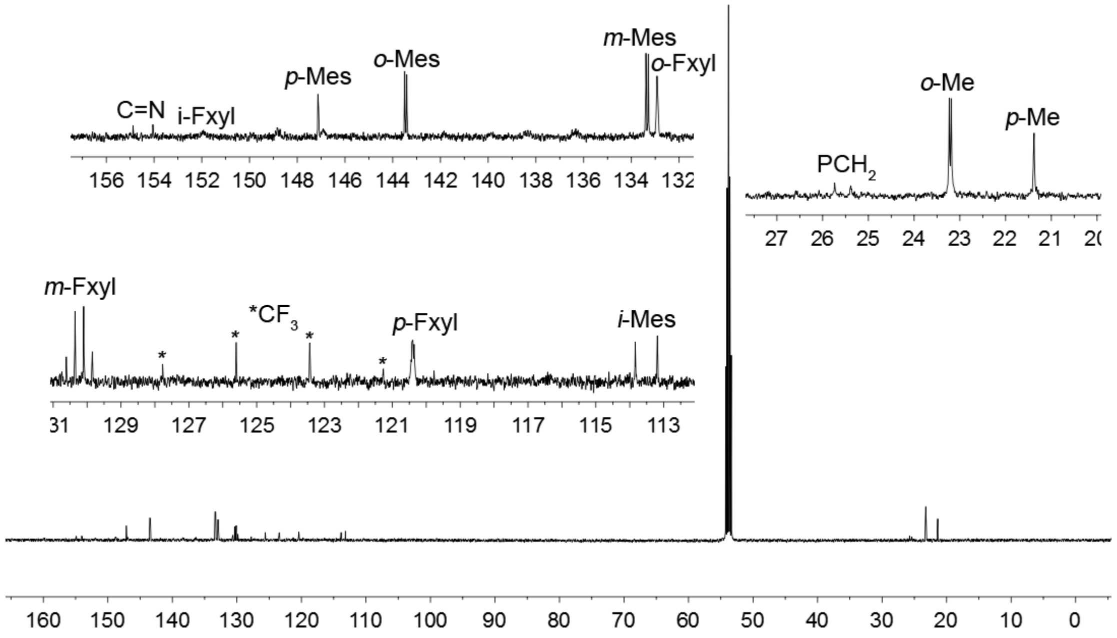

Figure S61. $^{13}C\{^{1}H\}$ NMR (126 MHz, 299K, dichloromethane-d_2) spectrum of compound **26**.

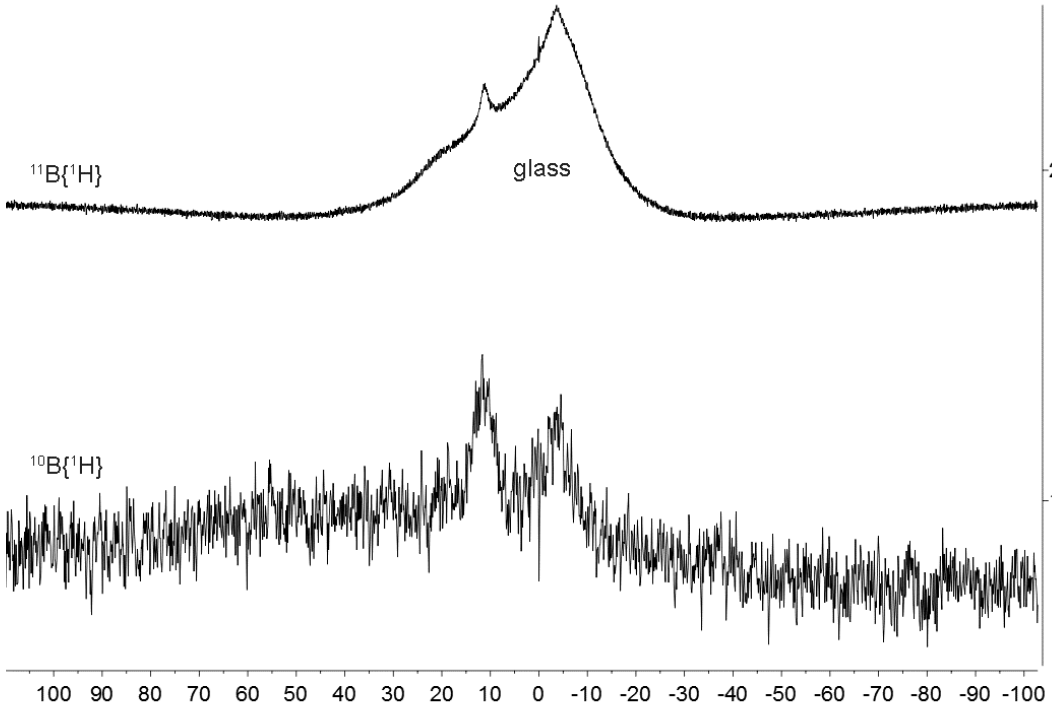

Figure S62. (2) $^{11}B\{^{1}H\}$ NMR (160 MHz) and (1) $^{10}B\{^{1}H\}$ NMR (54 MHz, 299K, dichloromethane-d_2) spectra of compound **26**.

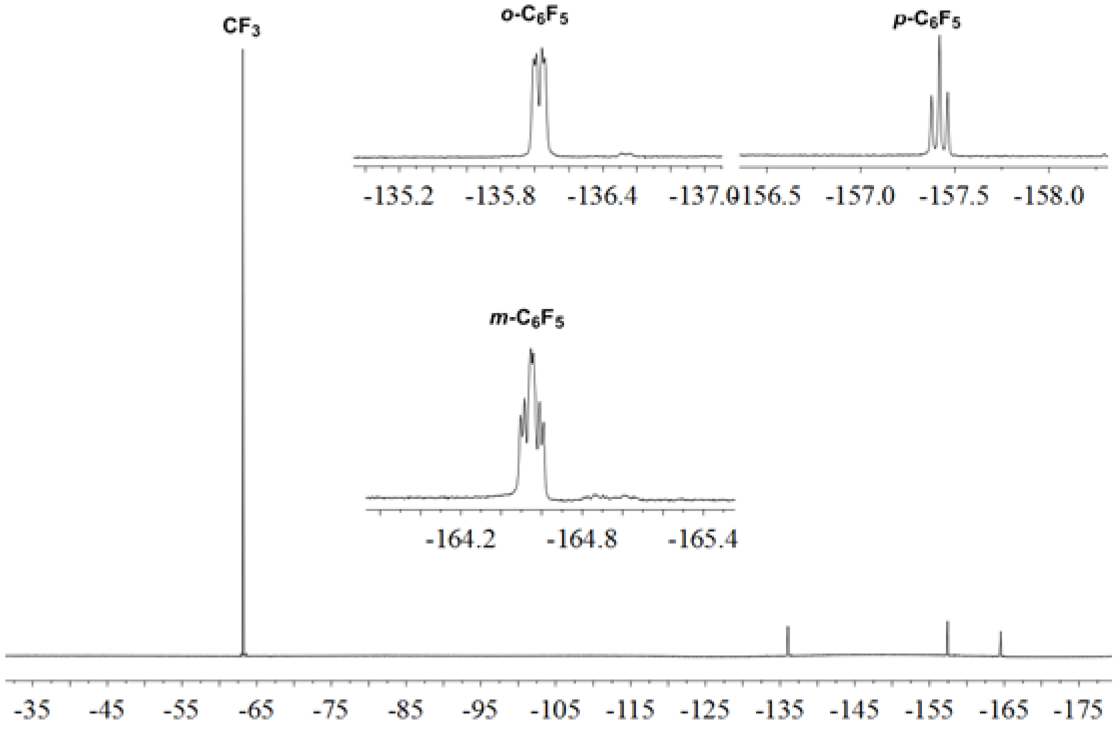

Figure S63. ^{19}F NMR (470 MHz, 299K, dichloromethane-d_2) spectrum of compound **26**.

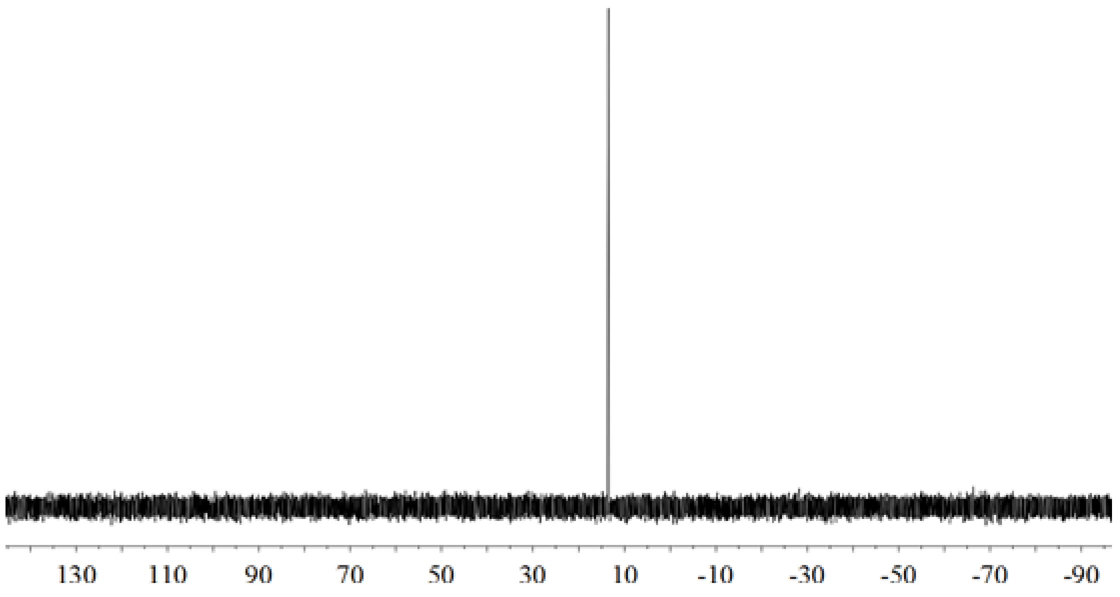

Figure S64. $^{31}P\{^{1}H\}$ NMR (202 MHz, 299K, dichloromethane-d_2) spectrum of compound **26**.

Crystals suitable for the X-ray crystal structure analysis were obtained by diffusion of pentane to a solution of the white solid in dichloromethane at -36 °C.

X-ray crystal structure analysis of compound 26: A colorless plate-like specimen of $C_{49}H_{32}B_2F_{22}NO_2P$, approximate dimensions 0.030 mm x 0.080 mm x 0.220 mm, was used for the X-ray crystallographic analysis. The X-ray intensity data were measured. A total of 1627 frames were collected. The total exposure time was 19.97 hours. The frames were integrated with the Bruker SAINT software package using a wide-frame algorithm. The integration of the data using a monoclinic unit cell yielded a total of 8883 reflections to a maximum θ angle of 65.08° (0.85 Å resolution), of which 8883 were independent (average redundancy 1.000, completeness = 96.2%, R_{int} = 11.49%, R_{sig} = 10.18%) and 5243 (59.02%) were greater than 2σ(F^2). The final cell constants of a = 12.2342(14) Å, b = 24.232(3) Å, c = 18.5607(19) Å, β = 100.311(7)°, volume = 5413.6(10) $Å^3$, are based upon the refinement of the XYZ-centroids of 7831 reflections above 20 σ(I) with 7.296° < 2θ < 133.0°. Data were corrected for absorption effects using the multi-scan method (SADABS). The ratio of minimum to maximum apparent transmission was 0.777. The calculated minimum and maximum transmission coefficients (based on crystal size) are 0.7360 and 0.9570. The final anisotropic full-matrix least-squares refinement on F^2 with 828 variables converged at R1 =

6.84%, for the observed data and wR2 = 18.73% for all data. The goodness-of-fit was 1.040. The largest peak in the final difference electron density synthesis was 0.304 $e^-/Å^3$ and the largest hole was -0.554 $e^-/Å^3$ with an RMS deviation of 0.076 $e^-/Å^3$. On the basis of the final model, the calculated density was 1.395 g/cm^3 and F(000), 2288 e^-.

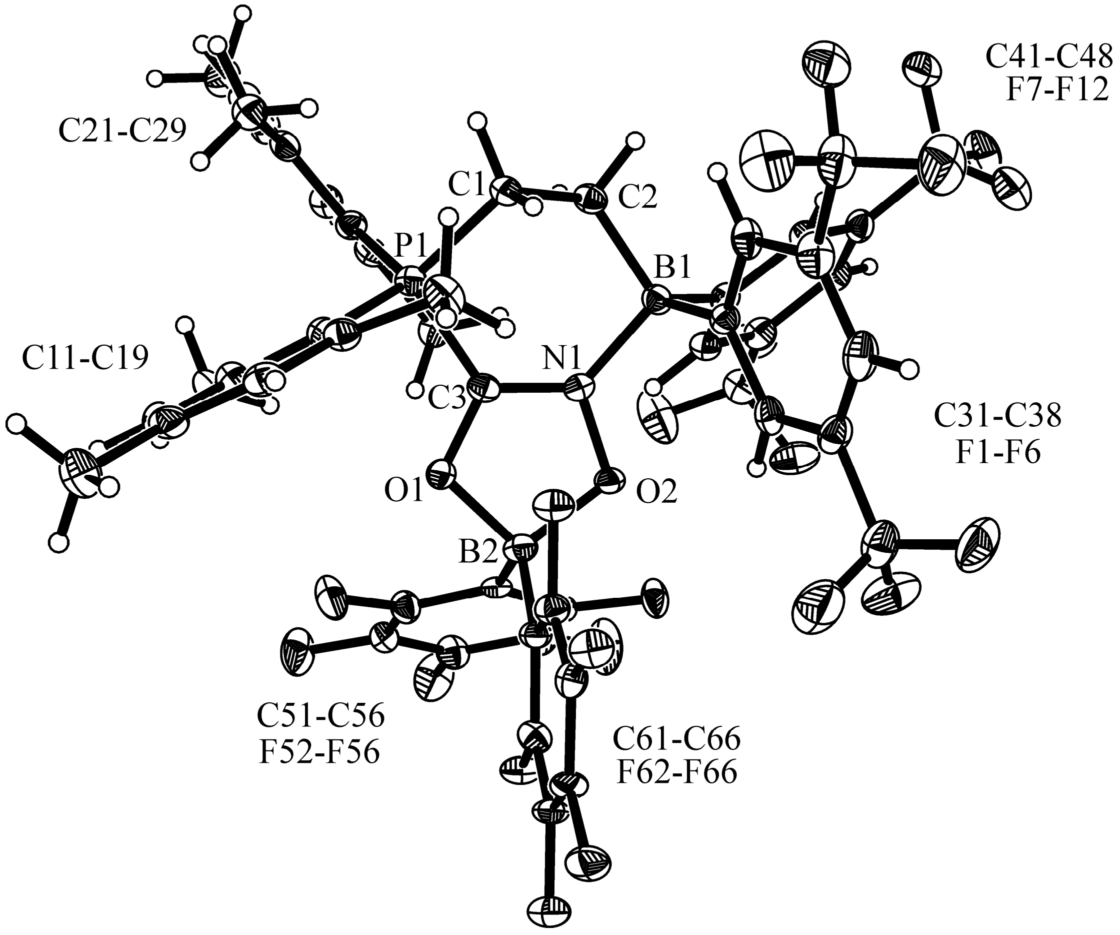

Figure S65. A view of the molecular structure of compound **26**.
